# The effects of manipulating levels of replication initiation factors on origin firing efficiency in yeast

**DOI:** 10.1371/journal.pgen.1008430

**Published:** 2019-10-04

**Authors:** Kelsey L. Lynch, Gina M. Alvino, Elizabeth X. Kwan, Bonita J. Brewer, M. K. Raghuraman

**Affiliations:** 1 Molecular and Cellular Biology Program, University of Washington, Seattle, Washington, United States of America; 2 Department of Genome Sciences, University of Washington, Seattle, Washington, United States of America; National Cancer Institute, UNITED STATES

## Abstract

Chromosome replication in *Saccharomyces cerevisiae* is initiated from ~300 origins that are regulated by DNA sequence and by the limited abundance of six *trans*-acting initiation proteins (Sld2, Sld3, Dpb11, Dbf4, Sld7 and Cdc45). We set out to determine how the levels of individual factors contribute to time of origin activation and/or origin efficiency using induced depletion of single factors and overexpression of sets of multiple factors. Depletion of Sld2 or Sld3 slows growth and S phase progression, decreases origin efficiency across the genome and impairs viability as a result of incomplete replication of the rDNA. We find that the most efficient early origins are relatively unaffected by depletion of either Sld2 or Sld3. However, Sld3 levels, and to a lesser extent Sld2 levels, are critical for firing of the less efficient early origins. Overexpression of Sld3 simultaneously with Sld2, Dpb11 and Dbf4 preserves the relative efficiency of origins. Only when Cdc45 and Sld7 are also overexpressed is origin efficiency equalized between early- and late-firing origins. Our data support a model in which Sld3 together with Cdc45 (and/or Sld7) is responsible for the differential efficiencies of origins across the yeast genome.

## Author summary

Eukaryotic chromosome duplication begins at sites called origins of replication along the chromosomal DNA. A conserved property of eukaryotic origins is that they vary in efficiency—the proportion of cells in a population in which they “fire”—and in the average time of activation within S phase, but the molecular details underlying this variation are not well understood. Previous work has shown that limiting concentrations of a set of conserved replication initiation proteins referred to as “SSDDCS” (Sld2, Sld3, Dbf4, Dpb11, Cdc45, and Sld7) are rate limiting for origin activation in budding yeast and other eukaryotes; combined overexpression of these proteins increases and/or advances origin firing. However, it remained possible that different factors affect different aspects of origin activation (e.g., timing vs. efficiency). Here, by depleting individual factors or by overexpressing sets of factors in budding yeast, we demonstrate that it is levels of Sld3, Cdc45 and/or Sld7 levels are primarily responsible for modulating the differences in relative origin efficiency and timing. This work gives further insights into what shapes the landscape of genome duplication.

## Introduction

One of the enduring mysteries in the field of eukaryotic DNA replication is the variability in initiation of DNA replication across a genome. Eukaryotes initiate DNA replication from multiple sites, known as origins, which are distributed along the length of each linear chromosome [1, reviewed in 2]. Early studies of DNA replication revealed that certain parts of the eukaryotic genome are replicated earlier in S phase than others, and that the large-scale organization of chromosomes, their underlying sequence and chromatin structure play a regulatory role in this variability [3, 4]. These differences in replication time arise from staggered origin firing during S phase with initiation occurring in a continuum from early to later in S phase [5, 6].

Not all potential sites of initiation are used in every cell: while some are used in nearly every cell cycle, others are used less frequently, and a few are almost never used in their native chromosomal context [7, 8]. This observed characteristic of what percent of cells in a population use a particular origin is what we refer to as origin “efficiency”. We note that this definition of efficiency is distinct from one of implied affinity or kinetic binding rates of origins for particular initiation factors [9]. The observed efficiency of an origin stems from a combination of an intrinsic ability of the origin to acquire all of the necessary replication factors to assemble bidirectional active helicases (which we have referred to previously as origin “competence” [10]), its genomic location relative to other origins and their times of activation. An origin that is fully competent to fire may be passively replicated by an incoming fork and therefore appear as less than 100% efficient. Because efficiency is the outcome of the interplay between origin competence and extrinsic variables such as location of nearby origins and their properties, measuring the competence of individual origins has been difficult. In broad terms, these two modes of variability in replication initiation, origin efficiency and origin firing time, are conserved from *Saccharomyces cerevisiae* to humans [6, 11]. The conservation of these features implies that plasticity in origin activation is biologically relevant, although the nature of its importance for genome function is not entirely clear.

**Fig 1:**
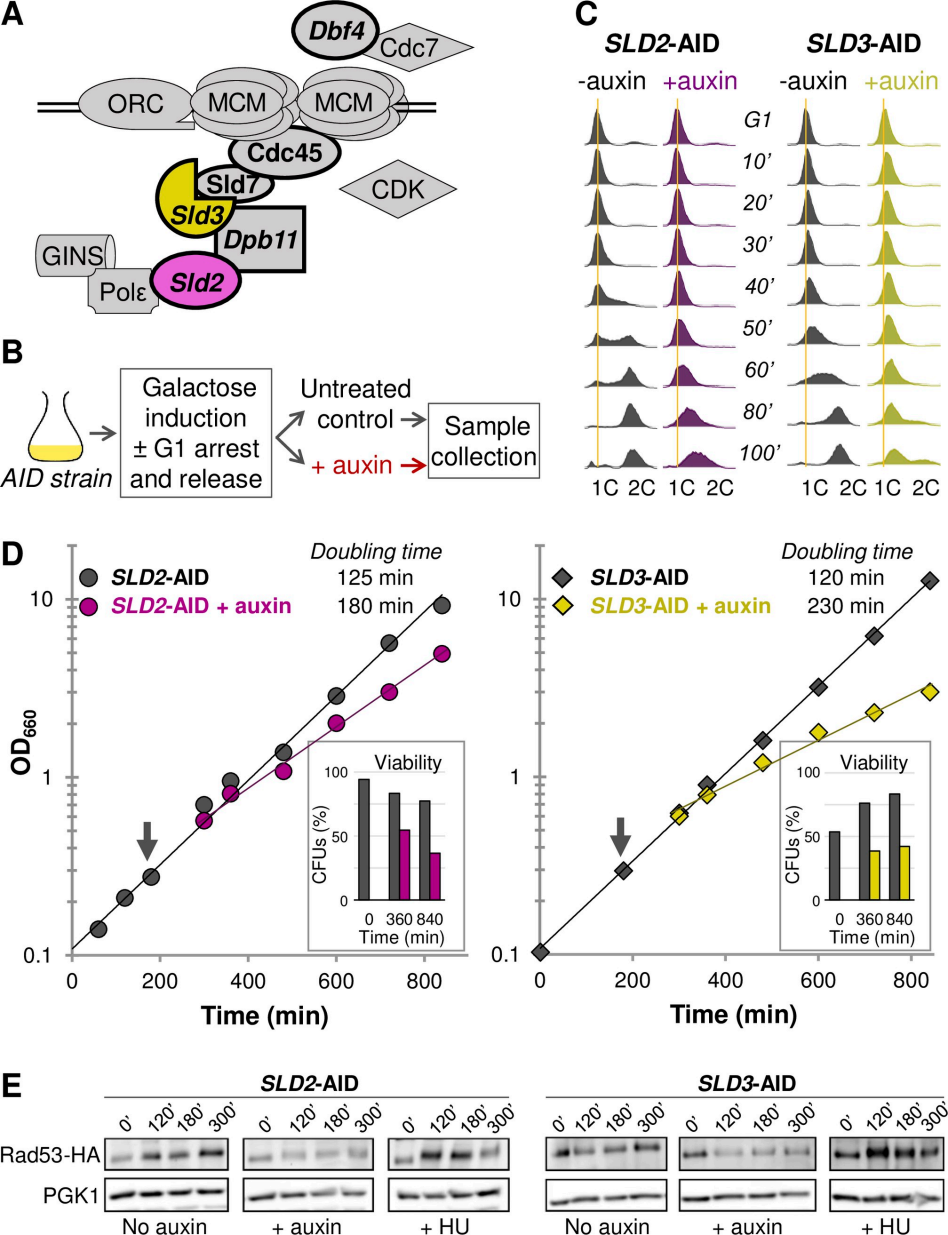
Initial phenotyping of Sld2- or Sld3-depleted cells. (A) Sld2 and Sld3, shown in purple and yellow, are two replication initiation factors thought to limit origin firing in *S*. *cerevisiae*. Here, they are shown with the assembling replisome. The other limiting initiation factors (Dbf4, Dpb11, Cdc45, and Sld7) are shown in bold. (B) General outline for experiments using the AID system to degrade a protein of interest. (C) Flow cytometry profiles for synchronized S phase in *SLD2*-AID and *SLD3*-AID. The vertical orange lines indicate 1C DNA. (D) Log phase growth of *SLD2*-AID and *SLD3*-AID. The arrow indicates when we divided the cultures and added auxin. Sld2 depletion increased the doubling time by 44%. Sld3 depletion increased the doubling time by 92%. The proportions of cells able to form colonies, i.e. the proportion of viable cells after being returned to no-depletion conditions, are shown for three timed samples. (E) Western blot for Rad53 phosphorylation in *SLD2*-AID and *SLD3*-AID strains containing HA-tagged Rad53. We collected samples at the indicated times after release into S phase with and without auxin induction. We treated the positive control with 200 mM hydroxyurea (HU) to achieve maximum Rad53 phosphorylation.

The proteins that recognize origin DNA and initiate replication at those loci are well conserved across eukaryotes; however, the molecular details have been most comprehensively studied in the budding yeast *S*. *cerevisiae* (reviewed in [12]). For an origin to fire, the origin DNA sequence is first bound by the origin recognition complex (ORC) which, through a set of interacting protein factors, coordinates the loading of the replicative MCM2-7 helicase during G1 [13–16]. In the transition from G1 to S phase, a series of initiation factors, a subset of which are illustrated in Fig 1A, associate transiently with the complex resulting in the assembly of the active replicative helicase known the CMG (Cdc45-MCM2-7-GINS) helicase [17, 18]. CMG assembly is considered the commitment step of replication initiation at origins and leads irreversibly to the establishment of bidirectional replisomes [12]. Variation in this crucial replisome assembly step is thought to determine time of origin firing and origin competence.

The assembly and activation of replisomes are regulated by two kinases, S-CDK (S-phase cyclin-dependent kinase; Cdc28 complexed with either Clb5 or Clb6) and DDK (Dbf4-dependent kinase; Cdc7 complexed with Dbf4) [19–23]. Clb5 and Clb6 have redundant roles in phosphorylating various replisome components; however, since Clb6 is expressed only at the beginning of S phase, late origin firing is dependent on Clb5 expression [10, 21, 24]. The processes that distribute CDK and DDK to specific origins are not well understood, although the kinetochore protein Ctf19 has been shown to recruit DDK to centromeric origins to ensure their early firing [25], and forkhead proteins Fkh1 and Fkh2 have been implicated in recruitment of DDK to the vicinity of origins that contain forkhead binding sites [26, 27]. The underlying DNA sequences and chromatin context at origins seem to have the greatest influence on regulation of initiation [3, reviewed in 9, 28, 29].

The discovery that six specific replication initiation factors (Sld2, Sld3, Dpb11, Dbf4, Sld7 and Cdc45—the “SSDDCS” factors) are produced at concentrations significantly lower than the pre-replication complex and replisome components suggested that they may be rate-limiting for origin initiation [30]. When Mantiero et al. [30] overexpressed four proteins simultaneously (Sld2, Sld3, Dpb11 and Dbf4; “SSDD”), the temporal firing of origins was compressed, and a subsequent study revealed that origin efficiency increased genome-wide when Cdc45 and Sld7 were overexpressed along with the SSDD proteins [31]. Independently, Tanaka et al. [32] found that overexpression of just Sld3, Sld7, and Cdc45 could advance the time of activation for late origins. These findings suggest that the varying ability of different parts of the genome to efficiently recruit the limiting factors results in the pattern of origin use observed during DNA replication. From their observations, Mantiero et al. [30] developed a model whereby genome features near origins determine how well origins are able to recruit the limiting SSDD factors, with the earliest and most efficient origins being the most competitive for recruiting those factors. Since each of these four proteins is required only for the initiation phase of DNA replication [12, 33–36], Mantiero et al. [30] proposed that as early and/or efficient origins become active and release the limiting factors from the assembling replisome, those factors are recycled to origins that are less competitive for recruiting SSDD and thereby promote origin firing at later and/or more inefficient origins. The SSDD proteins are all essential [12] as is Cdc45, which is also is the only limiting factor to travel with the replication fork [18]. Sld7 is the only non-essential limiting factor in yeast [37].

Collart et al. [38] demonstrated that the same limiting factors restrict origin firing during early embryonic development in *Xenopus laevis*. At the mid-blastula transition (MBT), developing embryos transition from relying on stores of maternally-derived transcripts to activating embryonically-driven transcription [39, 40]. The transcriptional activation of the genome results in drastic changes in embryonic cell cycles—from rapid, synchronous divisions with fast S phases and an almost complete lack of gap phases to slower, asynchronous divisions with G1 and G2 phases and a slowed S phase featuring greatly reduced origin activity. Collart et al. [38] demonstrated that the reduction in DNA replication initiation at the MBT depends upon titration of the orthologous SSDD factors. By simultaneously increasing the concentrations of the SSDD factors, they found that replication initiation was not reduced, that the cells continued to divide rapidly, and that the embryos failed to develop. These observations suggest that the establishment of variation in origin activity is an essential feature of embryogenesis.

While the “limiting factors” model for variable origin firing time and efficiency is an attractive one, there are still many questions regarding the mechanisms of how these factors associate with origins. Do some origins have a more favorable underlying DNA sequence, a more advantageous chromatin conformation, and/or reside in a prime nuclear compartment that promotes their advanced time of firing or their efficiency? Do some of the limiting factors contribute to efficiency while others contribute to the temporal aspect of origin firing?

For our study, we have explored the role that limiting factors play in determining origin efficiency and/or firing time—specifically we wondered whether further reducing the levels of these limiting factors would reveal specific regions of the genome or classes of origins as being particularly vulnerable to the reduced availability of the factor. One feature that influenced our work was the observation that even when the SSDD subset of limiting proteins were overexpressed in yeast, there were still differences in time of replication among the origins assayed [30]. This observation, coupled with the knowledge that each of the limiting factors interacts with the replisome at different times and with different components of the complex [12, 34, 36, 41, 42], suggested that the abundance of one or more of these factors may establish the replication program through preferential origin choice. Because sets of factors had to be expressed simultaneously to advance replication kinetics [30], the specific role of any of these factors independent of the others was not clear. Furthermore, while overexpression of all six factors largely reduced the differences in observed efficiency between origins [31], it was not clear whether overexpression of just SSDD would have the same effect. It was also not evident to what extent the increase in observed efficiency was the consequence of a reduction in passive replication of origins due to increased initiation across the genome. Therefore, we reasoned that comparing the outcome of depleting individual limiting factors to those resulting from overexpression of SSDD or SSDDCS would allow us to query the roles of different initiation factors in origin choice.

Accordingly, we introduced an auxin-inducible degron (AID; [43, 44]) tag on Sld2 or Sld3 and assessed changes in cell cycle progression, genome stability, and chromosome replication after inducing protein degradation. We find that reduced abundance of either Sld2 or Sld3 results in viability defects, and that Chromosome XII is uniquely unstable as a consequence of the failure to complete rDNA replication. Overall the rDNA phenotypes of Sld2- and Sld3-depleted cells are similar, with the Sld3-targetting strain having a more severe phenotype. At the level of individual single copy origins we find that all origins that usually fire early in S phase still do so but some origins fire with reduced efficiencies. No regions of the genome move from early-replicating to late-replicating as a consequence of Sld2 or Sld3 depletion. However, we find that those origins that are inherently less efficient are more severely affected than by Sld3 depletion than by Sld2 depletion. If Sld3 were the only factor that limits the efficiency of early origins, then overexpression of Sld3 alone or in the context of other limiting factors would equalize origin firing efficiency. We examined early origin efficiency during overexpression of Sld3 in concert with Sld2, Dbf4 and Dpb11 (SSDD) and find that the variability in levels of origin efficiency is maintained. However, additional overexpression of Sld7 and Cdc45 effectively removes the distinction of early and late origins and equalizes their relative efficiencies. We conclude that the SSDD overexpression improves overall origin firing efficiency but largely does not remove the distinction between early and late origins. Therefore, both relative efficiency and time of firing are likely established or reinforced by the availability of Cdc45 and/or Sld7.

## Results

### Independent depletion of two limiting replication initiation factors slows S phase progression, reduces growth rate, and impacts cellular viability

In this study, our goal was to manipulate the abundance of the limiting SSDDCS factors and then examine DNA replication initiation genome-wide to test whether those factors contribute to time and/or efficiency of origin activity. Initially, using the 3x-mini-AID version of the auxin inducible degron (AID) system [43, 44], we created strains with a C-terminal auxin-inducible degron tag on Sld2 or Sld3, referred to as *SLD2*-AID and *SLD3*-AID. Because Sld2 and Sld3 associate sequentially with the replisome during the cell cycle and interact with distinct proteins within the replication complex (Fig 1A) [12, 32, 41], we expected that focusing on those factors could reveal insights into how the abundance of these proteins contribute uniquely to variable replication initiation. Note that henceforth, by “induced” *SLD2-*AID or *SLD3-*AID we mean depletion of Sld2 or Sld3, respectively, while “uninduced” indicates the control, no-depletion condition.

We tested S-phase progression in *SLD2*-AID and *SLD3*-AID by measuring DNA content with flow cytometry. After arresting cells in G1 phase and inducing expression of the plant-derived E3 ubiquitin ligase with galactose, we induced degradation of Sld2 or Sld3 by adding 0.5 mM auxin to one half of the culture and leaving the other half as an uninduced control (Fig 1B). Uninduced *SLD2*-AID and the untagged AID parent (plus or minus auxin; S1 Fig) were indistinguishable in their progression through and completion of S phase by 80 minutes. *SLD3*-AID completed S phase by 80–100 minutes but entered S phase more slowly than the *SLD2*-AID strain (Figs 1C and S1). The S phase delay in uninduced *SLD3*-AID may be the consequence of the degron tag interfering with Sld3 function (see below). However, the addition of auxin (i.e., inducing degradation) severely impeded progress through S phase for both *SLD2*-AID and *SLD3*-AID: by 100 minutes neither strain had reached 2C DNA content, again with *SLD3*-AID lagging behind *SLD2*-AID (Fig 1C). Since the Sld2- or Sld3-depleted cells did enter S phase, we concluded that replication initiation was reduced but not entirely inhibited by degron-induced protein degradation. Furthermore, we suspected that the more pronounced S phase delay resulting from Sld3 depletion compared to the delay caused by Sld2 depletion may reflect a key difference between the two proteins. Sld3 is less abundant than Sld2, even before depletion, so reducing its abundance further may suppress origin firing more severely [30, 45].

To determine whether the increased S phase duration we observed in the Sld2- and Sld3-limited cells impacted growth rate and/or cellular viability, we compared log-phase growth of the two degron strains. Doubling times increased for both *SLD2*-AID and *SLD3*-AID when the target proteins were degraded (Fig 1D). In agreement with the more severe S phase phenotype observed by flow cytometry, the Sld3-depleted cells exhibited a greater increase in doubling time compared to cells depleted for Sld2 (Fig 1D). In the final timed sample, both strains had experienced an approximately 40% decrease in viability compared to the uninduced control as assayed by colony-forming ability of cells (Fig 1D). While auxin treatment did slightly slow growth in the AID parent strain, the difference was not due to decreased viability (S1B Fig).

One explanation for the rapid loss of viability in the auxin-induced cultures is that the S phase checkpoint is activated in response to initiation factor depletion, preventing the cells from completing the cell cycle and eventually leading to cell death [46, 47]. To test for checkpoint activation, we HA-epitope tagged Rad53 in the AID strains, then tested for phosphorylation of Rad53 during synchronous S phase. We detected modest Rad53 phosphorylation in the auxin-treated samples over the five-hour time course (Fig 1E). Adding auxin to the AID parent did not lead to Rad53 phosphorylation (S1D Fig). Therefore, although we cannot rule out a possible contribution of Rad53 checkpoint activation to the overall decrease in viability upon Sld2 or Sld3 depletion, it does not appear to be a major cause of reduced viability.

### Chromosome XII is specifically destabilized by Sld2 or Sld3 depletion

The rapid loss of viability and the moderate activation of the S phase checkpoint when Sld2 or Sld3 are depleted suggested that genome stability might be compromised. Because robust activation of the S phase checkpoint requires that cells establish a threshold of active replication forks [48, 49] we hypothesized that not all Sld2- or Sld3- depleted cells in the population reached this threshold and, as a result, some cells were entering mitosis with partially-replicated chromosomes causing double-stranded breaks, mitotic catastrophe, and cell death [50].

We used contour-clamped homogeneous electric field (CHEF) gel electrophoresis to assess chromosome stability during and after a synchronous S phase in *SLD2-*AID and *SLD3*-AID. Branched chromosomes formed during replication are unable to migrate from the wells and, therefore, the hybridization signal for individual chromosomes at their characteristic positions on CHEF gels is reduced while signal in the well is increased [51, 52]. If chromosome replication were slowed, we would expect increased hybridization at the well and for well hybridization to be present for a longer period of time. If partially-replicated chromosomes break from mitotic catastrophe, we would expect diffuse hybridization signal below the position of the intact chromosome band.

**Fig 2:**
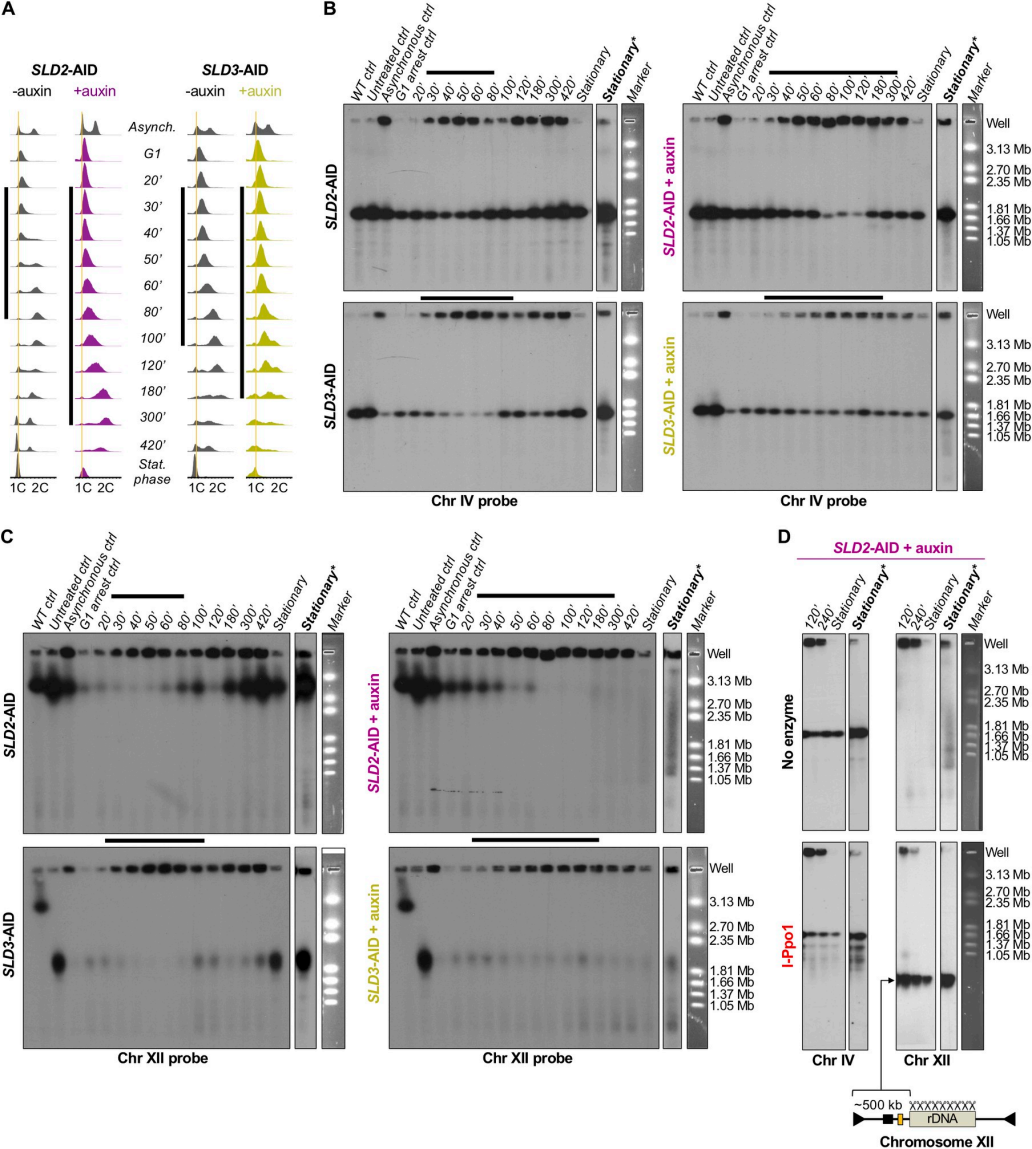
CHEF gel analysis of chromosome stability during Sld2- or Sld3-depleted S phase. (A) Flow cytometry profiles for the synchronized *SLD2*-AID and *SLD3-*AID strains, as well as asynchronous and stationary phase cells, collected for CHEF gel analysis. The vertical black bar to the left of each set of profiles shows the estimated duration of synchronous S phase. We continued to collect samples until synchrony was lost after T100 in the uninduced cells. Note that in the auxin-treated samples there is a small peak at 1C DNA in the later samples suggesting that the cells eventually divided. (B) Southern blot of the CHEF gels probed for Chr IV (*GAL3* probe). The horizontal black bars correspond to the extent of S phase determined from the flow cytometry profiles shown in (A). The EtBr-stained gel images to the right show *Hansenula wingei* chromosomes (sizes in Mb) as markers for electrophoretic mobility. Hybridization at the top of each lane is from DNA trapped in the wells. The “WT control” is a stationary phase W303 sample. The “untreated control” is from stationary phase cells that were neither galactose induced nor auxin treated. The “asynchronous control” is from cells before α factor arrest. “Stationary” is a saturated culture from cells that grew past 420 minutes to stationary phase. The images of the “stationary” sample indicated by bold type and an asterisk are longer film exposures to reveal subchromosome-sized DNA fragments. (C) The blots in panel B were re-probed with a single copy sequence on Chr XII (*CDC45*). (D) Chr IV- and Chr XII-probed Southern blots of auxin-induced, asynchronous *SLD2*-AID strain samples with and without I-Ppo1 restriction enzyme treatment. The illustration of Chr XII below the blots indicates where I-Ppo1 cuts within the rDNA on Chr XII and the predicted size for the fragment to the left of the rDNA. The yellow rectangle shows the approximate location of *CDC45*.

We synchronized *SLD2*-AID and *SLD3*-AID cultures in G1 and released them into S phase with and without auxin. We monitored cell cycle progression by flow cytometry and collected samples at intervals for 420 minutes (Fig 2A). Following the 420-minute sample collection, we allowed the remainder of the culture to grow to stationary phase overnight before recovering the final sample. We performed the same experiment using the AID parent strain (S1A and S2 Figs). All timed samples were embedded in agarose plugs and chromosomes examined by CHEF gel electrophoresis.

We probed the Southern blots of the CHEF gels for single-copy sequences on individual chromosomes (Chr IV in Fig 2B; blot images and quantification of chromosomes XV, X and III in panels F-I of S2 Fig). The WT control and untreated control samples from stationary phase (non-cycling) cells had little hybridization to the wells (Fig 2B). In the asynchronous samples, we observed more Chr IV signal in the well, reflecting the higher proportion of S phase cells (Fig 2B). The fraction of Chr IV signal migrating at its normal location was reduced as cells entered S phase and the well signal increased (Fig 2B). By T80—T100, we found the majority of signal returned to the expected Chr IV position and signal at the well was reduced (Fig 2B). The period between T30 and T80 marks the duration of replication for Chr IV (Fig 2B) and corresponds well with the estimated S phase length we determined by flow cytometry (Fig 2A, left). We quantified the well signal as a proportion of total chromosome signal to highlight the changes in well-retention over the course of S phase (panels A and B in S2 Fig). At the latest times (T180 –T420), when the uninduced cells had lost synchrony but were still in log-phase growth, the ratio of signal from partially-replicated and fully-replicated chromosomes was constant (Figs 2B and S2B).

In response to depletion of Sld2 or Sld3, the distributions of hybridization signal in the well vs. the signal from full-length Chr IV (Figs 2B and S2B) were in agreement with the flow cytometry analysis, indicating that Chr IV takes longer to finish replication when Sld2 or Sld3 are depleted. The accumulation of Chr IV signal in the wells was delayed (T30 –T180 in Figs 2B and S2B), and the maximum well signal occurred between T80 –T100, compared to T50 –T60 in the control (Figs 2B and S2B). We did not see evidence of random breakage for Chr IV in the timed or the stationary phase samples even after long exposures of the blot (Fig 2B). Additionally, we saw no differences in Chr IV migration depending on the presence of auxin in the AID parent strain control (S2C Fig). We conclude that Chr IV replication takes proportionately longer when either Sld2 or Sld3 is depleted, but replication of that chromosome appears to be complete since there is no evidence of breakage as the cells enter mitosis. Thus, Chr IV is not destabilized by depletion of either Sld2 or Sld3. The same conclusions can be made from our analyses of chromosomes XV, X, and III (panels F-I in S2 Fig). In contrast, the pattern of replication and stability of chromosome XII were strikingly different.

In uninduced *SLD2*-AID and in the AID parent (with or without auxin), the migration pattern of Chr XII was similar to that of Chr IV (Fig 2C, top left; S2E Fig). As cells entered S phase, the majority of Chr XII signal remained in the well and returned to its full-length position at the end of S phase in agreement with the S phase kinetics (Fig 2A, left). There was generally more Chr XII signal trapped in the well in all timed samples including in the AID parent (Fig 2C top left; panels D and E in S2 Fig), presumably due to its greater length (~twice the length of Chr IV) and to unresolved branched replication or recombination intermediates within the rDNA locus (Ide et al., 2007). In contrast, upon depletion of Sld2, Chr XII became trapped in the well as the cells entered S phase and did not return to its full-length position in the gel (Fig 2C). By the time the culture reached stationary phase, roughly half of the Chr XII signal was in subchromosomal-sized fragments (Fig 2C, top right). It appears that Chr XII, in response to Sld2 depletion, never completed replication and, as cells entered mitosis, was fragmented.

*SLD3*-AID cells had similar problems maintaining Chr XII. First, we noted that Chr XII in the *SLD3*-AID strain is more than 1.5 Mb shorter that the parental wild type strain (Fig 2C, bottom left). Because *SLD3*-AID is haploid, the reduction in size can only be explained by a loss of rDNA repeats. We estimate that there are ~250 rDNA repeats in the *SLD2*-AID strain vs. ~90 copies in the *SLD3*-AID strain. Consistent with the duration of S phase (Fig 2A), Chr XII signal persisted in the well longer in untreated *SLD3*-AID relative to *SLD2*-AID, potentially reflecting compromised function of degron-tagged Sld3 (Figs 2C and S2E). But by T100 in uninduced *SLD3-*AID, full length Chr XII returned to its position in the gel (Fig 2C). Post Sld3 depletion, most of the Chr XII signal remained in the well and appeared as subchromosomal fragments in the stationary phase culture (Fig 2C). The presence of small amounts of full-length Chr XII in the later samples (T100 –T300 in Figs 2C and S2E) likely originated from the subpopulation of cells that remained in G1 (Fig 2A, far right).

The persistence of Chr XII signal in the well followed by the appearance of subchromosomal fragments suggests that only Chr XII is unable to complete replication before the onset of anaphase resulting in chromosome-specific breakage. Our additional finding that, even without auxin treatment, *SLD3*-AID is unable to maintain the normal ~250 copies of the rDNA repeat suggests that a problem resides specifically with rDNA replication. If the problem were specific to the rDNA and not widespread across Chr XII, we would expect that the branched DNA that prevents Chr XII from migrating in the gel would be present only within the rDNA itself and not in the flanking portions of the chromosome.

To test the possibility that branched intermediates were restricted to the rDNA, we digested agarose-embedded DNA with I-Ppo1—a restriction enzyme that cleaves in each rDNA repeat but nowhere else in the yeast genome [53, 54]—and then determined whether the unique portions of Chr XII were able to enter the gel. If incomplete replication of the rDNA causes persistent branched structures on Chr XII, then cutting the chromosome within the rDNA should release the fully-replicated regions on either side of the rDNA and allow those arms of the chromosome to migrate into the gel. Probing the Southern blot for sequences outside of the rDNA would reveal whether the non-rDNA portions of Chr XII are able to migrate after the rDNA repeats are digested. Accordingly, we repeated the CHEF gel collection, this time using asynchronous cultures of the *SLD2*-AID strain and collecting cells 120 minutes, 240 minutes after adding auxin, and after growth to saturation. Flow cytometry confirmed an enrichment of S phase cells in the 120- and 240-minute samples (S3 Fig).

I-Ppo1 treatment of the 120- and 240-minute samples restored migration of the leftmost portion of Chr XII (Fig 2D). In the stationary phase sample, the Chr XII region to the left of the rDNA migrated as a discrete band of the expected size after I-Ppo1 digestion, suggesting that chromosome breakage occurred specifically in the rDNA (Fig 2D). The retention of Chr XII in the CHEF gel wells and the release of the hybridization signal by I-PpoI cleavage suggest that the specific fragmentation of Chr XII and the reduction in viability are the consequence of incomplete replication of the rDNA when Sld2 or Sld3 are further limited.

### Depletion of Sld2 or Sld3 reduces firing efficiency of the high copy rDNA origin of replication

Since Sld2 and Sld3 are both required for assembly of the replisome at origins, we reasoned that the rDNA replication problem might occur during initiation—either reduced efficiency of origin activation or delayed activation, such that replication of the locus cannot be completed in a timely manner. Each rDNA repeat contains a potential origin of replication within the non-transcribed spacer that lies between the 5’ ends of the divergently transcribed 35S and 5S genes (reviewed in [55]). On a recombinant plasmid, the rDNA origin is inherently inefficient, a property that is attributed to a single nucleotide replacement within the origin consensus sequence [56–58]. The combination of this SNP, silencing by Sir2 and other chromatin features unique to the rDNA, and the variable 35S transcriptional activity found across the rDNA array is thought to limit the origin firing density of the rDNA in its native context to about one origin in every 3–5 repeats [7, 48, 59–61].

**Fig 3:**
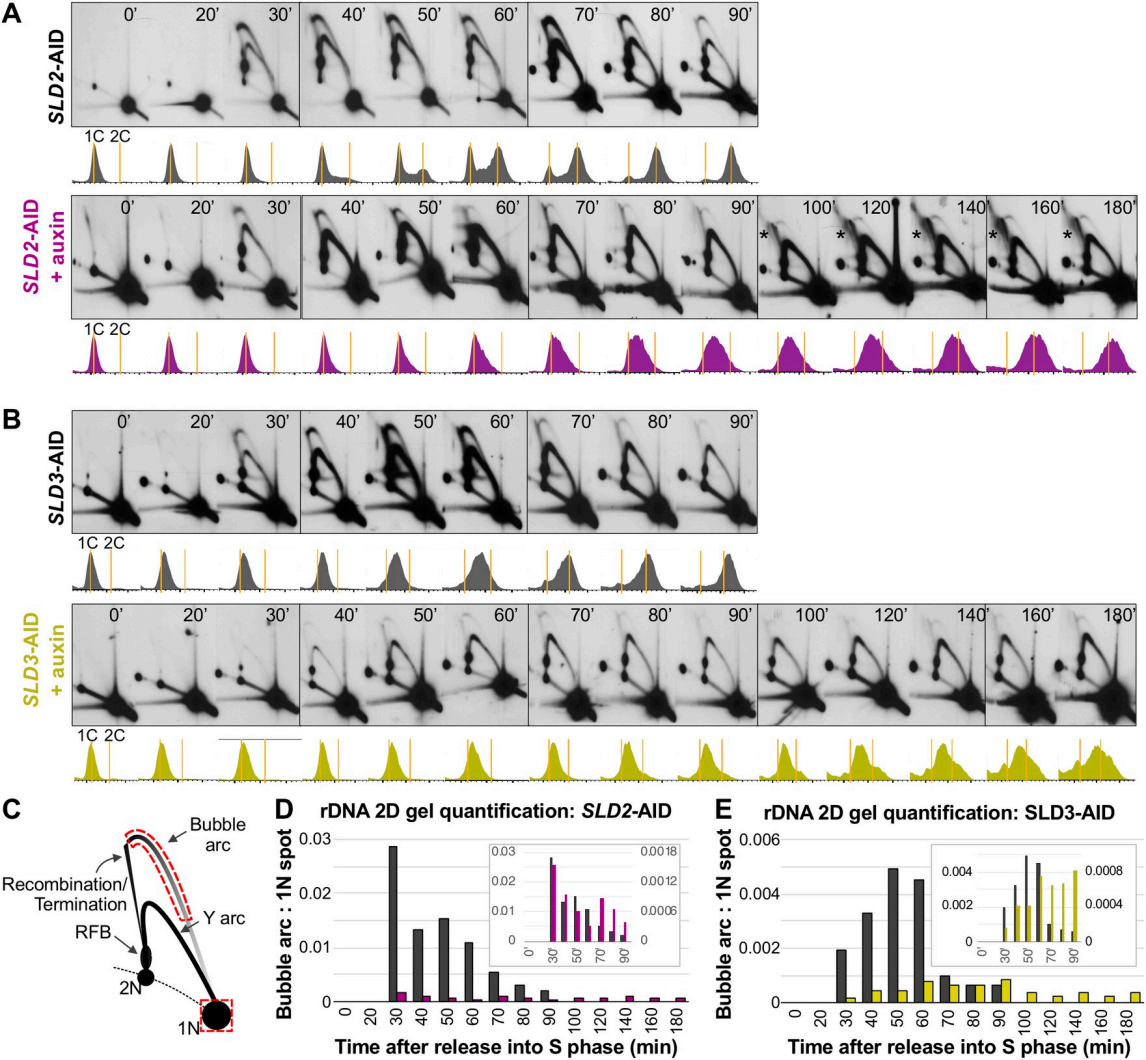
2D gel analysis of the rDNA origin of replication during synchronous S phase. (A) *SLD2*-AID rDNA 2D gels. The resulting Southern blots of NheI-digested genomic DNA were probed for the non-transcribed spacer region, *NTS2*, which contains the potential rDNA origin. The time relative to release from α-factor arrest is indicated in the top corner of each panel. Flow cytometry profiles corresponding to each timed sample are below each panel. Orange lines mark cells with 1C and 2C DNA content. The asterisks highlight the recombination/termination structures. (B) *SLD3*-AID rDNA 2D gels. Sample collection times and FACS profiles are labeled as in (A). (C) Illustration of an rDNA 2D gel and quantification strategy. The red boxes indicate the portions of the bubble arc and the 1N spot used to determine the bubble:1N ratio. (D) Quantification of the timed *SLD2*-AID rDNA 2D gels for uninduced (black) and induced (mulberry) samples. We did not calculate values for T0 and T20 since there was so little bubble arc hybridization signal in those samples. The inset displays the same data on a double-Y plot to highlight differences in initiation kinetics of the rDNA origin depending on Sld2 abundance (left axis = uninduced, black; right axis = induced, mulberry). (E) Quantification of *SLD3-*AID rDNA 2D gels as described in (D). Uninduced, black; induced, olive.

We assessed rDNA origin activity by examining the rDNA repeats, collapsed as a single restriction fragment, using 2D gel electrophoresis. The replication intermediates from the collection of identical restriction fragments are branched molecules that contain either a bubble shape (indicating initiation) or a Y shape (indicating passive replication). We collected samples from asynchronous, G1-arrested, and S phase-timed samples for uninduced and induced *SLD*2-AID and *SLD3*-AID cells then analyzed replication intermediates by 2D gel for the NheI restriction fragment containing the rDNA origin (Fig 3A and 3B, respectively). A decrease in probability of initiating at the rDNA origin would cause a reduction in replication bubble signal relative to replication fork signal on the Southern blot of the 2D gel, whereas a delay in activation would be manifested as a difference in the time at which bubble structures first become apparent when compared to the control condition. The uninduced cells show initiation beginning by T30 and falling to nearly undetectable levels by T90, which is when cells are at or near 2C DNA content. We quantified the bubble:1N ratio for each timed sample (Fig 3C–3E). The signal from replication bubbles from uninduced *SLD2*-AID peaked at T30 and gradually diminished as the cells neared 2C DNA content (Fig 3D). For uninduced *SLD3*-AID, the bubble signal peaked later (T50-T60) and declined to barely detectable levels by T90 (Fig 3E).

Sld2-depleted cells had reduced bubble:1N ratios throughout S phase with maximum initiation, ~5% of that in the uninduced culture, occurring most robustly at T30 and declining thereafter (Fig 3D). Initiation continued at a low level throughout the lengthened S phase (Fig 3A and 3D) and the most prominent form of replication intermediate was from passive replication (Y-arcs). Between 100–180 minutes, termination/recombination structures arising from the replication fork barrier (RFB) and/or 2N position became prominent, indicating that replication forks from adjacent replicons were converging (Fig 3A).

Relative to uninduced *SLD2*-AID, uninduced *SLD3*-AID cells had a ~5-fold reduction in rDNA origin firing even in the absence of auxin (Fig 3D and 3E) presumably due to a partial loss of function of AID-tagged Sld3 (S4 Fig). Depleting Sld3 reduced rDNA origin firing further and delayed the time at which the highest level of initiation was observed (Fig 3B and 3E). There was no discernable increase in termination/recombination structures suggesting that initiation events were so widely spaced that by T180, converging forks were not close enough to one another to be present on the same restriction fragment.

The reduced rDNA origin firing efficiency and persistence of passive replication structures indicate that the rDNA locus is particularly sensitive to Sld2 and Sld3 levels. Reduced rDNA origin firing leads to larger replicons and delays completion of rDNA replication. The persistence of branched rDNA structures explains the inability of Chr XII to migrate out of the wells of the CHEF gel and also explains its increased fragility.

### The effects of Sld2 or Sld3 depletion on unique genomic origins

**Fig 4:**
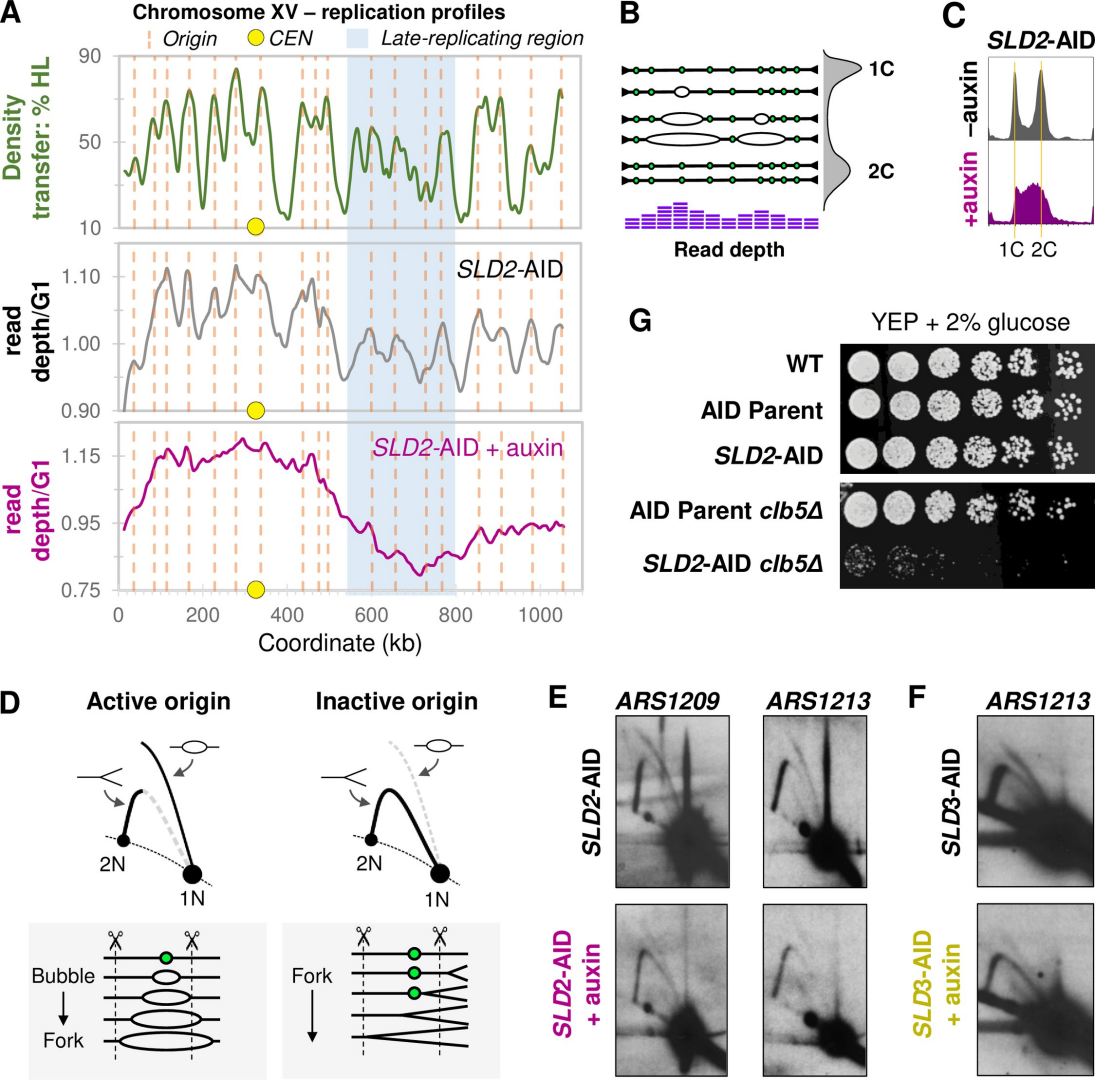
Analysis of replication at unique origins. **(**A) Whole genome sequencing (WGS)-based replication profiles for Chr XV in *SLD2-*AID. A replication profile for Chr XV based on WGS read-depth for untreated *SLD2*-AID is shown in the center panel and compared to our previously published replication data determined from a density transfer-to-microarray experiment (top panel; based on data from [82]). We define marker frequency as the read depth normalized to sequencing depth for G1-arrested cells. The vertical dotted orange lines correspond to confirmed origins in OriDB [86] that are detected in both the density transfer and the uninduced *SLD2*-AID WGS profiles. The yellow circle marks the centromere and the region highlighted in blue indicates the late-replicating domain. The bottom panel shows the WGS read-depth profile for the *SLD2*-AID strain treated with auxin for two hours. (B) Illustration of a single chromosome and how marker frequency, as measured by whole genome sequencing, reflects initiation of DNA replication based on variable origin efficiency and firing time (illustration adapted from [62]). A flow cytometry profile is shown on the right to illustrate the variable DNA content in a population of cycling cells. For haploid cells, the G1 population contains one copy of the example chromosome and the G2 population has two copies. In S phase cells, copy number along the length of the chromosome varies due to active replication bubbles. Active origins will generate local maxima in read depth, while regions where replication forks converge (termini), produce local minima. Read depth at origins that fire early in S phase will be high, while read depth at late-firing as well as inefficient origins will be lower. Large-scale chromosome regions replicated early in S phase (for example, *CEN*-proximal regions) will have higher average read depth and regions replicated late in S phase (for example, subtelomeric regions) will have lower average read depth. (C) Flow cytometry profiles for asynchronous *SLD2*-AID collected for whole genome sequencing. Orange lines indicate 1C and 2C DNA cells. The two-hour auxin treatment of *SLD2*-AID cells causes more than 50% of the cells to be distributed throughout S phase. (D) Illustration of expected 2D gel signal at an active vs. inactive origin. Arrows mark the positions of bubble and Y intermediates. The line diagrams below the 2D gel diagrams illustrate how replication forks move through the hypothetical restriction fragment (delimited by the dotted lines) containing an asymmetrically-located potential origin (closed green circle). At active origins, the bubble arc signal predominates and converts to large Ys as one of the forks passes the closest restriction site. However, when the origin fails to fire, the restriction fragment generates a uniform Y arc as forks from an adjacent origin pass through the fragment. (E) *ARS1209* and *ARS1213* 2D gel analysis for *SLD2-*AID. (F) *ARS1213* 2D gel analysis for *SLD3*-AID. (G) Spot test of *SLD2*-AID *clb5Δ* strain growth and viability. Cell cultures were serially diluted 1:3, spotted onto YEPD, grown at 30°C for three days and then photographed.

To get a broad assessment of the genome-wide consequences of depleting Sld2 or Sld3 we performed whole genome sequencing (WGS) of asynchronous cells to generate marker frequency plots (Figs 4A and 4B and S5; [62]). Since *SLD3*-AID cells failed to proceed past early S phase after 2 hours in auxin, we limited our analysis to the *SLD2*-AID strain, which is enriched for cells in S phase (~50% of cells are in S phase; Fig 4C) and compared the resulting replication profiles with those from the non-induced culture (Fig 4A).

In the absence of auxin, we found clear peaks in copy number amplitude closely paralleling the results from our previous studies using dense-isotope transfer and microarray analysis of synchronized wild-type cells (Fig 4A). Chromosome XV illustrates the distinction between early- and late- firing origins as it contains an internal late-replicating region that we previously showed to be due to a cluster of late-firing origins (blue shaded region in Fig 4A) [6, 10]. In the presence of auxin, *SLD2*-AID maintains both the early- and late-replicating domains but in both domains, there is similar decrease in the distinction between origin peaks and intervening termination regions (Fig 4A). We reason that the loss in definition between high and low marker frequency reflects reduced initiation genome-wide. To illustrate our reasoning, if the number of active origins per cell is reduced by initiation factor depletion such that different sets of origins fire in each cell, then each origin fires in a subset of the cells and is passively replicated by incoming forks in the other cells. As a result, replication fork termination will occur at different places along the chromosome across the population of cells (S5A and S5B Fig). In terms of the WGS replication profiles, this “randomization” of termination would result in the loss of distinction between peaks and valleys in marker frequency plots. However, sequencing read depth maxima will still be discernable at loci where some degree of origin activity is retained.

The loss of distinction between origin peaks and termination valleys occurs uniformly in both early- and late-replicating regions across the genome (S5C Fig). Furthermore, we observed that the range of normalized marker frequency values was considerably greater for the Sld2-depleted cells than for the control sample, which we attribute to the enrichment of cells in S phase upon depletion of Sld2 (Fig 4B and 4C). The increase in marker frequency at early-replicating regions roughly equaled the overall reduction in marker frequency at late-replicating regions (compare minimum and maximum marker frequencies across the two conditions for *SLD2-AID* in Fig 4A). Together, these observations support the idea that all origin firing is compromised by depletion of Sld2. Given that activation of the S phase checkpoint prevents activation of late-firing origins [63], we cannot say to what extent the effects on efficiency that we detect at late-firing origins is due to activation of the checkpoint.

We confirmed the reduction in origin firing for the unique origins *ARS1209*, *ARS1213* and *ARS522* by 2D gel electrophoresis (Figs 4D–4F and S6). We isolated genomic DNA from asynchronously growing cells that we treated with auxin for two hours (S6A Fig). We found a reduction in replication bubble signal and an increased Y signal intensity for the two early origins (*ARS1209*, *ARS1213*) in the *SLD2*-AID strain exposed to auxin (Fig 4E). *ARS1213* efficiency was also lower following Sld3 depletion (Fig 4F), although the *ARS1209* gel was less conclusive (S6B Fig). For late-firing *ARS522* we were unable to detect replication bubbles after depletion of either Sld2 or Sld3 (S6C Fig). While it is possible that reduced efficiency resulting from initiation factor depletion suppressed initiation more for late-firing origins than for early-firing origins, our finding that rDNA origin activation occurs at low levels for hours as cells transit S phase in the presence of auxin indicates that Sld2 and Sld3 are still functioning late in S phase in spite of their lowered concentrations.

For an independent test of whether late origin activation occurs when Sld2 is depleted, we attempted to delete *CLB5*, which is needed for origin firing in late S phase [21]. If the effects of *CLB5* deletion on growth rate, cell cycle progression, or viability were to worsen the effects of Sld2 depletion alone, we could conclude that origins are still able to fire in late S phase when Sld2 is depleted. Deletion of *CLB5* in the AID parent strain had a negligible impact on growth (Fig 4G). However, our attempts to delete *CLB5* in *SLD2*-AID, both by gene replacement using standard lithium acetate transformation and by crossing *SLD2*-AID into the AID parent *clb5Δ* strain, were of limited success. We isolated and confirmed a single, sick *SLD2*-AID *clb5Δ* clone. This *SLD2*-AID *clb5Δ* isolate could not be grown to high enough culture density for any experiments besides a spot-test, which confirmed its growth defect (Fig 4G). The *SLD2*-AID *clb5Δ* clone was inviable when exposed to 200 mM HU (S7 Fig). These results indicate that *SLD2*-AID cells are not merely relying on initiation from the early origins, because the cells still require *CLB5* to complete genome replication.

**Fig 5:**
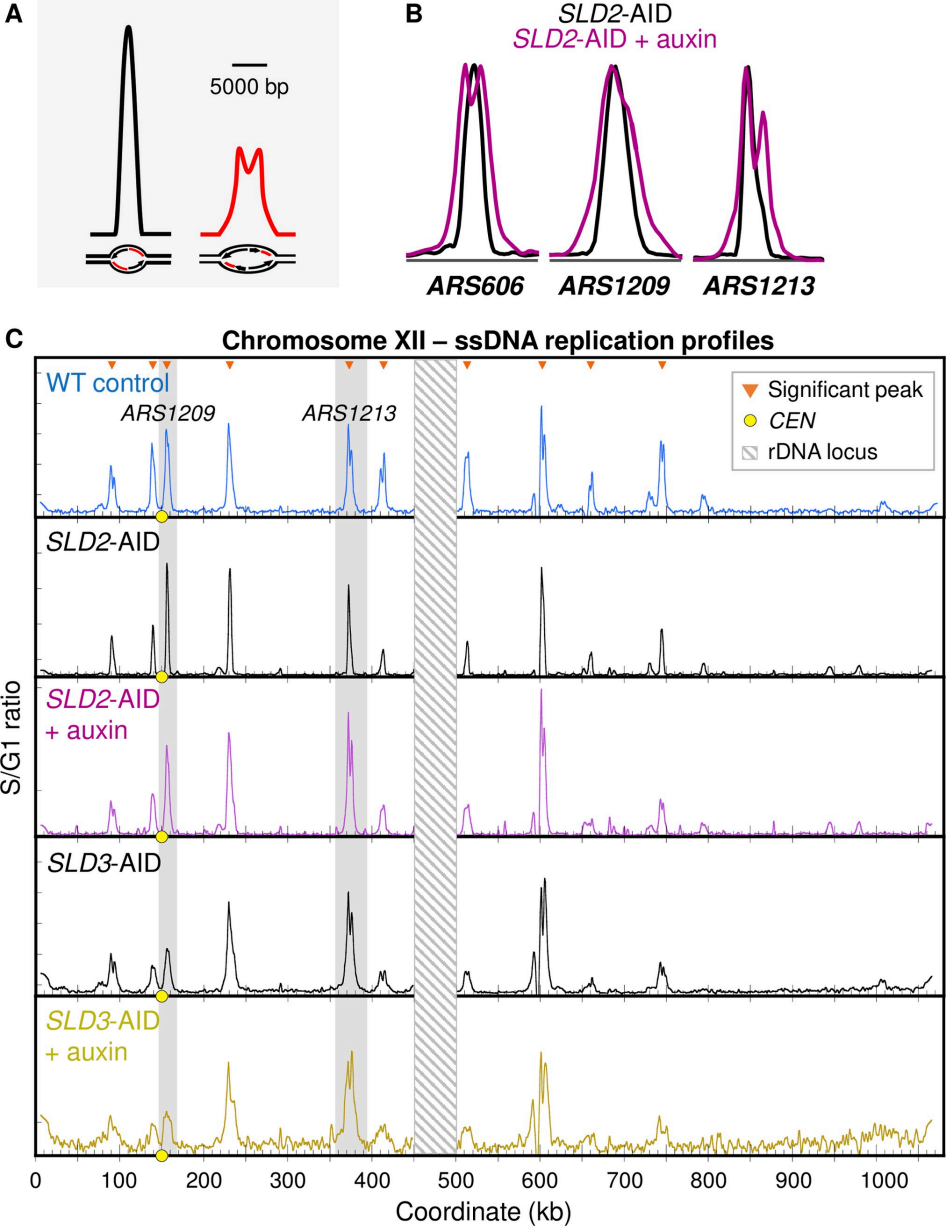
Replication profiles measuring relative origin efficiencies upon Sld2 or Sld3 depletion. (A) Cartoon illustrating the inverse relationship between origin efficiency and peak width at half maximum peak height [87]. The black peak is an example of a WT ssDNA origin peak with its corresponding replication bubble and fluorescent-labelled ssDNA (red) illustrated below the peak. The red peak is at the same origin, but when origin efficiency genome-wide is reduced. Depending on the synchrony of any particular early origin in the S phase samples, the broader peaks may or may not be “split” into two smaller peaks. (B) Examples of ssDNA peak width changes for the two *SLD2*-AID conditions at three origins. (C) Chr XII replication profiles for WT, *SLD2*-AID, and *SLD3*-AID. Profiles are generated by measuring the ratio of ssDNA labeled in S phase (60 min) relative to ssDNA labeled in G1. Orange triangles mark peaks called as significant (see Materials and Methods). Yellow dots on the X axis mark the centromere. Values at the rDNA locus (striped box at coordinates 450–500 kb) are excluded because of low probe density on the microarrays. Two origins, *ARS1209* and *ARS1213*, that we previously analyzed by 2D gel electrophoresis (Fig 4D), are highlighted in gray.

The loss of amplitude differences between origins and termination regions in the WGS marker frequency profiles (Fig 4A) and the extended time to complete individual chromosome replication (Fig 2B) strongly suggest that overall origin firing efficiency is reduced or that staggered origin firing has been extended when either Sld2 or Sld3 is depleted. In either case, individual cells in the culture would fire fewer origins at any given time and therefore have fewer active replication forks. Consequently, those fewer active forks would need to travel farther to ensure complete chromosome replication and the time required to duplicate the genome would increase. To test this prediction, we analyzed replication in the presence of hydroxyurea (HU)—a drug that inhibits ribonucleotide reductase. Cells entering S phase in the presence of HU exhaust their pre-S phase stores of nucleotides as they synthesize DNA from the earliest firing origins. If Sld2 or Sld3 depletion reduces the number of active origins per cell, then each active fork should be able to traverse a greater distance before running out of dNTPs [64]. We are able to measure how far these forks travel by mapping the positions of the ssDNA associated with active replication forks via *in vitro* labeling of nascent single-stranded regions with fluorescent nucleotides followed by hybridization to microarrays [50, 65, 66]. Active origins are revealed as peaks of ssDNA (Figs 5 and S8). If fewer forks are active in individual Sld2- or Sld3-depleted cells, then the positions of ssDNAs should be further from the initiating origins than in non-depleted cultures undergoing a similar extent of replication (Fig 5A). Note that this technique maps stable single-stranded regions (marked in red in the replication bubbles in Fig 5A) and not regions of DNA unwinding, which would revert to being double-stranded upon deproteinization during sample preparation.

We performed the ssDNA assay on WT cells as well as the uninduced and auxin-induced *SLD2-AID* and *SLD3-*AID cells. We first confirmed that the uninduced degron strains initiated DNA replication from the same origins as WT cells (Figs 5C and S8) and then examined the ssDNA profiles from Sld2- and Sld3-depleted conditions. Visual examination of the *SLD2-*AID ssDNA profiles suggested that the same set of early-firing origins is active even when Sld2 has been depleted (Figs 5C and S8). However, we noticed increased and earlier splitting of ssDNA peaks upon auxin treatment of *SLD2*-AID cells and treated as well as untreated *SLD3-*AID cells, as expected if origin firing is reduced overall and each active fork is able to move further using the available dNTPs.

**Fig 6:**
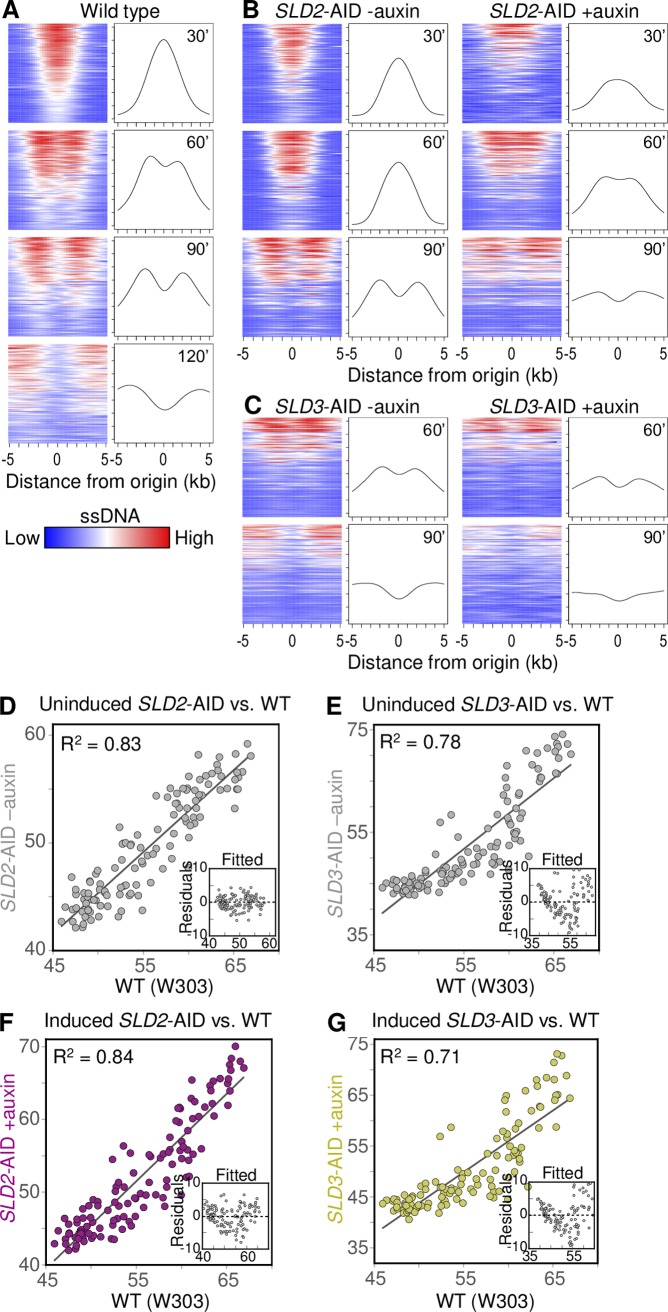
Changes to replication fork dynamics indicate reduced origin firing efficiency. (A) Aggregate views of origin activity and fork progression in wild type (W303) cells. Heat maps (left panels) show ssDNA values in 10 kb windows with 0.25 kb step size, centered at the 117 origins detected in the 30’ sample (blue = lowest, red = highest ssDNA value). Within each panel, origins (rows) are ordered in descending order of area under the ssDNA curve (i.e., sum of ssDNA values for that 10 kb window). To facilitate comparison between samples, all ssDNA values for the genome were scaled to be between 1.0 and 2.0 (see Materials and Methods). Right panels: mean ssDNA values across origins at each coordinate within the 10 kb windows (i.e., of each column in heat map). (B,C) Heat maps and mean ssDNA values for the same set of origins in the same order as in (A) for *SLD2-*AID +/- auxin (B) and *SLD3*-AID +/- auxin (C). For *SLD3-*AID, ssDNA signal was too low at 30 min to identify peaks reliably. (D, E) ssDNA peak area correlation for uninduced *SLD2-*AID and *SLD3-*AID 60 min samples, respectively, compared to wild type 60 min. Insets show residuals as a function of fitted values from the linear fit models applied to the scatter plots. (F, G) Similar comparisons with induced *SLD2-*AID and *SLD3-*AID 60 min samples, respectively.

For a more comprehensive view of the behavior of origins under these conditions, we first defined origins as those producing clearly resolved peaks in the 30 min sample of wild type (W303) cells (red circles in S8 Fig and orange triangles in, Figs 5C and S9). Under the conservative criteria we used, we defined 117 origins (Materials and Methods), comparable to the 113 origins originally defined by the ssDNA assay [66]. We based our subsequent analyses of other samples using this set of 117 origins. Fork progression leading to ssDNA peak splitting is illustrated in heat maps of ssDNA values for these origins in the wild type samples (Fig 6A left). To construct each heat map, we extracted the ssDNA values in 0.25 kb bins for 10 kb centered at each of the 117 origin locations and assigned a color value from blue (lowest ssDNA value) to red (highest value) to each bin. To establish the order in which origins are displayed (i.e., the order of the rows in the heat map), we then sorted the origins in the wild type 30 min sample by the sum of ssDNA values (i.e., the area under the curve). Origins in all other samples are sorted in this same order to reveal large-scale changes that might occur in the relative robustness of initiation across origins. To capture the overall behavior of the origins, we plotted the mean ssDNA value in each 0.25 kb bin (Fig 6A right). Together, these aggregate views of the origins clearly demonstrate the splitting and separation of peaks and hence the expansion of replication bubbles. The data also illustrate the range in origin efficiencies across the 117 origins.

Comparison of heat maps and ssDNA profiles for wild type vs. *SLD2*-AID (Figs 6B and S9) reveals several features of the *SLD2*-AID cells. First, origins in uninduced *SLD2*-AID (Fig 6B, left panels), sorted in the wild type 30’ sort order, largely retain the wild type “tornado” pattern. This result indicates that the relative efficiencies of initiation are largely unperturbed in uninduced *SLD2*-AID cells. Second, whereas undinduced *SLD2*-AID does not show evidence of peak splitting until 90’ (Fig 6B left), *SLD2*-AID cells in which degradation had been induced—taken from the same initial culture as the uninduced control—show evidence of peak splitting as early as the 30’ sample (Fig 6B right). The increased distance traveled by the forks upon induction of degradation is consistent with our conclusion above that overall origin firing is reduced upon Sld2 depletion, allowing individual forks to move further. Furthermore, the lack of fork splitting in uninduced *SLD2*-AID at early times indicates that these cells are not suffering a similar reduction in origin firing, i.e., the AID tag by itself does not detectably compromise activity of Sld2. In contrast, *SLD3-*AID cells apparently are compromised for origin activation even in the uninduced condition—ssDNA peaks in the uninduced 60’ sample were comparable to the induced *SLD2*-AID samples (Fig 6C left, S9 Fig). This result is consistent with our hypothesis that the AID-tag on Sld3 creates a hypomorphic allele (S4 Fig). Adding auxin to further reduce Sld3 function resulted in an additional increase in the distance the replication forks moved (Fig 6C right). Overall, the increased distance that forks travel when dNTP supply is limited by HU supports the conclusion that Sld2 and Sld3 depletion reduce origin efficiency across the genome.

### Sld2 or Sld3 depletion has differential effects on early origin firing

Because HU limits fork migration, passive replication of origins is no longer an issue when evaluating origin efficiency; therefore, the efficiencies of the early origins relative to each other can be assessed directly by measuring the amount of ssDNA detected at each origin (i.e., the area under each peak). We can then use the correlation between peak area under the two conditions to ask whether the relative efficiencies of different origins are maintained upon Sld2 or Sld3 depletion despite the overall reduction in origin usage.

The origin peak areas for the plus and minus auxin conditions are highly correlated (R^2^ = 0.88 and 0.97 for *SLD2* and *SLD3*, respectively; S10A and S10B Fig), initially suggesting that all origins are proportionally affected by Sld2 depletion. However, to determine whether the auxin tags were having an effect even in the absence of auxin, we compared the origin peak areas for the tagged strains to those of the parent W303 wild type (Fig 6D and 6E). The R^2^ values are slightly lower than for the same-strain comparison but are similar to those obtained when comparing two different untagged, untreated strains (R^2^ = 0.73; S10C Fig). To test the appropriateness of the linear regression, we plotted the residuals of the linear model as a function of fitted values. Random dispersion of the residuals around the horizontal (zero) would support appropriateness of a linear model. For uninduced *SLD2-*AID peak areas compared to wild type areas, the residuals from the linear fit are randomly distributed around zero (Fig 6D), indicating that the relative efficiencies of the origins being tested are preserved in uninduced *SLD2-*AID compared to wild type. These data support our conclusion above that the AID tag in the absence of auxin does not detectably compromise Sld2 function: we cannot rule out the possibility that the AID tag on Sld2 creates a hypomorph, but if so, it affects all origins more or less uniformly. In the auxin-treated *SLD2-*AID condition, peaks show a similar correlation with untreated wild type (R^2^ = 0.84; Fig 6F) with a mild skew in the residuals. These results suggest that for the *SLD2*-AID auxin treated culture the reduction in origin activity is relatively proportional across the early origins with the less efficient ones experiencing a modest disadvantage relative to the wild type control. In contrast, in *SLD3*-AID, inefficient origins were preferentially compromised in untreated as well as auxin-treated conditions (Fig 6E and 6G). In the auxin-treated condition, all but the most efficient origins are severely affected. Because of the potential for the depletion tag to affect protein activity, we subsequently based our comparisons with untagged wild type as control.

We additionally compared peak areas for two types of origins—those within 50 kb of the centromere, which are known to fire early (Natsume et al., 2013; Pohl et al., 2012), and those regulated by forkhead (*FKH1/2)* transcription factor binding near the origin [27] (panels D and E, respectively, in S10 Fig). For both origin types, the peak areas followed the same trends as the comparison of all origins detected (Fig 6D; panels D and E in S10 Fig). The high correlation among *CEN* and *FKH*-regulated origins during Sld2 or Sld3 depletion demonstrates that origins with different regulatory mechanisms experience a similar relative drop in firing efficiency.

**Fig 7:**
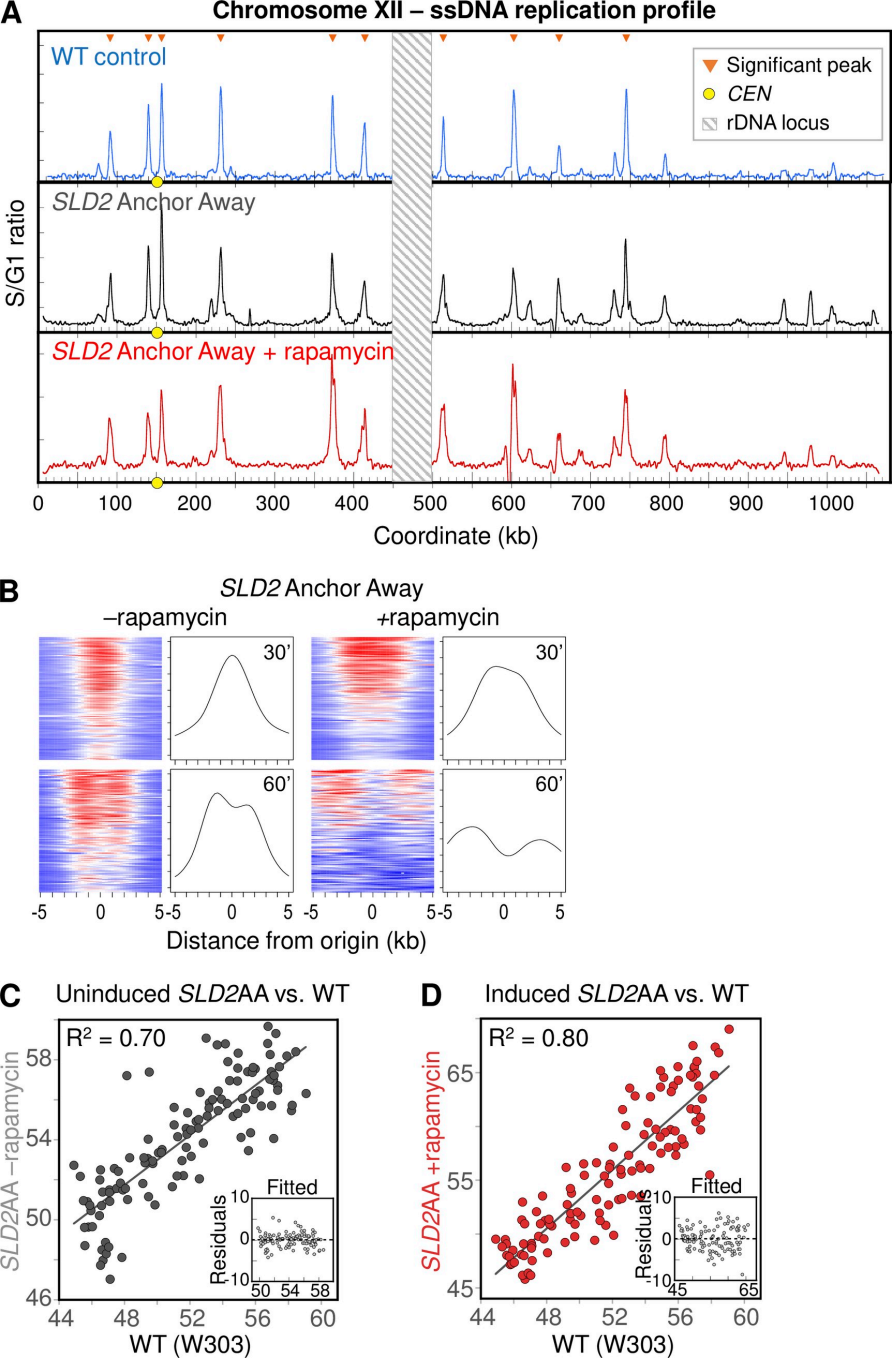
ssDNA replication profiling of *SLD2* Anchor Away cells. (A) Chr XII replication profiles for WT T30 (blue), uninduced *SLD2* Anchor Away T30 (black), and induced *SLD2* Anchor Away T30 (red). The WT profile is the same as in S8 Fig. Orange triangles mark significant peaks. Yellow dots on the X axis mark the centromere. Values at the rDNA locus (striped box at coordinates 450–500 kb) are excluded because of low probe density on the microarrays. (B) “Tornado” heat maps and mean ssDNA values showing origin activity and fork migration from the 117 significant origin peaks in control (left) and rapamycin-treated (right) in *SLD2* Anchor Away cells. See Fig 6 legend and Materials and Methods for details. (C and D) Peak area correlation for control (uninduced; C) and rapamycin-treated (induced, D) *SLD2* Anchor Away, respectively, with wild type. All samples were collected at 30 min. Insets show residuals from the linear fit model as in Fig 6.

Since the WT control and *SLD2-*AID data represent different metabolic conditions, we could not rule out either of those experimental conditions as having impacted the correlation between the two data sets. To mitigate variation due to timing and metabolic condition, we repeated the assay using Anchor Away [67], a completely different protein depletion system that does not require galactose induction, to deplete Sld2 from the nucleus. In the Anchor Away system, the target protein is not degraded, but is inducibly exported from the nucleus following the addition of the small molecule rapamycin [67]. Flow cytometry showed that addition of rapamycin had no effect on cell cycle and S phase progression of the Anchor Away parent strain (with untagged Sld2) (S11A Fig). Likewise, ssDNA profiles were indistinguishable between wild type and rapamycin-treated Anchor Away parent strain (S11B Fig) and the ssDNA peak areas were highly correlated between these two strains (S11C Fig), confirming that rapamycin on its own does not alter S phase dynamics. In contrast, flow cytometry of synchronized *SLD2* Anchor Away cells demonstrated a slowed S phase phenotype that was less pronounced than the auxin-treated *SLD2*-AID strain, as expected for reduced origin activation (S11A Fig). In agreement with this observation, tornado plots and ssDNA profiles showed clear evidence of peak splitting earlier in rapamycin-treated sample than in the untreated control (Figs 7A and 7B and S11F) with a concomitant reduction in origin efficiency (i.e., shallower tornado patterns).

When we compared the peak areas to WT, we found that for both *SLD2* Anchor Away conditions (control and depleted) the peak areas were well-correlated (R^2^ = 0.70 and 0.80, respectively, with residuals showing no evidence of skew) (Fig 7C and 7D),indicative of a proportional loss in origin efficiency among all origins upon Sld2 depletion with rapamycin (Fig 7D) as well as among *CEN-* and *FKH*-regulated origins (panels D and E in S11 Fig). The changes in origin efficiency in the *SLD2* Anchor Away strain agree with the more proportional efficiency reduction observed in the *SLD2*-AID cells.

### Overexpression of SSDD and SSDDCS produce distinct ssDNA profiles

Based on the depletion results, Sld3 appears to have a more significant role in establishing the efficiency of early firing origins than Sld2. If Sld3 were the only player, then overexpression of *SLD3* alone should result in advancement of S phase and equalization of early firing origins. However, Mantiero et al. [30] found this not to be the case. They overexpressed combinations of the limiting factors during a synchronous S phase and analyzed the replication kinetics of a handful of unique yeast sequences using a density transfer technique and an alkaline gel protocol. They found S phase to be compressed but the relative order of replication timing was preserved when only the SSDD factors were overexpressed. In addition, they found the increase in origins fired per cell resulted in a decrease in the distance individual forks traveled before exhausting dNTPs. In a separate study, McGuffee et al. [31] employed an Okazaki fragment mapping technique to examine origin/termination regions during overexpression of SSDDCS factors in an asynchronous culture. They presented evidence suggesting that origin efficiencies became more uniform. Because these two studies were carried out with three different techniques and implied different types of changes to initiation dependent upon whether Cdc45 and Sld7 were also overexpressed, we repeated the overexpression of both sets of factors and examined replication by the ssDNA assay under the same growth conditions used for the *SLD2*-AID and *SLD3*-AID strains. Since Mantiero, et al. [30] demonstrated that both SSDD and SSDDCS overexpression strains produced replication intermediates at multiple origins during S phase in HU, and since McGuffee, et al. [31], using a completely different assay, demonstrated the production of replication intermediates with SSDDCS overexpression, we were confident that the ssDNA assay would allow us to assay bona fide replication initiation.

**Fig 8:**
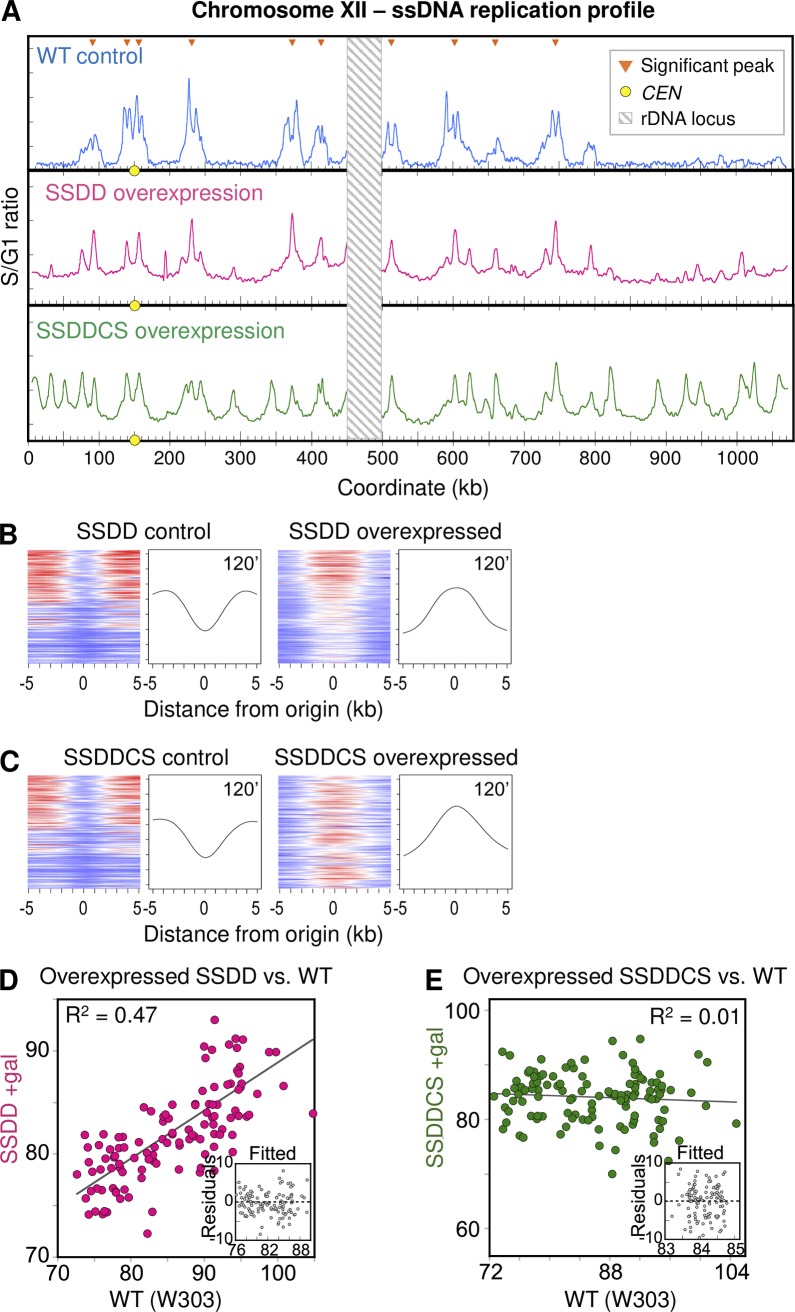
ssDNA replication profiles for SSDD and SSDDCS factor overexpression. (A) Chr XII replication profiles for WT, SSDD overexpression, and SSDDCS overexpression T120 samples. Orange triangles mark positions of significant peaks as defined for wild type at 30 min (see Materials and Methods). Yellow dots on the X axis mark the centromere. Values at the rDNA locus (striped box at coordinates 450–500 kb) are excluded because of low probe density on the microarrays. (B) “Tornado” heat maps and mean ssDNA values showing origin activity and fork migration from the 117 significant origin peaks in control (left) and SSDD overexpression (right). (C) Similar plots for SSDDCS. See Fig 6 legend and Materials and Methods for details. (D and E) Peak area correlation for SSDD overexpression (D) and SSDDCS overexpression (E) with wild type. All samples were collected at 120 min. Insets show residuals from the linear fit model as in Fig 6.

Our flow cytometry analysis of SSDD and SSDDCS overexpressing cells suggested that SSDDCS overexpression allows cells to enter and complete S phase faster than SSDD overexpression. These results suggest that there are different replication initiation dynamics between the two conditions (S12A Fig). During SSDD overexpression, we detected peaks at the early-firing origins that are detected in the WT condition as well as some small peaks at later-firing origins (Figs 8A and S12B). However, the observed peaks had variable areas, indicating that differences in origin efficiency persist even upon SSDD overexpression (Fig 8A). Considering the origins in aggregate, we see an opposite effect in the tornado plots to what we observed with Sld2 or Sld3 depletion: splitting of peaks is now delayed compared to the control condition, consistent with more initiation occurring per cell (Fig 8B). However, the tornado pattern does not reach the bottom of the plot, indicating again that efficiency differences persist between origins even upon SSDD overexpression. Overexpression of SSDDCS also delayed splitting of peaks (Figs 8A and 8C and S12B). In contrast to SSDD overexpression, SSDDCS overexpression resulted in additional active origins in regions that are usually late-replicating as well as more uniform peak areas among all origins (0–80 kb, 250–350 kb, 800–1000 kb regions in Fig 8A). These changes are reflected as a column of red color (i.e., robust peaks) extending all the way down the tornado plot (Fig 8C). There is still a positive correlation with the WT ssDNA peak areas for SSDD overexpression (R^2^ = 0.47; Fig 8D) suggesting that whichever factor is responsible for distinguishing efficiency among origins was still largely in limiting supply. The general uniformity of peaks in SSDDCS-overexpressing cells translated to a loss in correlation with WT peak area (R^2^ = 0.01; Fig 8E) and indicates that differences in origin efficiencies were largely eliminated. These observation support the conclusion that the number of origins fired per cell is lowest in wild type and highest in SSDDCS overexpression cells. Overall, these data are consistent with either Sld7 or Cdc45 (or their combination) setting the intrinsic efficiency of origins across the yeast genome.

## Discussion

Inspired by the model proposed by Mantiero et al. [30] to explain their observation that simultaneously increasing the cellular abundance of limiting initiation factors advances origin firing time and increases origin efficiency [30], we wanted to ask: How do these factors contribute to the regulation of replication origins? Would further restricting the availability of these factors have disproportionate consequences for specific origins or specific regions of the genome? What are the individual roles of these factors in determining firing time or efficiency? We have approached these questions both by reducing single limiting factors and by overexpressing combinations of the limiting factors. We then analyzed genome replication using whole-genome as well as locus-specific techniques. In particular, we sought to determine whether altering the abundance of limiting factors changes the intrinsic firing efficiency of origins or alters their ability to initiate replication in the presence of hydroxyurea (HU)—a property that has become synonymous with “early” firing status.

We find that when Sld2 abundance is reduced the distinction between origins and termination regions in our marker frequency analysis is dampened. These results suggest that individual cells use fewer origins to complete genome replication but that, in the population as a whole, the same relative efficiency and timing preferences remain. This marker frequency profile is strikingly similar to the megabasepair-sized “timing domains” observed in replicating metazoan genomes [9, 68, 69]. In metazoans, it is thought that these timing domains contain many adjacent, low efficiency origins that tend to fire within the same span of time during S phase [9, 69].

Results from the ssDNA assay, in which we measure the accumulation of ssDNA associated with replication forks generated from the early-firing origins—estimated to comprise ~35–55% of all origins [65, 70]—are consistent with the interpretation that individual cells in the population fire fewer origins, with different cells firing different subsets of origins, leading to an overall reduction in origin efficiency. We find that individual replication forks travel further from each active origin presumably because there are fewer active replisomes per cell competing for limiting dNTPs [64]. However, even with reduced origin firing per cell, due to the presence of HU the forks do not travel far enough to passively replicate through adjacent origins, allowing us to measure relative intrinsic origins efficiencies without the complication of passive replication. Because we see only a relatively weak effect on origin activity upon Sld2 depletion, we conclude that Sld2 is not the major player in establishing the differential efficiencies or firing times of early origins in yeast.

Compromising Sld3 activity, on the other hand, has a more noticeable differential effect on origin activity. One difference we noted between *SLD2*-AID and *SLD3*-AID is that the presence of the auxin degron on Sld3 reduces its function even in the absence of auxin. For example, the growth rate, the number of rDNA repeats, and the distance that forks move in the ssDNA assay are altered relative to WT, suggesting that *SLD3*-AID is a hypomorphic allele. The degron domain on Sld3 may interfere with the protein’s stability or its interactions with other members of the assembling replisome, such as Sld7, Cdc45, or the cyclin-dependent kinase. This hypomorphism coupled with the fact that Sld3 is already less abundant than Sld2 in WT cells [30, 45] may explain why the phenotypes we observed were often more severe for Sld3 depletion than for Sld2 depletion.

The Sld3 results raised the possibility that Sld3 availability could explain the differences in efficiency between origins. To expand this analysis, we attempted to deplete other limiting factors. We were particularly interested in Cdc45 since it is the only replication initiation factor that also travels with the fork, a property that suggests recycling of Cdc45 for new rounds of helicase activation would be inherently restricted. As we were not able to recover AID-tagged Cdc45, Dpb11 or Dbf4, we turned to overexpression studies. Previous work had explored the consequences of overexpressing different sets of the limiting factors, using different types of assays to characterize their phenotypes. Single locus methods (alkaline gels and density transfer experiments) suggested that a few late regions were advanced under normal growth conditions or were now able to replicate in the presence of HU [30, 32]. Those papers examined different loci and expressed different sets of limiting factors. Whole genome analyses—Okazaki mapping [31] and SOLiD whole genome sequencing of HU treated cells [32]—suggested that late origins are being activated earlier than in non-overexpressing cells. However, because they used different subsets of overexpressed limiting factors, it is difficult to completely reconcile their results into a single model. To resolve some of the confusion, we felt it worthwhile to repeat some of these overexpression studies, using the same ssDNA assay that we used on the depletion strains. In particular, we predicted that if Sld3 was the primary determinant of origin efficiency, then SSDD overexpression should eliminate efficiency differences between origins.

The ssDNA profiles for SSDD-overexpressing cells indicate that while some late origins fire in HU, their peak areas are lower than for the early-firing origins. In addition, the comparison of early origin efficiency between WT cells and SSDD-overexpressing cells reveals the persistence of a positive correlation, suggesting that differences in efficiency among origins are still maintained. These results are in agreement with those of Tanaka et al. [32] in that the additional peaks we detect overlap in large part with the minor peaks they found in their HU-sequencing experiments. However, the ssDNA profiles for SSDDCS-overexpressing cells show robust initiation at essentially all origins in the genome. This loss of initiation preference is also reflected by the profound reduction in peak area correlation between WT and SSDDCS-overexpressing cells (R^2^ ~0.01). The equalization of origin efficiency that we observed in SSDDCS cells but not in SSDD cells is also supported by experiments on two origins reported by Mantiero et al [30]. Using alkaline gel analysis to detect replication intermediates at an early (*ARS305*) and a late origin (*ARS501/522)* in HU, Mantiero et al. [30] identified replication intermediates at both loci for both overexpression conditions (see S10 Fig of that publication). However, the difference in hybridization intensity is preserved between the two origins in the SSDD overexpression condition while the intensity is essentially the same at the two origins when all six limiting factors are overexpressed.

Mantiero et al [30] also found that SSDDCS-overexpressing cells activate the S phase checkpoint via Rad53 phosphorylation in response to limiting dNTPs. The fact that we observe initiation at essentially all origins in HU suggests that the origins fire before the Rad53-inhibition of late origin firing can take place. Likewise, Mantiero et al. [30] demonstrated that Rad53 is phosphorylated in the SSDD overexpression strain during S phase in HU. The failure to see robust activation of late-firing origins (Fig 8) indicates that it is the activation of the Rad53 checkpoint that is inhibiting their initiation.

The loss of initiation preference we observe during SSDDCS overexpression is in agreement with the results of McGuffee et al. [31], who found that fork termination sites were tightly clustered at the midpoints between adjacent origins upon SSDDCS overexpression. Furthermore, consistent with the alkaline gel results of Mantiero et al. [30] and Tanaka et al. [32], we observe relatively low levels of initiation at *ARS501* and *ARS603* with SSDD overexpression, but high levels with SSDDCS overexpression (S12B Fig, coordinates ~549 kb on chr V and ~69 kb on chr VI, respectively). From our results, we conclude that it is primarily the levels of either Cdc45 or Sld7, or both, that are responsible for determining genome-wide differences in origin efficiency although their contribution to origin efficiency is only uncovered when Sld2, Sld3, Dbf4, and Dpb11 are no longer limiting. This conclusion is consistent with the observation by Belsky et al. [71], who found by chromatin footprinting that although all origins showed an ORC footprint in G1, only a subset of origins had an ORC-dependent footprint in G2 while origins that are likely to be inefficient lacked the G2 footprint. They suggested that delayed assembly of preRCs at the latter class of origins might impede recruitment of limiting factors such as Cdc45 or Dbf4. Equally intriguing is the report by Azmi et al. [72] that in their in vitro replication assays, chromatin remodeling by SWI/SNF and RSC permitted origin licensing but inhibited origin activation by interfering with CMG assembly. Our observation that origin efficiency is equalized by SSDDCS overexpression—but not by SSDD overexpression—raises the possibility that the increased dosage of Cdc45 and/or Sld7 could be overcoming chromatin effects of remodelers such as SWI/SNF or RSC to allow robust CMG assembly at all origins. The fact that Cdc45 is an integral part of the active helicase and thus moves with the forks while all of the other five factors are required for helicase assembly and/or activation, and the fact that Sld7 is not essential [37], make Cdc45 the strongest candidate. Work in human cells indicates that Cdc45 is limiting for replication initiation and that overexpression of Cdc45 alone leads to S phase arrest, accumulation of ssDNA and apoptosis [73].

Overexpression of SSDD limiting replication factors during early *Xenopus* development has been shown to delay the mid-blastula transition and to interfere with normal development [38]. Phenotypes associated with SSDD overexpression in yeast include reduced S phase length, activation of the Rad53 checkpoint, and, on continuous culture, to severe growth defects [30]. In neither of these cases, nor in the case of Cdc45 overexpression in human cells, is it clear how changes to the replication initiation program cause lethality. In our Sld2 and Sld3 depletion experiments, we also see significant effects on growth rate, S phase progression, and viability. We see a slight increase in Rad53 phosphorylation, which may contribute to a reduction in firing efficiency of origins that fire later in S phase. We find that the most notable consequence of reduced origin firing is increased instability of the rDNA locus.

When we induce depletion of either Sld2 or Sld3, Chr XII is unable to migrate at its normal position in CHEF gels suggesting that replication is incomplete; the branched DNA structures that prevent migration in the CHEF gel appear to be confined to the rDNA array. Incomplete replication eventually leads to Chr XII fragmentation and, in the strain with the hypomorphic *SLD3*-AID allele, seems to provide selective pressure for reduced rDNA array size from ~250 copies of the rDNA locus to ~90 copies. During Sld2 and Sld3 depletion, we find rDNA origin efficiency is reduced more than tenfold while the ssDNA peak areas indicate that genomic origins are reduced by approximately twofold.

Several structural features of the rDNA locus make replication especially challenging: First, the origins in each repeat are inherently inefficient due to a single nucleotide variant in the origin consensus sequence [56–58]. Even in wild type cells, on average, only one in approximately five origins fires in a given S phase [7]. This inherent inefficiency leads to an average spacing of ~45 kb between active origins; however, the actual distance is likely greater as active origins appear to be clustered [61]. Second, Fob1 [74] binds to a site at the 3’ end of the 35S transcription unit (the replication fork barrier; RFB; [7]) and blocks forks from encountering RNA polymerase 1 complexes oriented opposite to the replication complex. Each initiation event in the rDNA effectively produces a single active fork that must cover the span between adjacent initiation events. As a consequence of this unidirectional replication, resolution of replication gaps in the rDNA could take longer than for replication gaps in the unique portions of the genome. Third, the rDNA origin is more sensitive to Sld2 or Sld3 depletion. Unlike unique regions of the genome where origins of differing efficiencies and timing are interspersed, each 9 kb repeat in the ~2 MB rDNA locus has the same inefficient origin. Therefore, the greater reduction in origin firing when Sld2 or Sld3 is limited generates very large initiation-free regions. While random replication gaps may appear in the unique portions of the genome, they are more likely to be filled by forks from nearby origins that do manage to initiate. The specific replication gaps in the rDNA only have other rDNA origins to rely on for completion of replication. While this study is not the first to show a link between initiation defects and rDNA instability [75–78], our study provides a mechanistic explanation for the importance of interspersed origins of different efficiencies/timing for overall maintenance of genome integrity.

The rDNA instability in *S*. *cerevisiae* suggests that large spans of repeated DNA elements may likewise be a problem for genomes with much more repetitive content, such as the human genome. For example, the rDNA loci in humans, while divided into arrays on 6 different chromosomes, have a tandem repeat structure [79]. Human centromeres, which are megabasepairs long, also consist of tandem repeats primarily made up of α satellite DNA [80, 81]. The yeast rDNA locus is the only part of the yeast genome that approximates the structure of these long, tandemly-repeated parts of the human genome, and its sensitivity to replication initiation defects suggests that defective replication initiation may also impact the stability of similarly repetitive regions of metazoan genomes. Generally, it would be challenging to determine whether large repeated DNAs in metazoan cells are similarly sensitive to replication initiation defects because the requirements for origins are still unclear in those organisms (reviewed comprehensively in [2]). Nevertheless, our work suggests that DNA replication through repetitive sequence could be a potential challenge for metazoans in terms of ensuring complete replication and maintaining genome stability.

## Materials and methods

### Strain construction

The parental strain for this study is W303 *rad5*Δ *MATa*, which is used as a wild-type (WT) control throughout. We used standard yeast lithium acetate transformation for all transformations. In the auxin inducible degron (AID) strains, the *GAL1-10*-*Os*TIR1-9myc construct is integrated at *URA3*. We C-terminally tagged Sld2 and Sld3 with 3xmini-AID [43] by transformation and confirmed integration of the AID tag by PCR and Southern blot hybridization. Takashi Kubota and Anne Donaldson at the University of Aberdeen generously donated the auxin degron construct plasmids to us. We confirmed Sld2 and Sld3 degradation following auxin induction by flow cytometry for delayed or slowed S phase. We constructed the *RAD53*-2xHA tagged versions of the AID strains by integrating linearized plasmid containing a partial copy of *RAD53* with the HA tag. The Bedalov lab at the Fred Hutchinson Cancer Research Center donated the *RAD53*-2xHA plasmids. The Anchor Away FRB domain [67] was integrated at the C terminus of Sld2 in the Anchor Away parent strain as above. The Henikoff lab at the Fred Hutchinson Cancer Research Center donated the Anchor Away parent strain and pFA6a-FRB-KanMX6 plasmid. See S1 Table for oligos and S2 Table for list of strains and genotypes.

### AID strain induction

All liquid culture was in yeast synthetic (YC) medium. YC medium contains per 1000 ml: 1.45 g yeast nitrogen base without amino acids and ammonium sulfate (Becton-Dickinson); 10 g succinic acid; 6 g dry NaOH; 5 g ammonium sulfate; 1.4 g dropout mixture; supplemented with a carbon source of choice. The dropout mixture is made by mixing the following: 2 g each of dry adenine, arginine, histidine, methionine, and uracil; 5 g phenylalanine; 6 g each of lysine and tyrosine; 8 g each of leucine, isoleucine, and tryptophan; 10 g each of aspartic acid and glutamic acid; 15 g valine; 20 g threonine; and 40 g serine. For selective growth, the appropriate component is left out of the dropout mixture and the amount of the mixture added is scaled accordingly. For asynchronous experiments we grew cells in YC + 1% raffinose (YCR) medium until early log phase and induced *Os*TIR1-9myc expression with 2% galactose for 3 hours before adding 0.5 mM auxin (IAA) for a minimum of 2 hours. Unless specified, negative controls are the degron strains without auxin (see Fig 1B). As needed, we diluted cultures during galactose induction to maintain log phase growth. For synchronous S phase experiments, we grew cultures in YCR to early log phase, then synchronized them with 3 μM α-factor for 3.5 hours. Thirty minutes after adding α-factor, we added 2% galactose and, one hour before release, we split the cultures and added auxin to one half at a final concentration of 0.5 mM. We collected flow cytometry samples for every experiment to confirm initiation factor degradation. Growth data are in S11 Data.

### Anchor Away strain induction

Cells were grown in liquid YC + 2% dextrose. To synchronize the cells, we grew cultures to early log phase, then added 3 μM α-factor for 3.25 hours total. Thirty-five minutes before release into S phase, we split the G1-arrested culture into two portions and added 1 μg/mL rapamycin to one portion to induce nuclear depletion of the target protein.

### Flow cytometry

We collected one mL of culture per sample and prepared samples as in Alvino, et al [82] using SYTOX Green Dead Cell Stain (ThermoFisher). We assayed 10,000 events per sample on a BD FACSCanto cytometer and analyzed the data in FlowJo. For the degron strains, we gated all samples based on the uninduced asynchronous culture.

### Growth and viability

To determine viability, we plated 300 cells in triplicate on YEP + dextrose (2%) and counted colonies after 2 days of growth. YEP contains 10 g yeast extract and 20 g bacto peptone per 1000 ml. For spot-testing, we suspended the cells in water, sonicated, then serially diluted the cells in a 1:3 ratio and spotted 5 μl onto appropriate plates. We grew the cells at 30°C and photographed the plates each day. Viability values are in S11 Data.

### Western blotting

For each sample, we collected ~4.0 x 10^7^ cells after G1 synchronization and galactose induction as above. The positive control samples were treated with 200 mM hydroxyurea for 10 minutes before release from G1. We extracted protein by bead beating in SUMEB pH 6.8 (1% SDS, 8 M urea, 10 mM MOPS pH 6.8, 10 mM EDTA, and 0.01% bromophenol blue) plus ThermoFisher protease inhibitor cocktail and 5% 2-Mercaptoethanol. We probed with 1:2500 α-HA (BioLegend/Covance MMS-101R) and 1:10,000 α-PGK1 (Abcam #ab197960) as a loading control.

### CHEF gel analysis of chromosome stability

We used contour-clamped homogeneous electric field (CHEF) gel electrophoresis to assay chromosome integrity and stability during synchronous S phase [51, 52]. We collected 5 mL of culture for each timed sample then embedded the cells in agarose. We ran the samples on a 1% agarose gel on Bio-Rad CHEF electrophoresis apparatus at 100 V for 68 hours, switch time ramped from 300 to 900 seconds as in Kwan et al. [83]. We transferred the gels using standard Southern blotting methods to GeneScreen nylon membrane (PerkinElmer) and probed the same blots multiple times for single copy sequences on different chromosomes, stripping between each hybridization. We used a Bio-Rad Personal Molecular Imager and QuantityOne software to quantify hybridization signal. CHEF gel quantification values are in S11 Data.

For the *in gelo* I-PpoI restriction digest, we washed the plugs 3x in 10 mM Tris (pH 8), then pipetted 75 μl I-Ppo1 buffer plus 0.5 μl I-Ppo1 onto a small slice of each plug and incubated for 1 hour at 37°C. We ran each sample using the same CHEF gel parameters as above.

### WGS replication profiling

For each sample, we collected 50 mL mid-log phase (OD_660_ ≈ 0.5) cells as well as a G1-arrested control sample after 120 minutes in α-factor. We purified genomic DNA by the standard Smash & Grab protocol [84]. We sheared the DNA to an average length of 260 bp using a Covaris ultrasonicator, then treated the sample with 0.01 mg/ml RNAse A before a final clean up using the Zymo Research DNA Clean and Concentrator-25 kit. We end-repaired and adapter-ligated 100 ng of DNA per sample with the KAPA Biosystems Hyper Prep Kit and the KAPA Single-Indexed Adapter Kit. We sequenced on the Illumina NextSeq 500 platform. We aligned single reads of at least 25 bp to Saccer1 using Bowtie2 then binned the reads into 1 kb windows using custom Python scripts. When possible, we aimed for 10 million reads per sample before generating replication profiles.

To generate profiles, we first calculated average read depth for each sample, excluding the rDNA locus, 2-micron plasmid, and the mitochondrial DNA and normalized the read depth in each 1 kb bin to this average. We determined marker frequency for each bin by dividing the bin read depth by the corresponding bin read depth of the G1 cells. We found that poor mappability of repetitive subtelomeric DNA sequences, transposable elements, and highly conserved paralogous genes descended from the whole genome duplication event resulted in outlying marker frequency values that distorted the replication profiles when smoothing the data. Consequently, we excluded these poorly-mapped regions, which are listed in S3 and S4 Tables, from each dataset. Finally, we LOESS smoothed the marker frequency values in 50 kb windows to generate replication profiles. All sequencing reads are available at SRA accession #SRP156227. Processed data are included in Source Data files (S1 Data).

### 2D gel electrophoresis

We performed 2D gel electrophoresis analysis for the high copy rDNA origin and unique origins using different DNA purification and restriction digest protocols for each type. For each timed rDNA sample, we collected 50 ml culture volume by chilling the cells on frozen 0.1% sodium azide plus 0.025 M EDTA. We then embedded the cells in 0.5% low melt (SeaPlaque) agarose plugs and spheroplasted [50]. To digest the DNA, we cut a single 90 μl plug in half and washed it 3x in 10 mM Tris, then equilibrated 3x in 200 μl NEBuffer 2.1 on ice with gentle shaking. We removed the buffer and pipetted 3 μl NheI (10,000 units/ml; NEB) directly onto the plug and incubated it for 4 hours at 37°C. We loaded each plug onto a comb and poured the 0.5% agarose first dimension gel around the plugs. We ran the first-dimension gel for 20 hours at 1V/cm without ethidium bromide. We cut the resulting gel slice at least 1 cm below the 4.7 kb NheI fragment that contained the origin, then transferred each gel slice to a casting tray and poured the 2^nd^ dimension gel. We ran the 1.1% agarose second-dimension gel with 0.3 μg/ml ethidium bromide for 6 hours at 7 V/cm in the cold room with circulating buffer. We probed the membranes with the *NTS2* sequence and quantified the rDNA 2D gels as depicted in Fig 3C. The timed collection for rDNA 2D gels was performed once.

For single copy origins, we collected 300 mL of mid-log phase culture (OD_660_ = 0.5–0.7). We chilled the sample on frozen sodium azide and EDTA and purified genomic DNA using a variation of the “NIB (nuclear isolation buffer) & Grab” DNA purification protocol detailed on the Brewer/Raghuraman lab website [85]. Instead of using glass beads and vortexing alone to lyse the cells, we incubated the cells in 1 mg/ml zymolyase in NIB for 30 minutes at room temperature, and then used one 30s round of glass bead-beating to ensure lysis before continuing with the standard NIB & Grab protocol. We find that zymolyase-mediated cell lysis better preserves replication intermediates.

We used all of the DNA purified from 300 mL of culture (a few micrograms) for each 2D gel for each single-copy origin analyzed. We used the enzyme BanI to analyze *ARS1209* (4.8 kb fragment) and *ARS1213* (3.7 kb fragment). Due to a polymorphism that changed the BanI site in *SLD3-*AID, we used BspHI to analyze *ARS1209* (4.9 kb fragment). For *ARS501/522*, we used XbaI to cut a 3.2 kb origin fragment. We ran the 0.4% first dimension agarose gel at 1 V/cm for 18 hours without ethidium bromide. We ran the 1.1% agarose second-dimension gel with 0.3 μg/ml ethidium bromide in the cold room with buffer circulation at 7 V/cm for 4–5 hours, depending on the size of the origin fragment. After transfer, we probed the blots with a fragment containing the origin of interest. See S1 Table for the oligos used to amplify the probe fragments. At least two biological replicates (meaning DNA collected from independent samples) were performed for the asynchronous culture 2D gels. The uninduced and induced pairs of gels are shown from the same biological replicate. 2D gel quantification values are in S11 Data.

### ssDNA mapping replication assay

We grew cultures to OD_660_ = 0.25, α-factor arrested them and added auxin as described above. We grew the WT cells in YC + 2% glucose. We added hydroxyurea (final concentration = 200 mM) 10 min before release from α-factor and collected S phase samples at 30, 60, 90, and 120 minutes after release. We prepared samples using the *in gelo* ssDNA labeling protocol detailed by Feng et al. [50], and used β-agarase (NEB #M0392S) to recover the labeled ssDNA. We co-hybridized one G1 and one S phase sample to Agilent ChIP-on-chip 4x44 *S*. *cerevisiae* microarray. We processed and normalized the array data as described in Feng, et al. [65], with one additional processing step: we eliminated outliers by discarding values more than 8 standard deviations above and less than two standard deviations below the mean ssDNA value for the whole genome. We then used LOESS regression to smooth the data over a 6 kb window except as noted below. Raw intensity values and processed microarray data are available in S2–S8 Data Files. We analyzed the 60-minute samples for the AID strain because earlier timed sample profiles may have high signal-to-noise and later timed samples are impacted by loss of replication fork synchrony [65]. We analyzed the 30-minute timed sample for the Sld2 Anchor Away cells to match of the effects of Sld2 depletion with the timed wildtype control sample. The galactose-driven SSDD and SSDDCS overexpression strains were grown in 1% raffinose and induced with 2% galactose during the 2.5 hour α-factor arrest. We analyzed the 120-minute samples for these two strains and compared them to the 120-minute wild type sample because with the reduced fork migration in the overexpression strains peaks were not large enough to measure reliably until the later timed sample. For these analyses, smoothing was over a 9 kb window to compensate for the increased noise in the data.

### Defining origin peaks

We first defined origin locations based on peaks called from the 30 min wild type (W303) sample. We identified all peaks (including the small peaks in the “noise” in the baseline) based on local maxima after LOESS smoothing, i.e., locations where the slope of the profile changed from positive to negative. Then, we called significant peaks as those that were in the top 5% of all peaks. In one instance of a peak that was called on the shoulder of a larger peak that was less than 5 kb away, we excluded the smaller peak. Overall we were left with a list of 117 significant peaks, closely matching the 113 peaks called in the original ssDNA mapping paper [66]. *CEN-*proximal origins are the origins within 50 kb of the centromere on each chromosome that are detected in the WT data. *FKH*-regulated origins are origins identified as being either positively or negatively regulated by *FKH* transcription factor proteins by Knott et al. [27]. S5 Table includes the full list of origins analyzed by ssDNA mapping.

### ssDNA heat maps and origin peak areas

To construct the heat maps of ssDNA values, we rescaled the ssDNA values to fall between a baseline value of 1 and a maximum of 2 as follows to facilitate comparison between samples. We first applied LOESS smoothing to the data as described above to get smoothed data points spaced at 0.25 kb intervals. We then defined the baseline (ssDNA_*baseline*_) for each sample as the mode of the distribution of all smoothed ssDNA values for the whole genome. Finally, we rescaled the values for all coordinates by applying the transformation:

$${\text{Rescaled}\;\text{Value}}_{i}=\frac{{\text{ssDNA}}_{\text{i}}\;{\text{-}\;\text{ssDNA}}_{baseline}}{{\text{ssDNA}}_{max}\;{\text{-}\;\text{ssDNA}}_{baseline}}+1$$

where ssDNA_*max*_ is the maximal smoothed ssDNA value in the genome for that sample. From these rescaled data sets, we extracted the ssDNA values in 10 kb windows centered at the list of 117 origins described above. We then sorted these windows in order of the sum of the values in each window (i.e., the area under the curve) for the wild type 30 min sample and used the ‘heatmap3’ library in the software package R to generate heat maps of ssDNA values. All subsequent samples were sorted in this wild type 30 min order. The color range was set to be the same across all samples (blue = lowest value to red = highest value). We used these values of peak areas for the scatter plots comparing origin efficiencies. For the SSDD and SSDDCS peak area comparisons, because of the broader peaks in control conditions, we expanded the window size to 16 kb centered on peak locations. Peak areas and heat map values are available in S9 and S10 Datas, respectively.

### Data availability statement

All sequencing reads are available at SRA accession #SRP156227. Processed data are included in Source Data Files.

### Key resources used

See S6 Table.

## Supporting information

S1 Fig AID parent strain cell cycle progression and growth. (A) We tested cell cycle progression in the AID parent strain to determine whether any off-target auxin-induced protein degradation changed S phase progression. We have included induced and uninduced *SLD2-AID* and *SLD3-*AID strains (Fig 2) for comparison. The thin orange line marks the position of cells with 1C DNA and the thick vertical black bars indicate the approximate duration of S phase in each condition. (B) AID parent strain growth. The auxin-treated cells had a slightly slower growth rate (17% increase) but auxin did not impact viability. (C) Flow cytometry profiles for the AID strains for each condition used for western blotting of Rad53 phosphorylation in a *RAD53-*2xHA strain background. Bold, red text indicates the samples analyzed by western blot. (D) Rad53 phosphorylation in the AID parent strain.
(PDF)

S2 Fig AID parent strain CHEF gel analysis and additional chromosome CHEF gel blots. (A) CHEF gel quantification to determine the percentage of signal in the well of each lane. The hybridization signal from a section above the well was subtracted from the well signal and the lane signal portion of each sample to correct for background. The corrected well hybridization was then divided by the total hybridization signal for each individual sample to determine the “percent well signal” shown in the bar charts in (B)–(I). (B) Quantification of Chr IV *SLD2*-AID and *SLD3*-AID blots. The gray shading highlights the regions of maximum discrepancy between the uninduced and induced samples. (C) Chr IV-probed CHEF gel blots and quantification for the AID parent strain. (D) Quantification of *SLD2*-AID and *SLD3*-AID Chr XII CHEF gel blots. The gray shading highlights the regions of maximum discrepancy between the uninduced and induced condition. (E) Chr XII-probed blots and quantification for the AID parent strain. (F) Chr XV-probed blots and blot quantification the AID strains. The probe is a single copy sequence at ~810 kb. (G) Chr X-probed (*ARS1011*) blots and quantification. (H) Chr III-probed (*ARS306*) blots and quantification. (I) Single copy mitochondrial DNA sequence (*COX1*) probed blots and quantification. Flow cytometry profiles for the AID parent control are shown in S1 Fig.
(PDF)

S3 Fig Flow cytometry profiles for asynchronous *SLD2-*AID for CHEF gel analysis. Profiles for asynchronous *SLD2-*AID cells treated with auxin for 120 minutes, 240 minutes, and grown to saturation overnight.
(PDF)

S4 Fig The C-terminal AID domain on Sld3 causes partial loss of function. To test for compromised Sld3 function, we constructed a version of the *SLD3*-AID strain without the E3 ubiquitin ligase. (A) Flow cytometry profiles for asynchronous growth in WT, AID parent, *SLD3*-AID, and two clones containing degron-tagged Sld3 but no *GAL1-10*-*Os*TIR1 construct. The G1 peaks are height-matched, and the dashed line indicates the proportion of S phase cells in WT. We found that both isolates of *SLD3-AID*^-*OsTIR1*^ had a larger population of S phase cells than WT or the AID parent strain. (B) rDNA 2D gel analysis of the same strains in (A). Samples were collected from cycling cells. The asterisk highlights the strains with reduced bubble arc signal compared to WT. The presence of the AID tag correlated with reduced rDNA origin efficiency. Together the flow cytometry and 2D gel analyses demonstrate that the degron tag on Sld3 partially reduces its function.
(PDF)

S5 Fig Replication profiles for all chromosomes from whole genome sequencing (WGS). (A) Illustration of the consequence of unilateral reduction in origin firing efficiency on Marker Frequency Analysis. A hypothetical chromosome with 5 origins that have different timing properties (red, early origins; orange, mid-S origin; yellow, late origins). A collection of cells at different stages in S phase (five of which are shown) produce read depths that have maxima at origins and minima at sites of replication termini. For this model, all cells in the population behave identically. (B) Examples of five chromosomes from a mid-S sample with different subsets of three active origins, but with timing properties preserved. Read depths of all permutations were pooled and normalized to the total read depth of the example in A. The same general features of the replication profiles are preserved while the amplitude between adjacent origins and termini are dampened. (C) WGS-based replication profiles for all chromosomes for *SLD2-*AID. Chromosome coordinates in kb are on the X axis. LOESS-smoothed marker frequency values are on the Y axis. Orange diamonds show OriDB-designated “confirmed” origins [86] and a yellow circle marks the position of each centromere.
(PDF)

S6 Fig Additional 2D gel analysis of origin efficiency. (A) Flow cytometry profiles for asynchronous degron strain cells collected for 2D gel electrophoresis. Orange lines indicate 1C and 2C DNA cells. Depletion of Sld2 enriches the number of cells in S phase. Depletion of Sld3 stalls cells in early S phase and reduces the number of cells in late S/G2. (B) *ARS1209* 2D gel for *SLD3-*AID. The orange arrows point to the bubble and Y arc signal. Although the Y arc and bubble arc are not well-separated on the control blot, there is less replication bubble signal after Sld3 is depleted. (C) 2D gel analysis of late-firing *ARS501/522*. The orange arrows point to the bubble and Y arc signal.
(PDF)

S7 Fig *clb5Δ* AID strain growth and viability on YEPD + 200 mM HU. Cells were serially diluted 1:3, spotted onto YEPD + 200 mM HU, grown at 30°C for seven days and then photographed.
(PDF)

S8 Fig Wild type (W303) ssDNA replication profiles for all chromosomes. Replication profiles (ssDNA S/G1 ratios for the 30 min sample) are shown for all chromosomes. Peaks passing our significance cutoff (see Materials and Methods) are marked with red circles. Yellow circle marks the location of the centromere. Chromosome coordinates are on the X axis and S/G1 ratios are on the Y axis. Values at the rDNA locus (striped box at coordinates 450–500 kb) are excluded because of low probe density on the microarrays.
(PDF)

S9 Fig Wild type, *SLD2*-AID and *SLD3*-AID ssDNA replication assay profiles for all chromosomes. Orange triangle show origins called from WT. Chromosome coordinates are on the X axis and S/G1 ssDNA ratio is mapped on the Y axis. All samples were collected at 60 min. Values at the rDNA locus (striped box at coordinates 450–500 kb) are excluded because of low probe density on the microarrays.
(PDF)

S10 Fig ssDNA peak area correlations for *SLD2-*AID and *SLD3-*AID strains. (A, B) Peak areas for the significant set of 117 origins in uninduced vs. induced *SLD2-*AID (A) and *SLD3-*AID (B) strains. Insets show residuals from linear fit models as a function of fitted values. (C) ssDNA peak area comparison of two untagged, untreated strains (W303 and Anchor Away Parent with untagged *SLD2*). Inset shows residuals from a linear fit model. (D) *CEN*-proximal origin peak area comparison for uninduced *SLD2*-AID compared to induced *SLD2*-AID. *CEN-*proximal origins are the subset of origins assayed by ssDNA assay within 50 kb of the centromere (28 origins). (E) *FKH*-regulated origin peak area comparison for uninduced *SLD2*-AID compared to induced *SLD2*-AID. *FKH-*regulated origins are the subset (84 origins) of origins detected by ssDNA replication profiling identified by Knott, et al. [27] as partially regulated by *FKH1* and *FKH2* transcription factor binding near origins. All samples were collected at 60 min.
(PDF)

S11 Fig *SLD2* Anchor Away strain cell cycle progression, peak area correlation, and ssDNA replication assay profiles for all chromosomes. (A) S phase progression analysis by flow cytometry for Anchor Away parent and *SLD2* Anchor Away cells with and without the addition of rapamycin. (B) ssDNA replication profile for chromosome XII in wild type control and rapamycin-treated Anchor Away parent strain. The wild type profile is the same as shown in Fig 6. (C) WT ssDNA peak areas compared to those seen in rapamycin-treated Anchor Away parent strain cells. (D) *CEN*-proximal origin peak area comparison for induced *SLD2* Anchor Away compared to WT (28 origins). (E) *FKH*-regulated origin peak area comparison for uninduced *SLD2* Anchor Away compared to induced *SLD2* Anchor Away (84 origins). (F) *SLD2* Anchor Away ssDNA replication profiles for all chromosomes. All samples were collected at 30 min. WT profiles are the same as in S8 Fig.
(PDF)

S12 Fig Flow cytometry and ssDNA replication assay profiles for all chromosomes in wild type and in SSDD and SSDDCS overexpression strains. (A) We analyzed synchronous S phase progression by flow cytometry for SSDD and SSDDCS overexpression strains without HU. Times indicate the time after release from α-factor arrest. The blue line marks the position of cells with 1C DNA. (B) ssDNA replication profiles for wild type and overexpression strains of SSDD and SSDDCS collected at 120 min. See Fig 8 legend for details. Orange triangles show origins called from WT. Yellow circle marks the centromere. Chromosome coordinates are on the X axis and S/G1 ssDNA ratio is mapped on the Y axis.
(PDF)

S1 Table Oligonucleotides used in this study. (XLSX)

S2 Table Strains used in this study. (XLSX)

S3 Table Subtelomeric regions excluded in marker frequency analysis. (XLSX)

S4 Table Ohnologs and Ty elements excluded in marker frequency analysis. (XLSX)

S5 Table Origins analyzed by ssDNA mapping. (XLSX)

S6 Table Key resources used. (XLSX)

S1 Data *SLD2*-AID minus and plus auxin 1kb bin WGS smoothed data. (XLSX)

S2 Data Wild type ssDNA data. Zip archive.
(ZIP)

S3 Data *SLD2-AID* minus and plus auxin ssDNA data. Zip archive.
(ZIP)

S4 Data *SLD3*-AID minus and plus auxin ssDNA data. Zip archive.
(ZIP)

S5 Data *SLD2*-AnchorAway ssDNA data. Zip archive.
(ZIP)

S6 Data Anchor Away Parent strain ssDNA data. Zip archive.
(ZIP)

S7 Data SSDD overexpression strain ssDNA data. Zip archive.
(ZIP)

S8 Data SSDDCS overexpression strain ssDNA data. Zip archive.
(ZIP)

S9 Data Peak area values for scatter plots. (XLSX)

S10 Data Heat map data. (XLSX)

S11 Data Data from growth curves, viability analysis, and quantification of CHEF gels and 2D gels. (XLSX)
