# Supplementary figures and images for: The effects of manipulating levels of replication initiation factors on origin firing efficiency in yeast
Source: PLoS Genet. 2019 Oct 4;15(10):e1008430. doi: 10.1371/journal.pgen.1008430 (PMC6795477; doi:10.1371/journal.pgen.1008430)

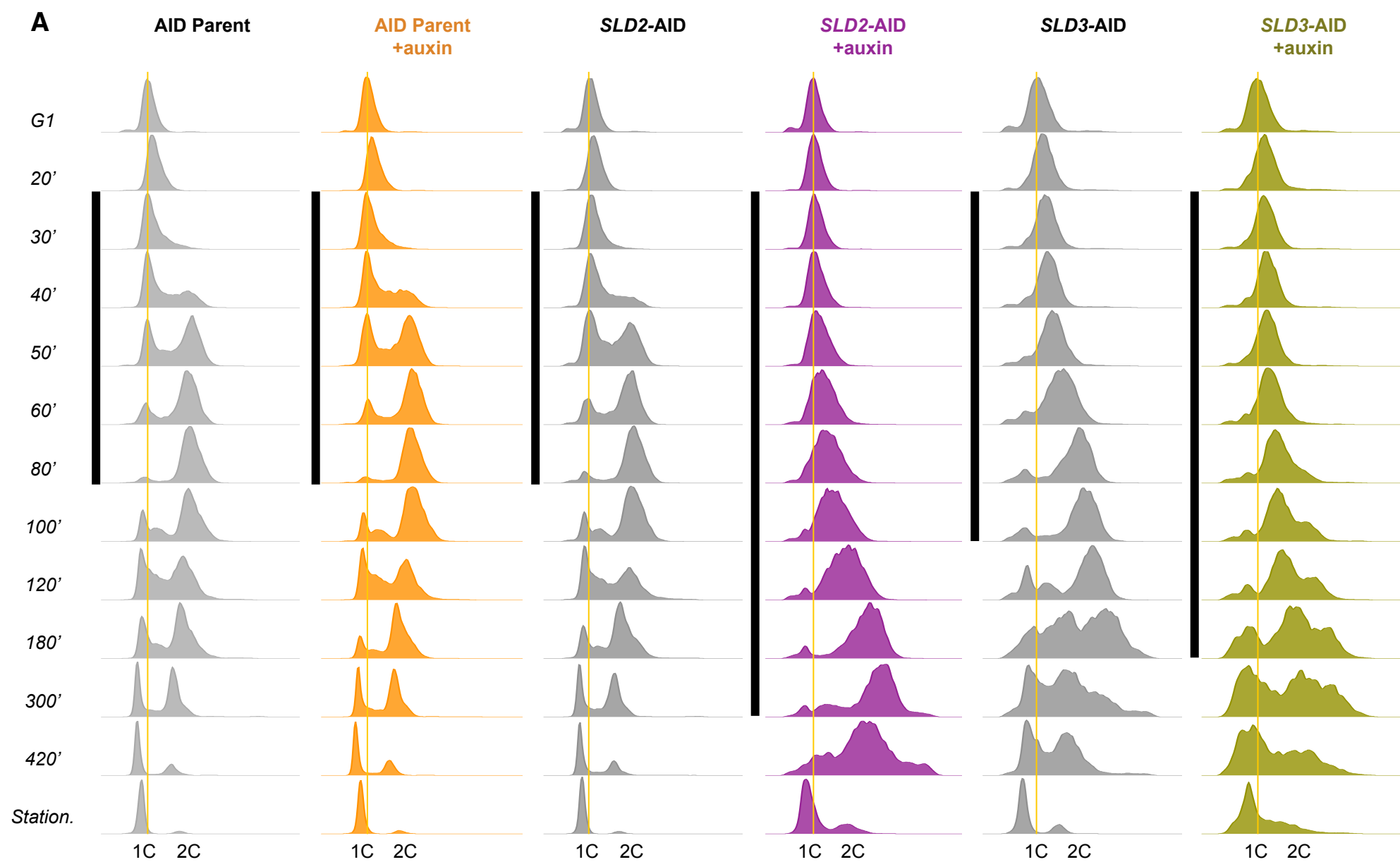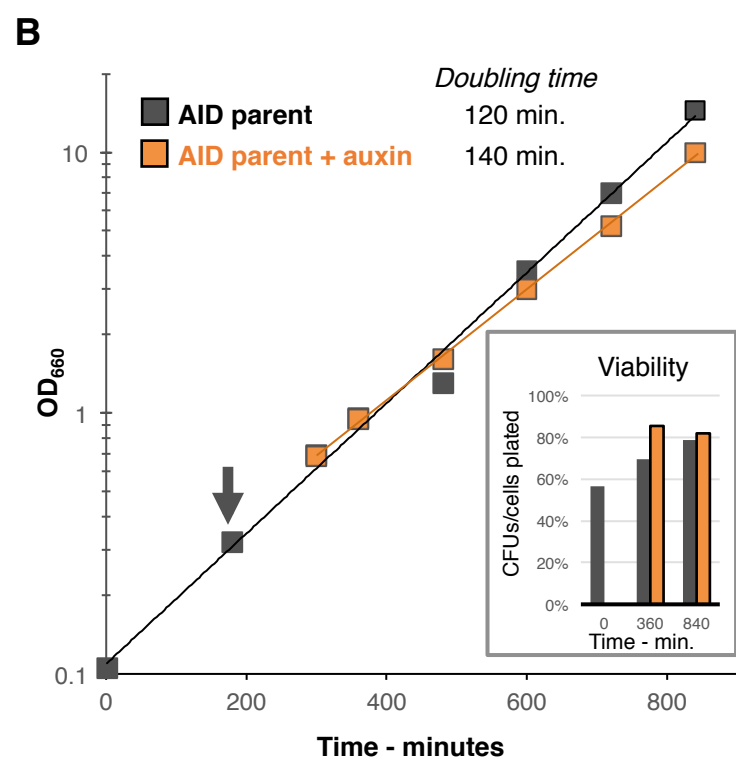

**C**

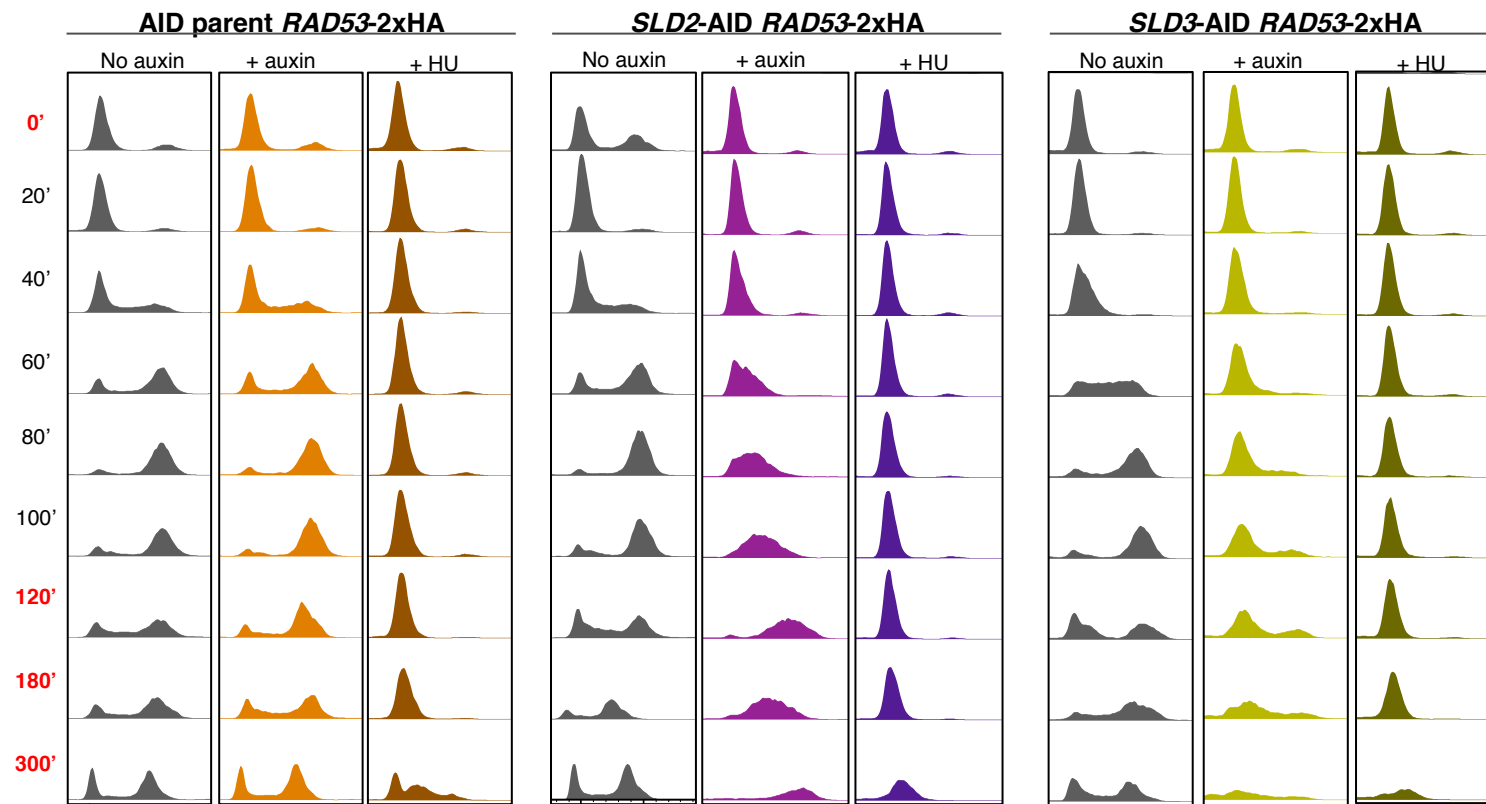

**D**

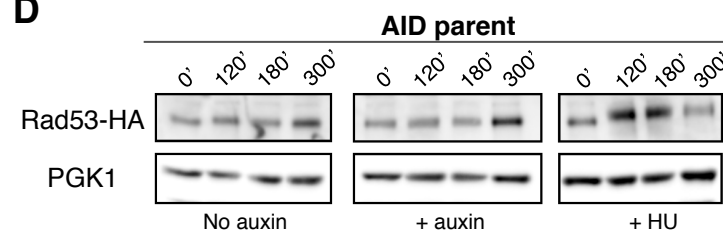

Supplement: S1 Fig — (A) We tested cell cycle progression in the AID parent strain to determine whether any off-target auxin-induced protein degradation changed S phase progression. We have included induced and uninduced SLD2-AID and SLD3-AID strains (Fig 2) for comparison. The thin orange line marks the position of cells with 1C DNA and the thick vertical black bars indicate the approximate duration of S phase in each condition. (B) AID parent strain growth. The auxin-treated cells had a slightly slower growth rate (17% increase) but auxin did not impact viability. (C) Flow cytometry profiles for the AID strains for each condition used for western blotting of Rad53 phosphorylation in a RAD53-2xHA strain background. Bold, red text indicates the samples analyzed by western blot. (D) Rad53 phosphorylation in the AID parent strain. (PDF) [file pgen.1008430.s001.pdf]

***SLD2-AID + auxin***

*120'*

*240'*

*Stationary phase  
culture*

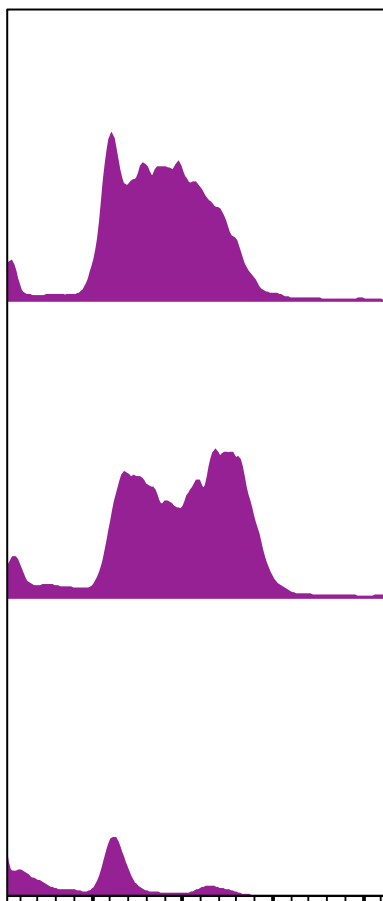

Supplement: S3 Fig — Profiles for asynchronous SLD2-AID cells treated with auxin for 120 minutes, 240 minutes, and grown to saturation overnight. (PDF) [file pgen.1008430.s003.pdf]

**A**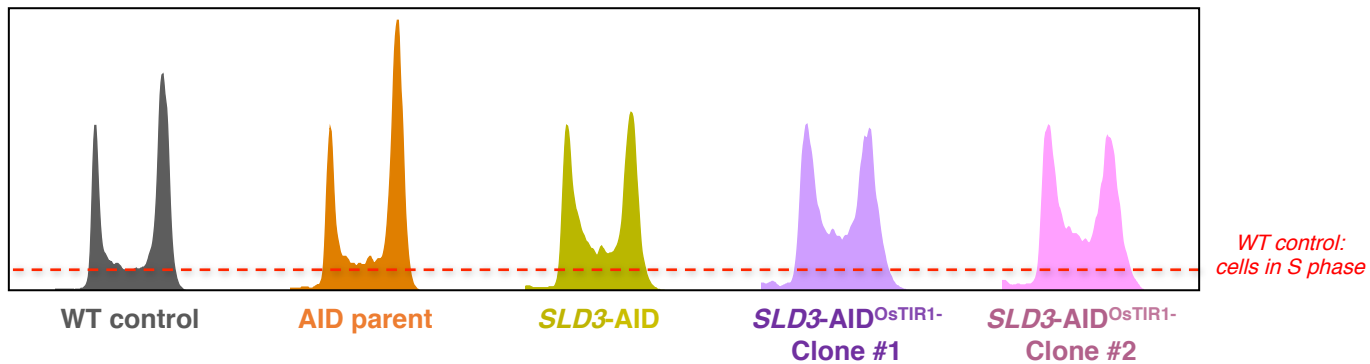**B**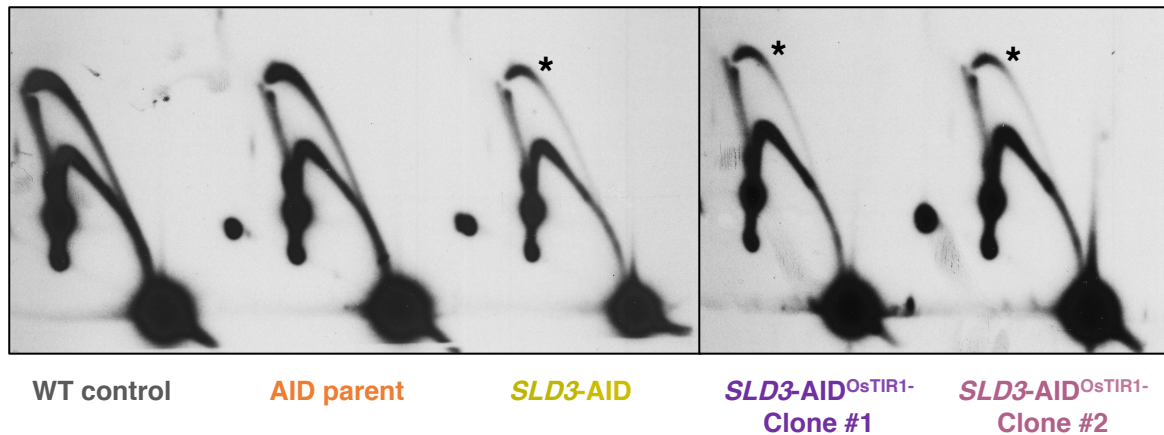

Supplement: S4 Fig — To test for compromised Sld3 function, we constructed a version of the SLD3-AID strain without the E3 ubiquitin ligase. (A) Flow cytometry profiles for asynchronous growth in WT, AID parent, SLD3-AID, and two clones containing degron-tagged Sld3 but no GAL1-10-OsTIR1 construct. The G1 peaks are height-matched, and the dashed line indicates the proportion of S phase cells in WT. We found that both isolates of SLD3-AID-OsTIR1 had a larger population of S phase cells than WT or the AID parent strain. (B) rDNA 2D gel analysis of the same strains in (A). Samples were collected from cycling cells. The asterisk highlights the strains with reduced bubble arc signal compared to WT. The presence of the AID tag correlated with reduced rDNA origin efficiency. Together the flow cytometry and 2D gel analyses demonstrate that the degron tag on Sld3 partially reduces its function. (PDF) [file pgen.1008430.s004.pdf]

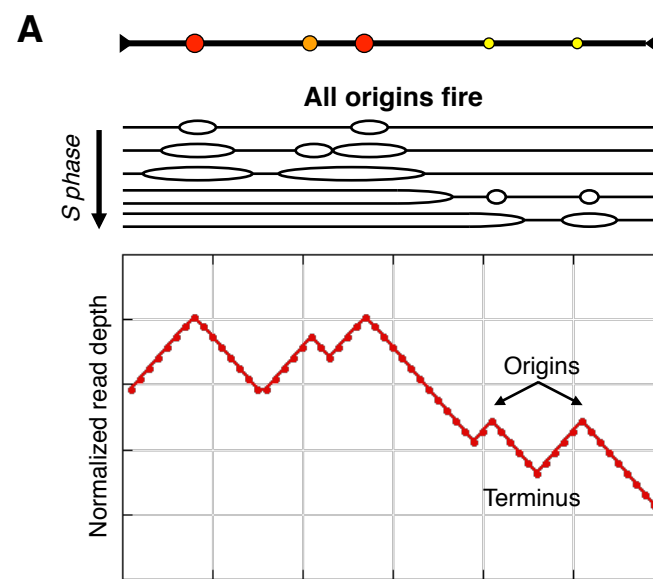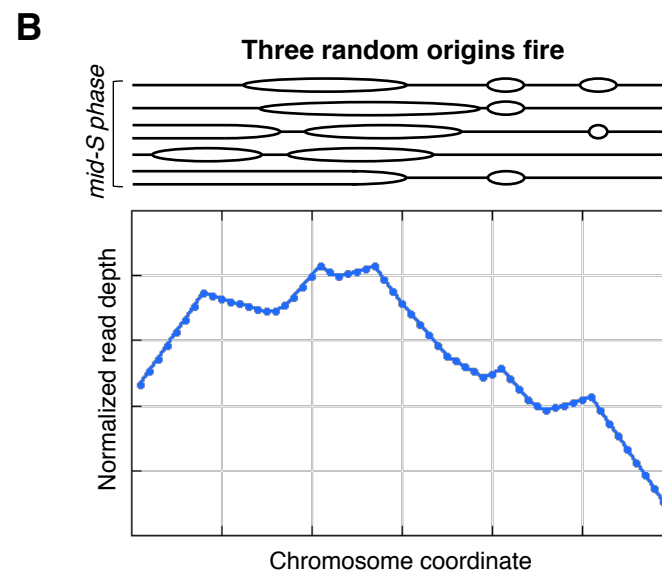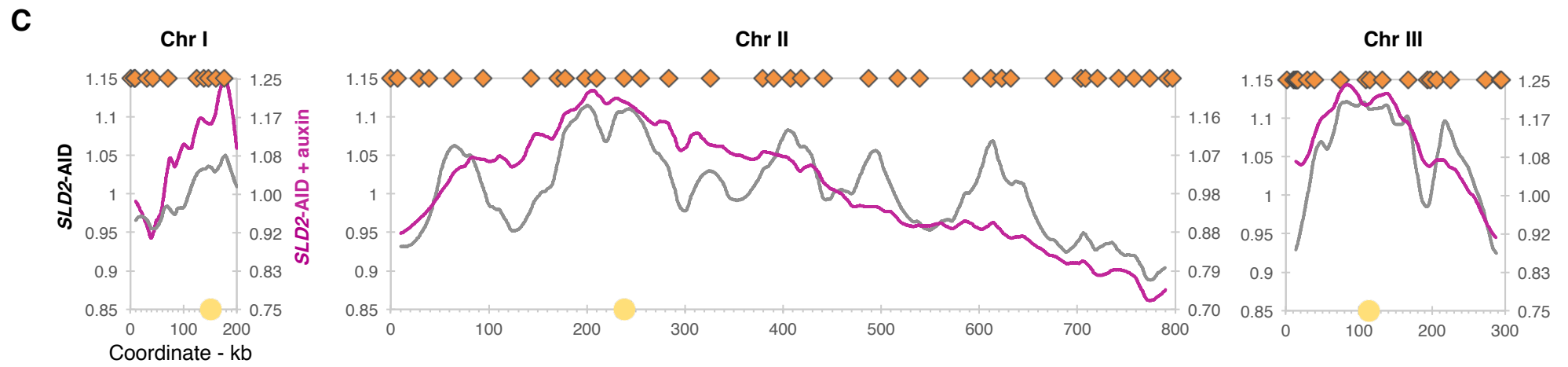

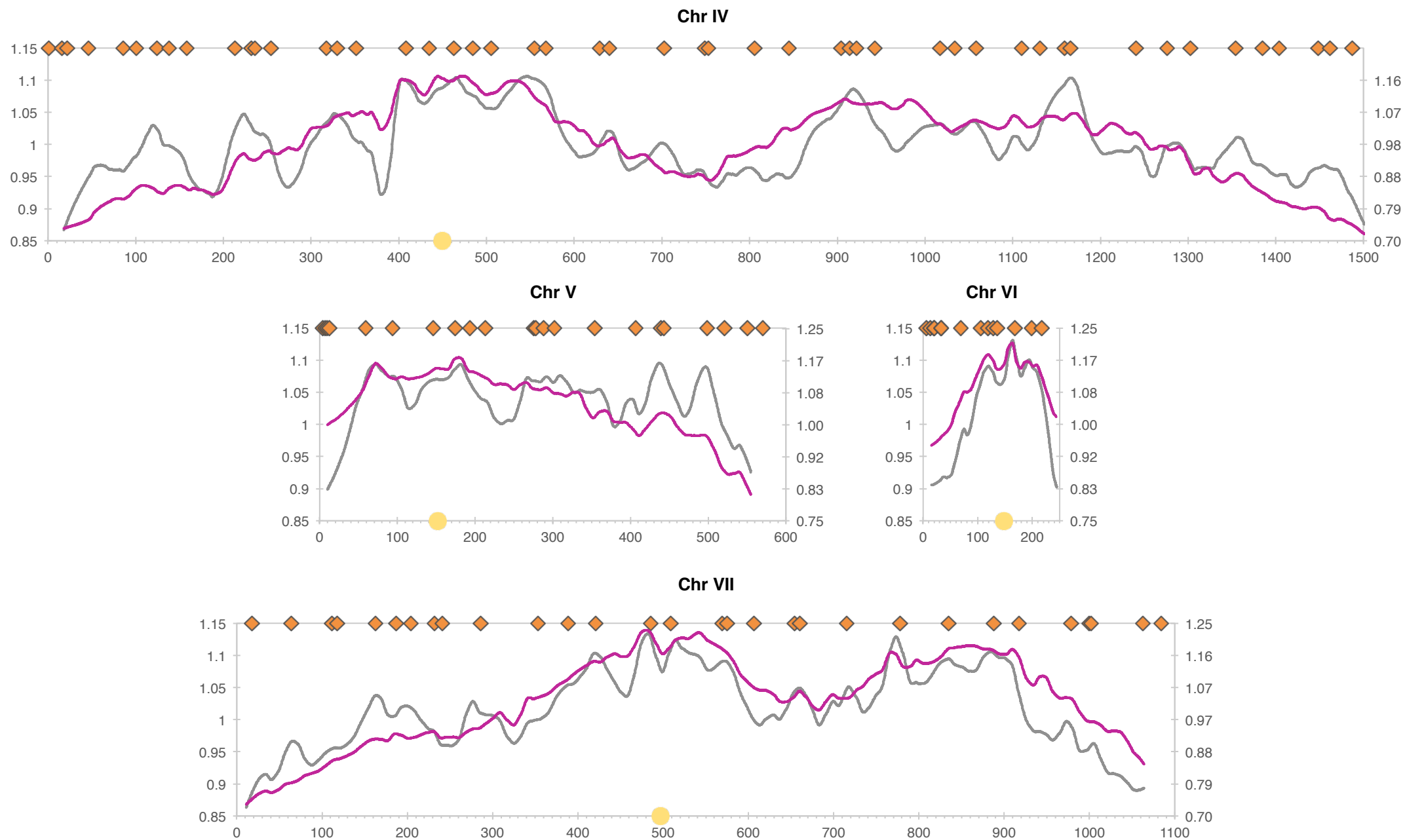

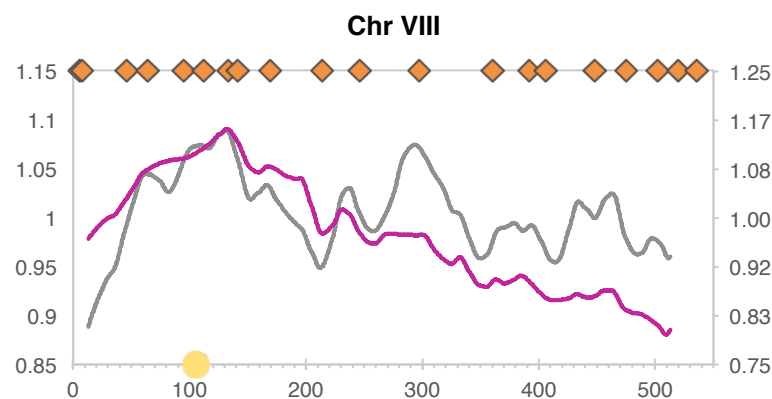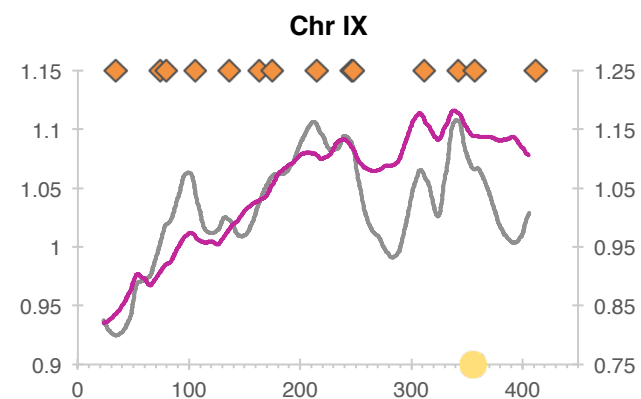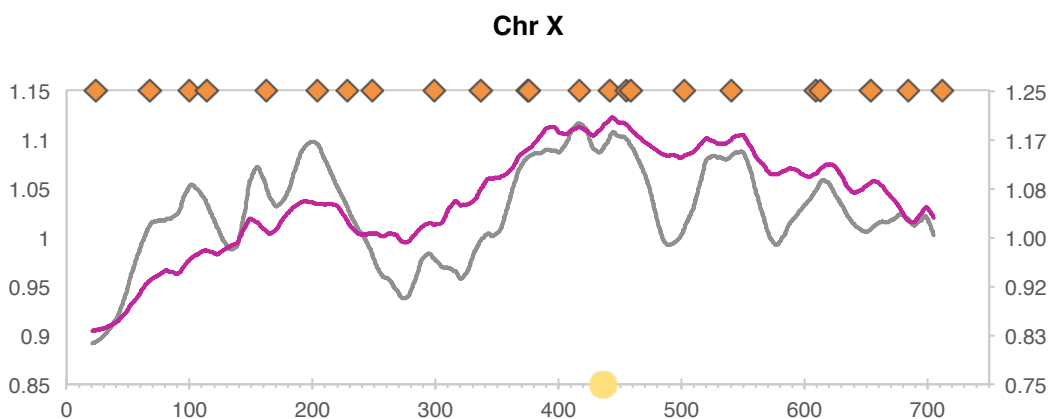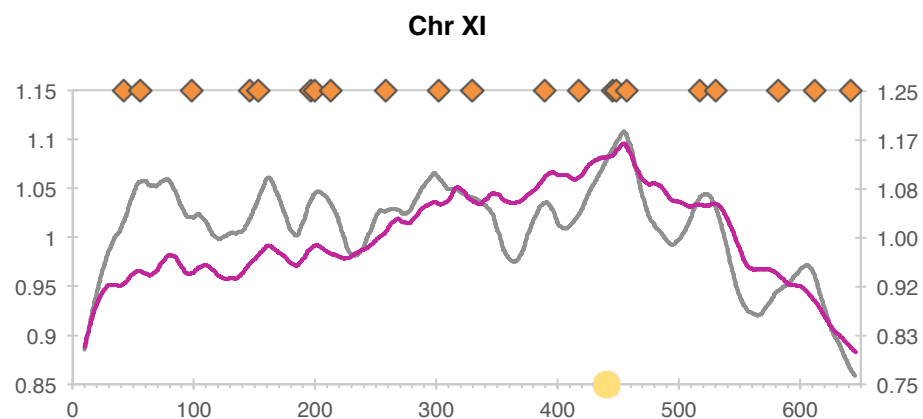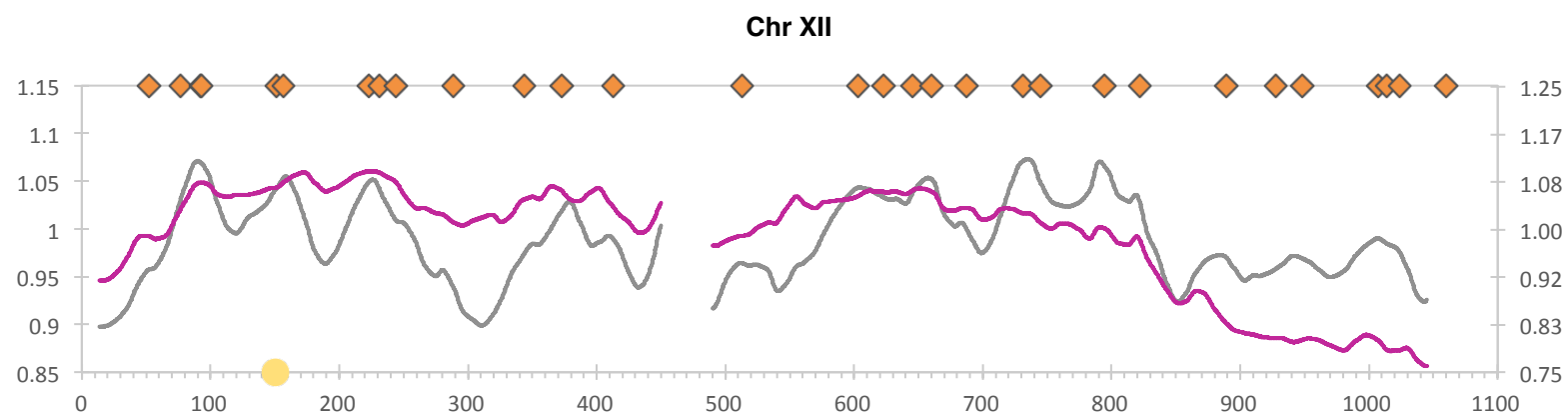

Chr XIII

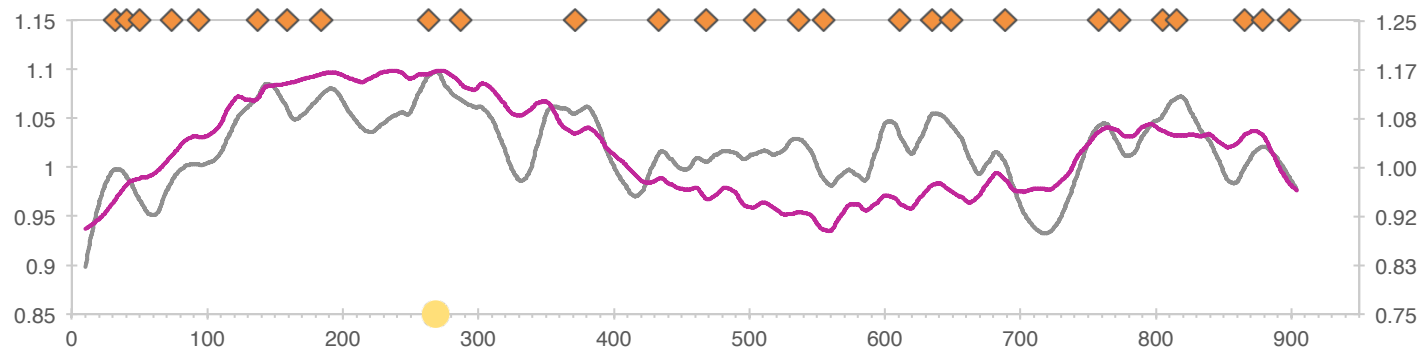

Chr XIV

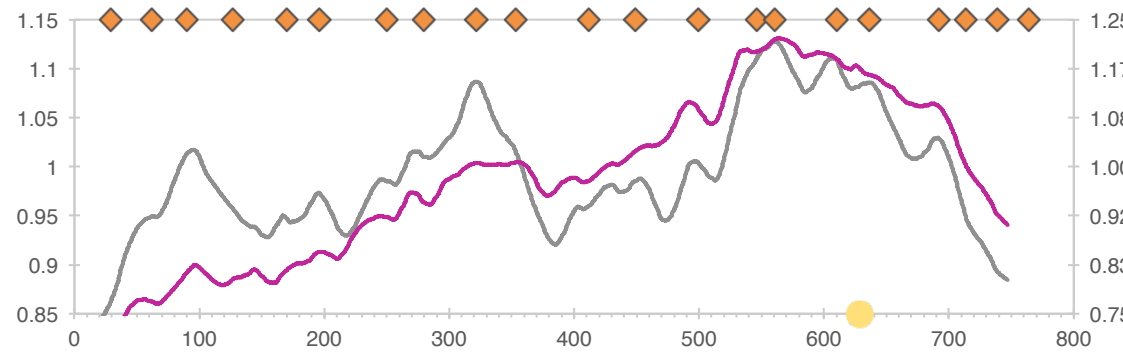

Chr XV

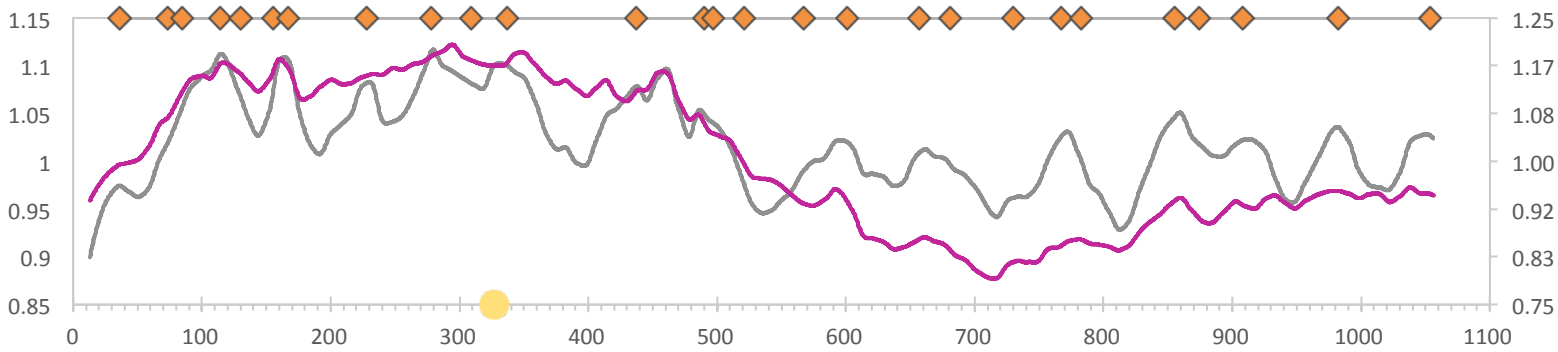

Chr XVI

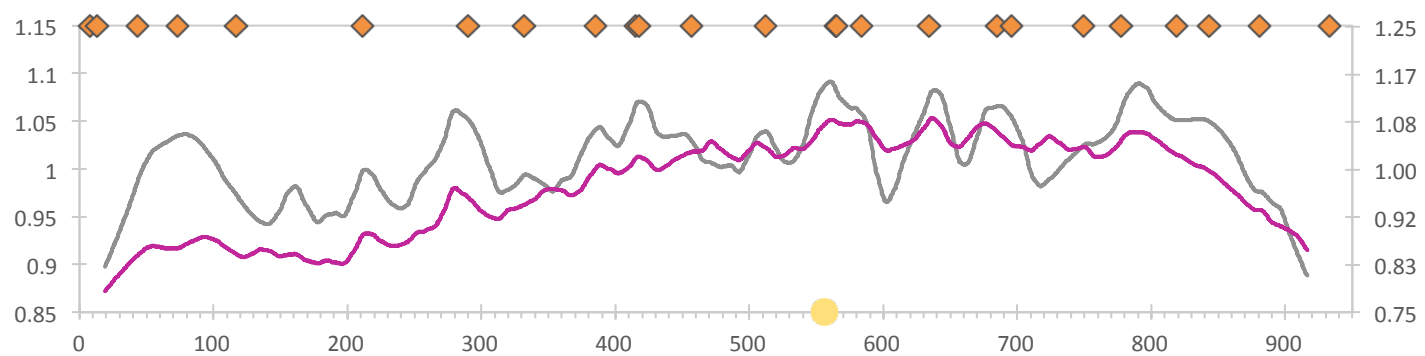

Supplement: S5 Fig — (A) Illustration of the consequence of unilateral reduction in origin firing efficiency on Marker Frequency Analysis. A hypothetical chromosome with 5 origins that have different timing properties (red, early origins; orange, mid-S origin; yellow, late origins). A collection of cells at different stages in S phase (five of which are shown) produce read depths that have maxima at origins and minima at sites of replication termini. For this model, all cells in the population behave identically. (B) Examples of five chromosomes from a mid-S sample with different subsets of three active origins, but with timing properties preserved. Read depths of all permutations were pooled and normalized to the total read depth of the example in A. The same general features of the replication profiles are preserved while the amplitude between adjacent origins and termini are dampened. (C) WGS-based replication profiles for all chromosomes for SLD2-AID. Chromosome coordinates in kb are on the X axis. LOESS-smoothed marker frequency values are on the Y axis. Orange diamonds show OriDB-designated “confirmed” origins [86] and a yellow circle marks the position of each centromere. (PDF) [file pgen.1008430.s005.pdf]

**A**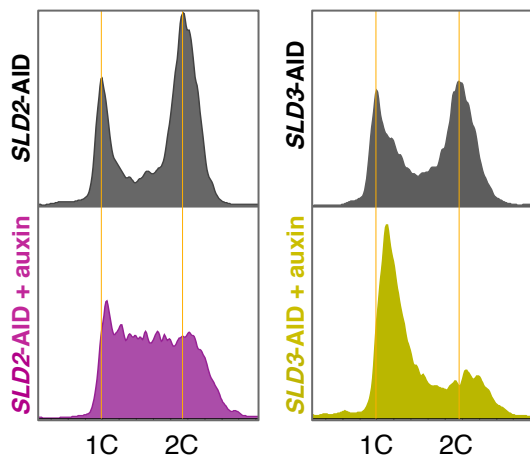**B***ARS1209*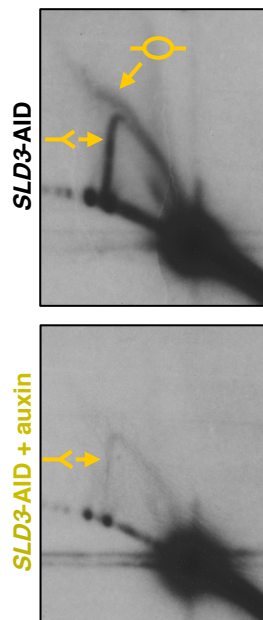**C***ARS522*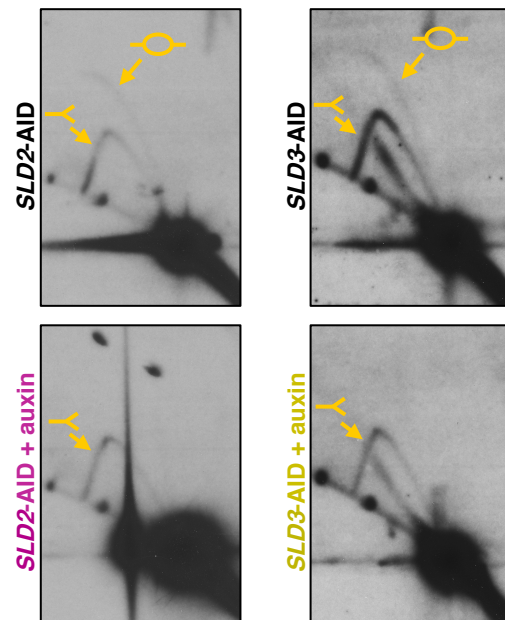

Supplement: S6 Fig — (A) Flow cytometry profiles for asynchronous degron strain cells collected for 2D gel electrophoresis. Orange lines indicate 1C and 2C DNA cells. Depletion of Sld2 enriches the number of cells in S phase. Depletion of Sld3 stalls cells in early S phase and reduces the number of cells in late S/G2. (B) ARS1209 2D gel for SLD3-AID. The orange arrows point to the bubble and Y arc signal. Although the Y arc and bubble arc are not well-separated on the control blot, there is less replication bubble signal after Sld3 is depleted. (C) 2D gel analysis of late-firing ARS501/522. The orange arrows point to the bubble and Y arc signal. (PDF) [file pgen.1008430.s006.pdf]

200 mM HU

**WT**

**AID Parent**

***SLD2-AID***

**AID Parent *clb5Δ***

***SLD2-AID clb5Δ***

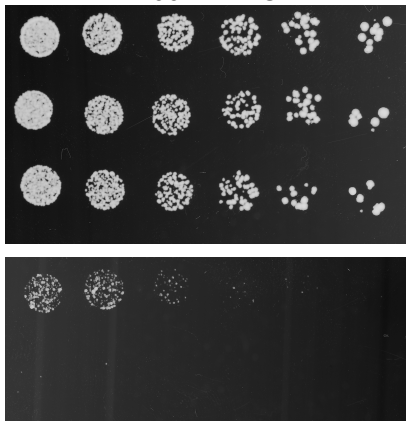

Supplement: S7 Fig — Cells were serially diluted 1:3, spotted onto YEPD + 200 mM HU, grown at 30°C for seven days and then photographed. (PDF) [file pgen.1008430.s007.pdf]

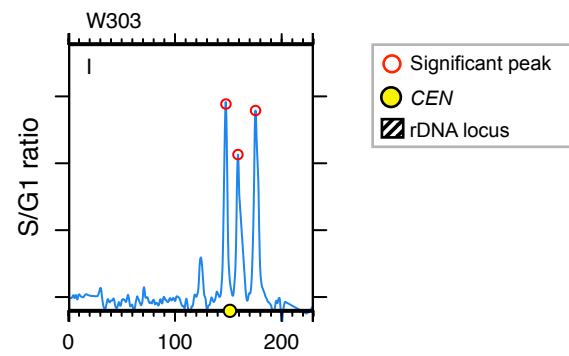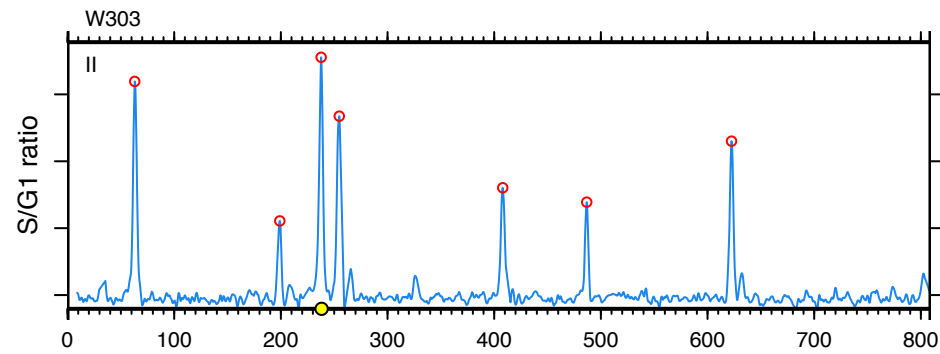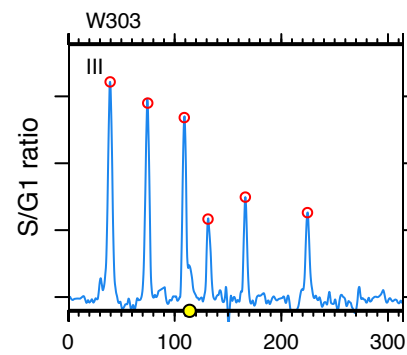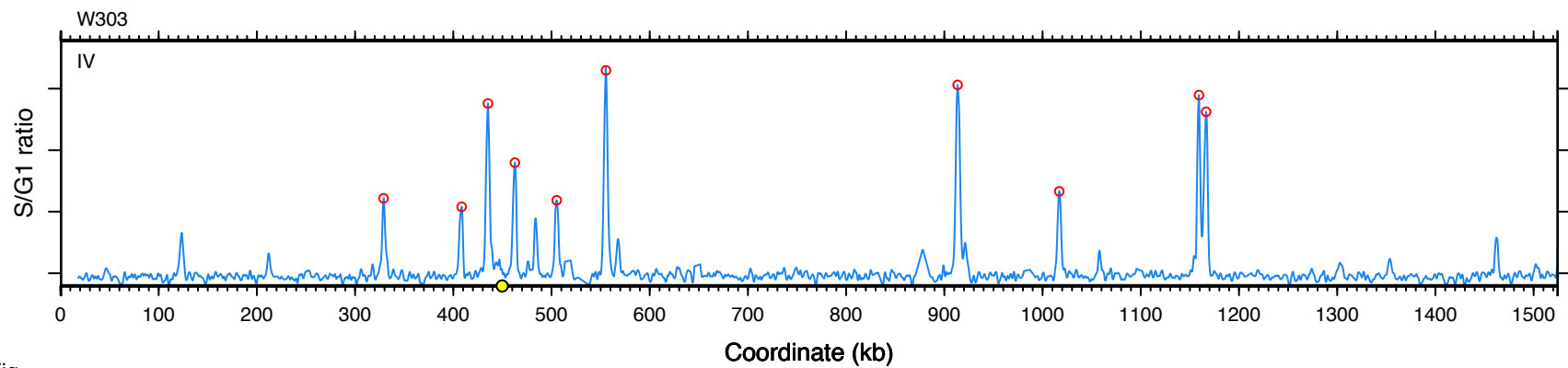

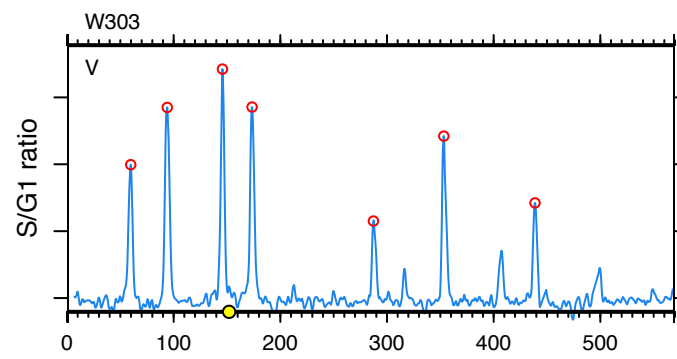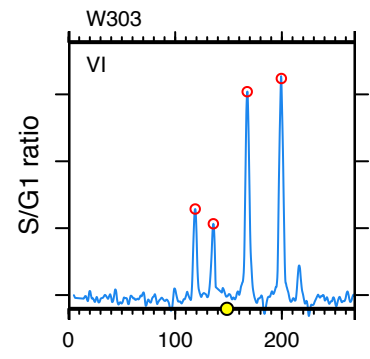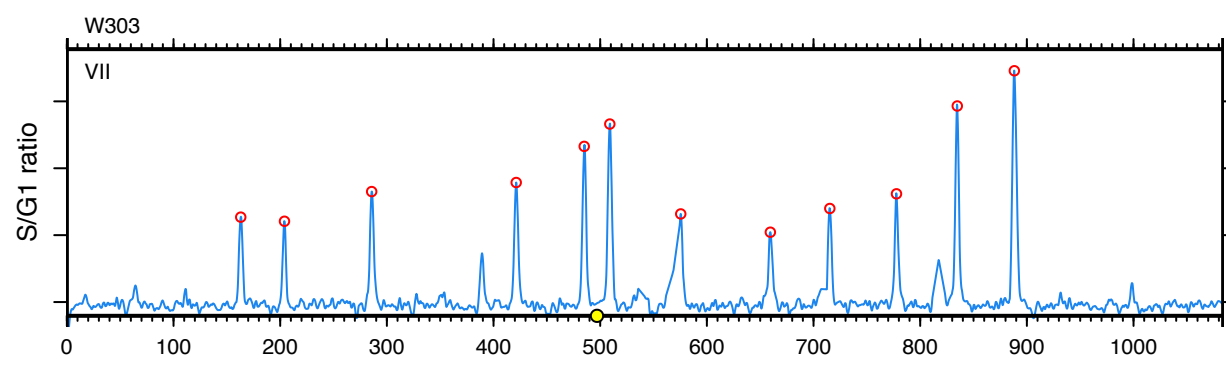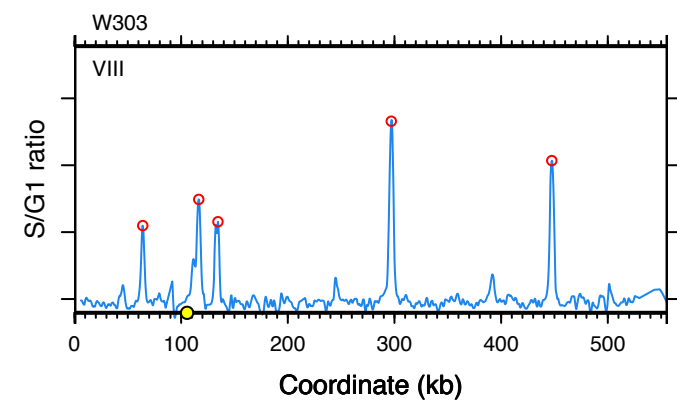

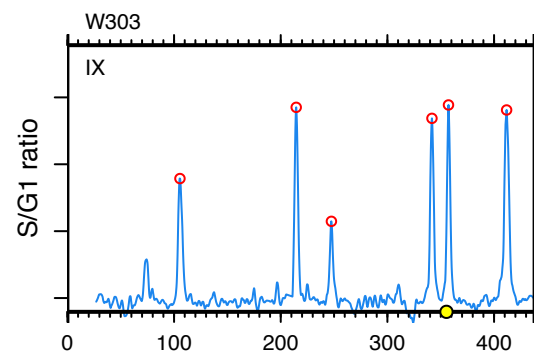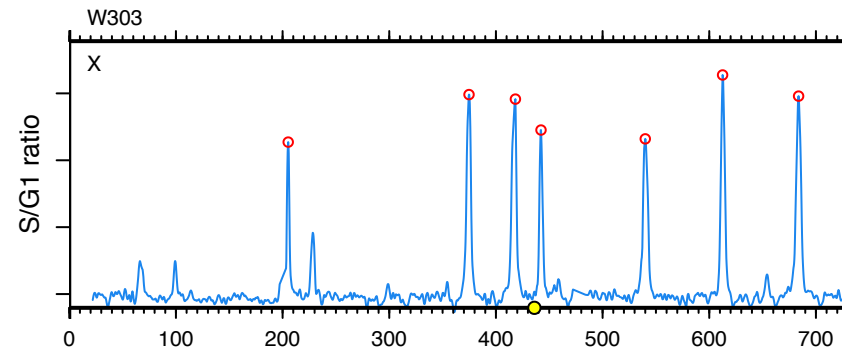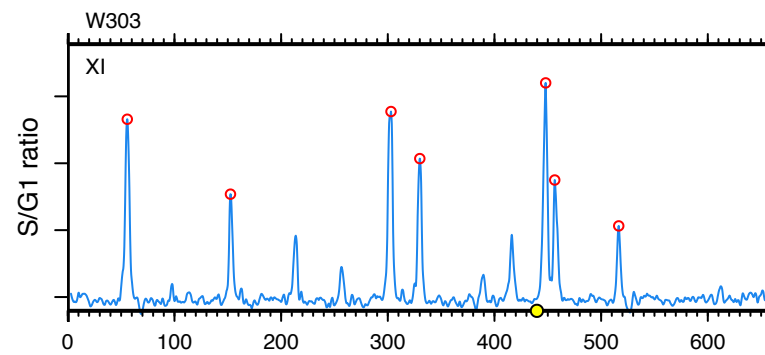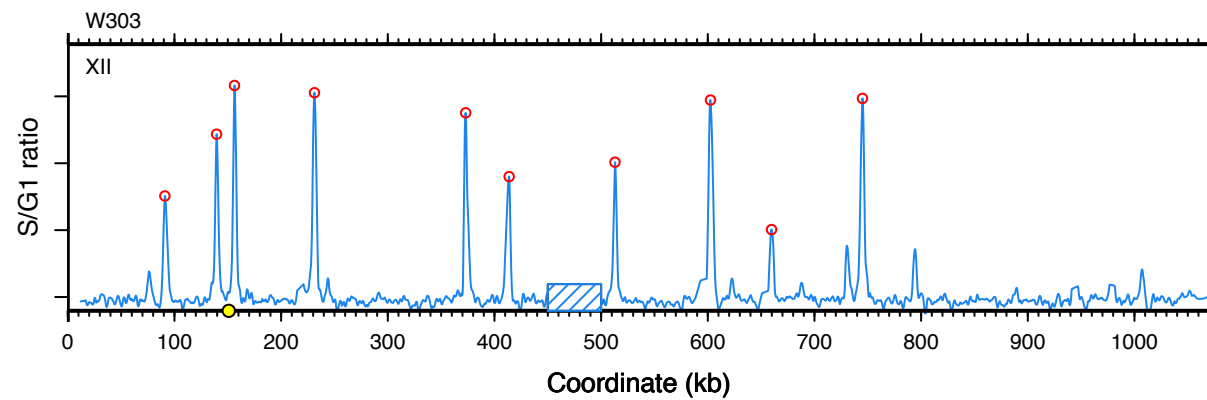

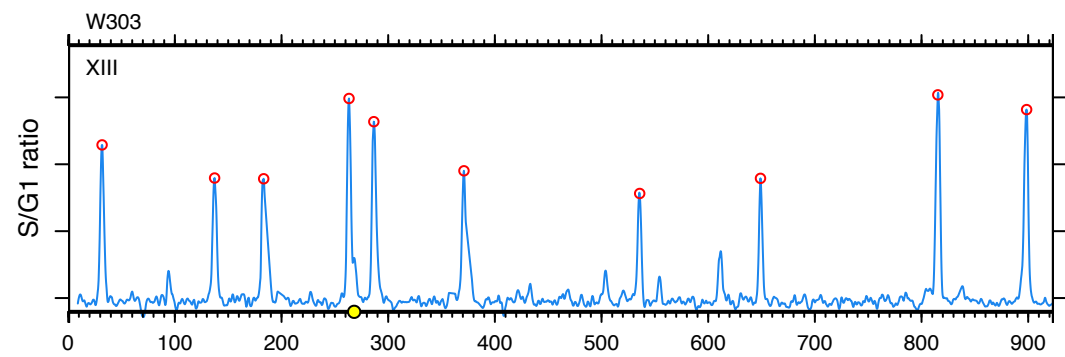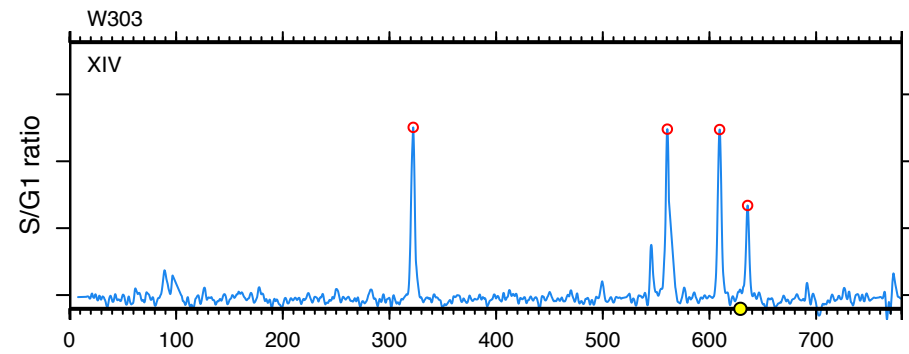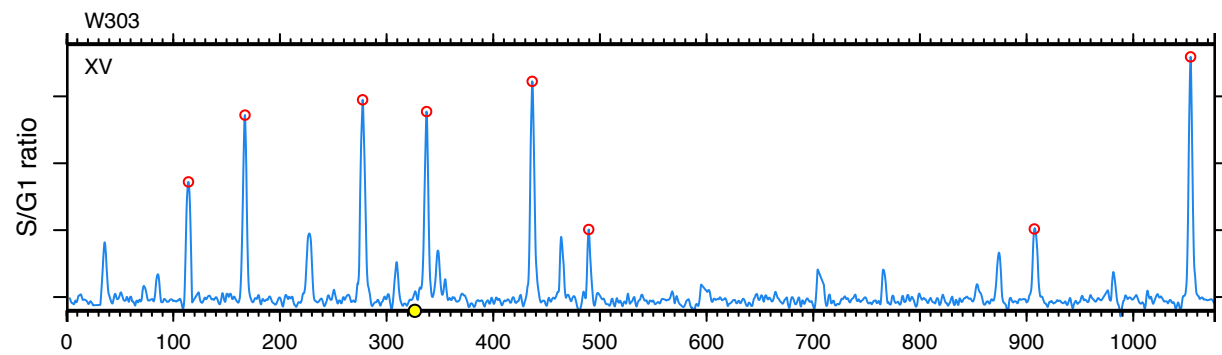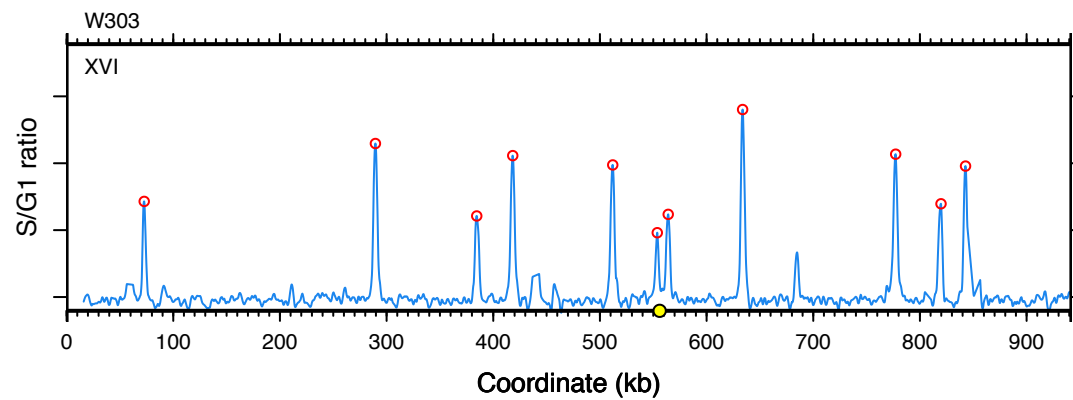

Supplement: S8 Fig — Replication profiles (ssDNA S/G1 ratios for the 30 min sample) are shown for all chromosomes. Peaks passing our significance cutoff (see Materials and Methods) are marked with red circles. Yellow circle marks the location of the centromere. Chromosome coordinates are on the X axis and S/G1 ratios are on the Y axis. Values at the rDNA locus (striped box at coordinates 450–500 kb) are excluded because of low probe density on the microarrays. (PDF) [file pgen.1008430.s008.pdf]

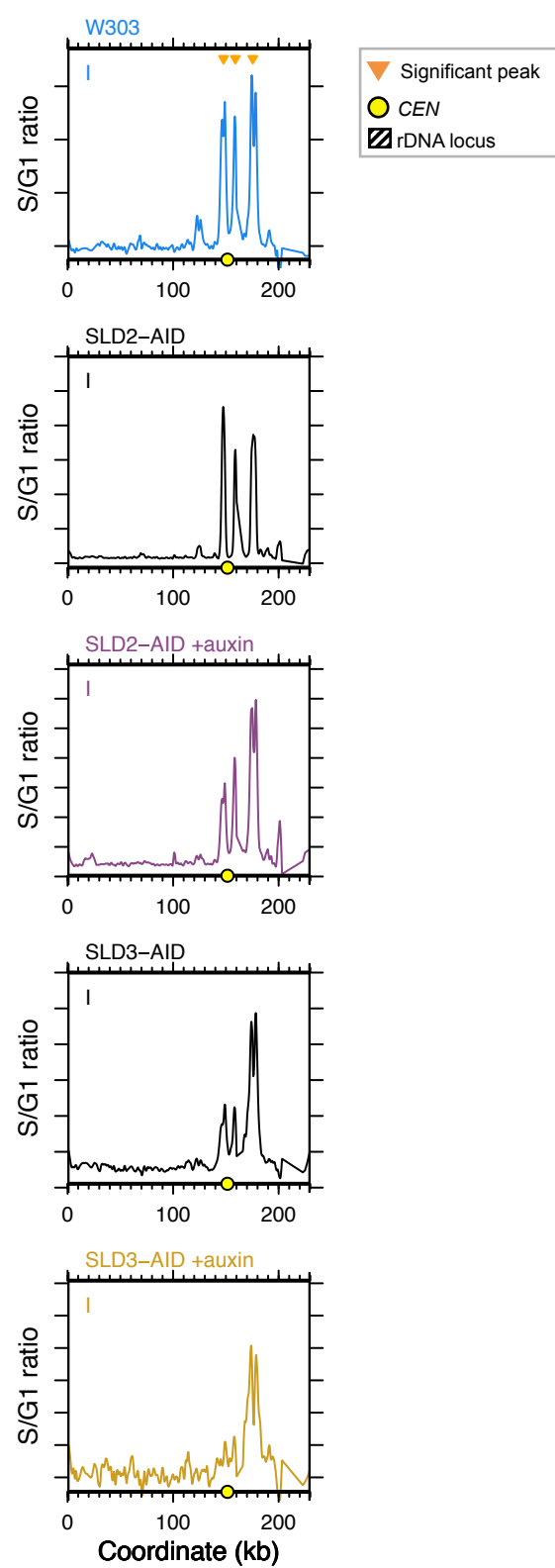

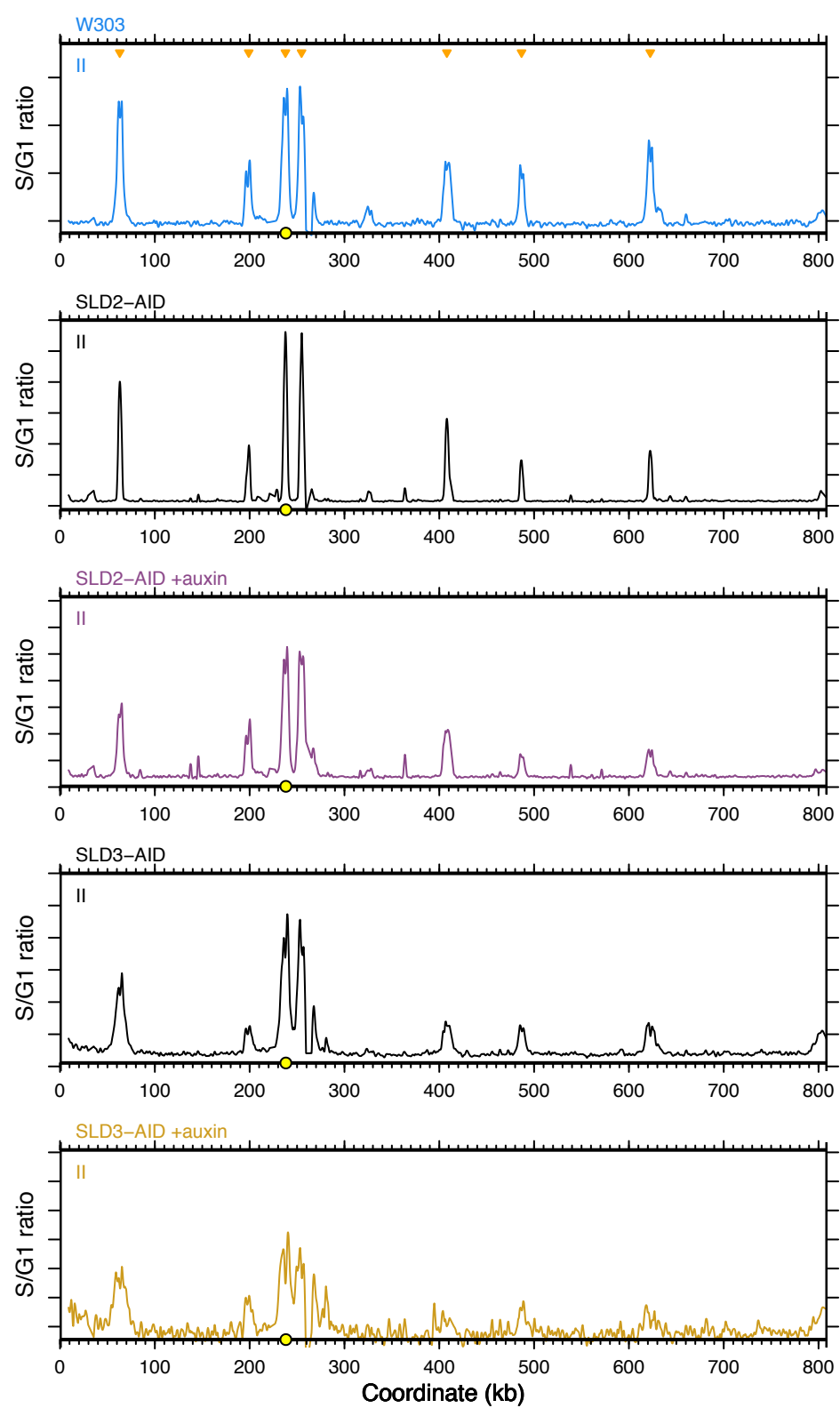

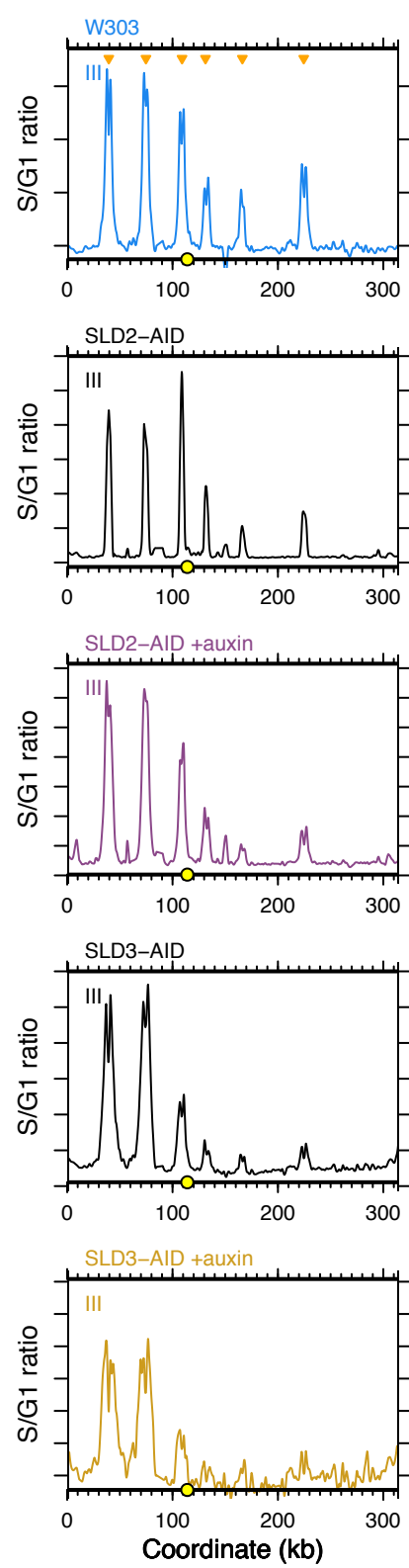

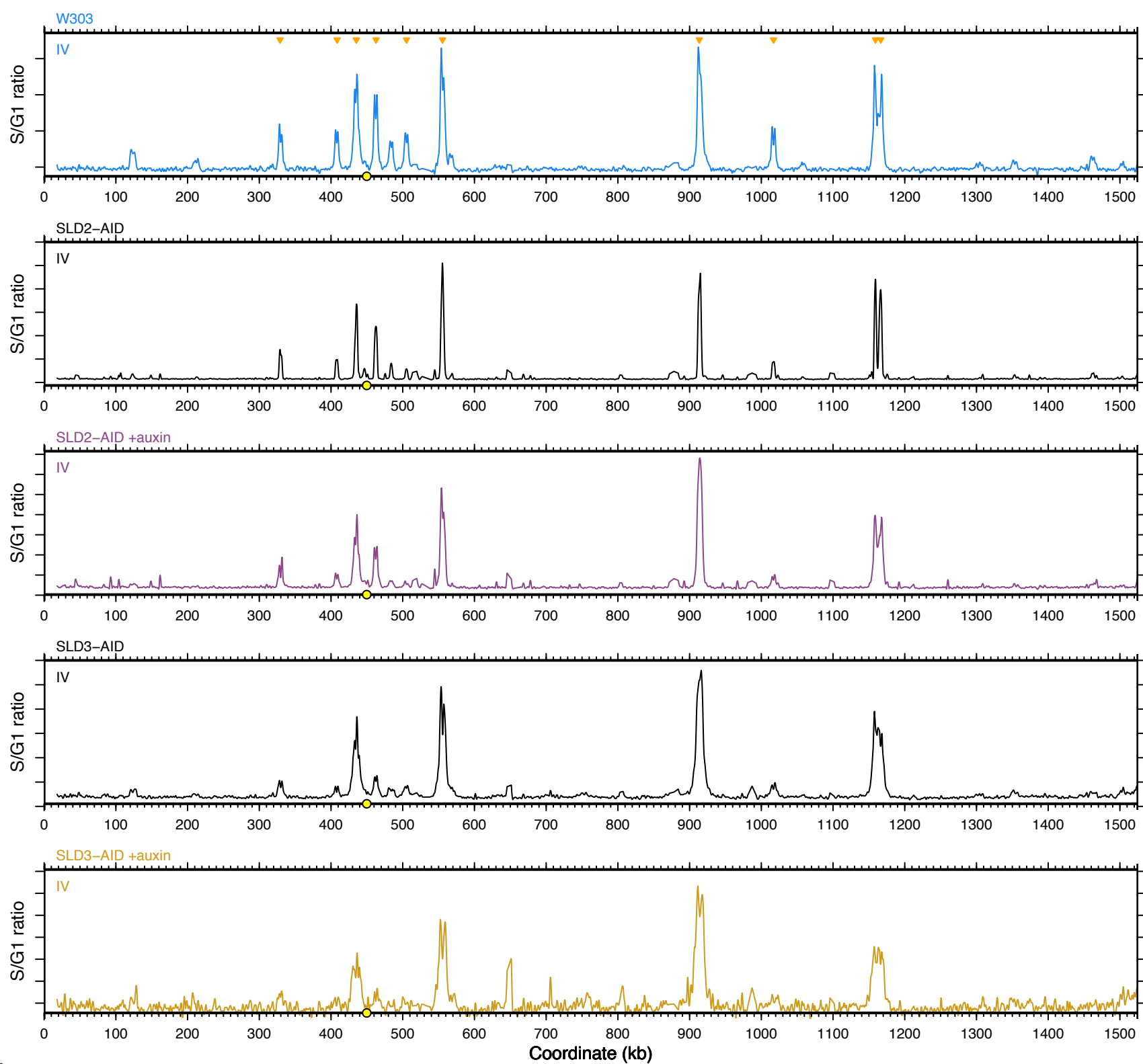

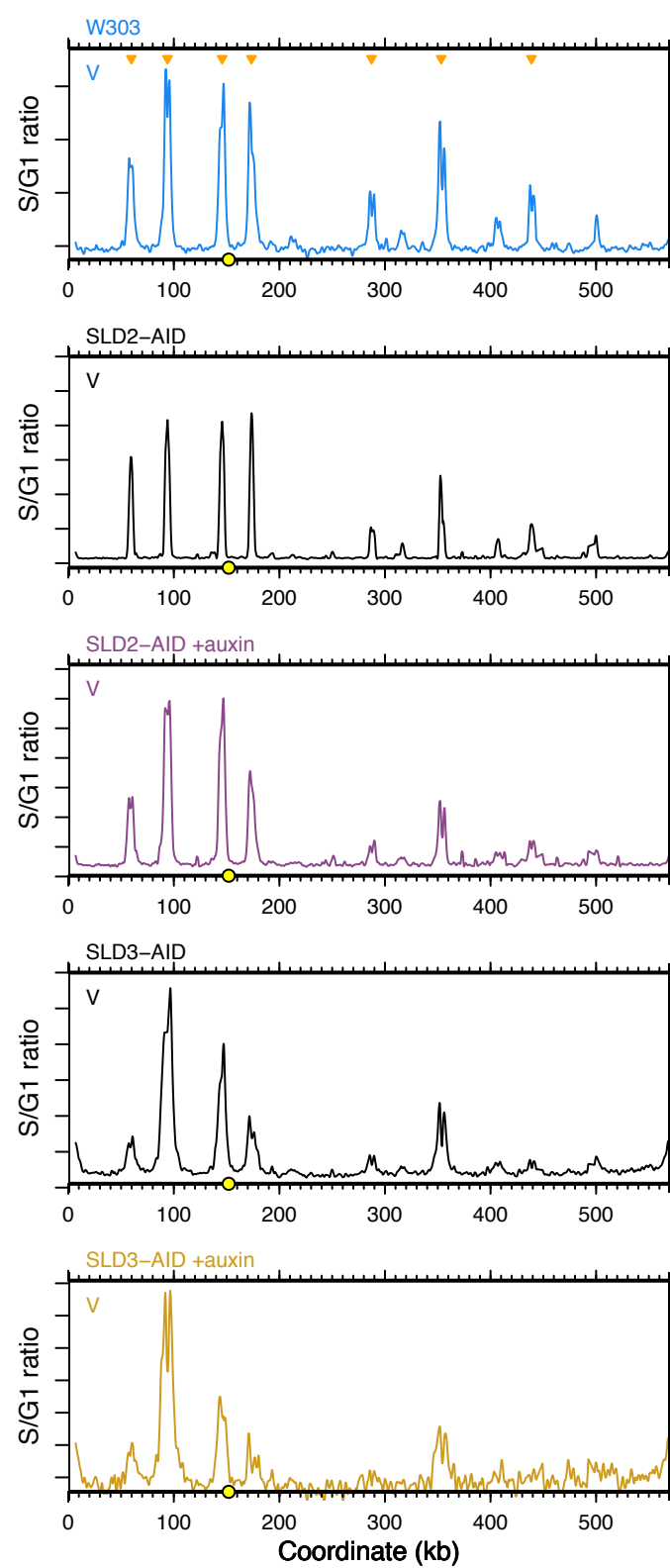

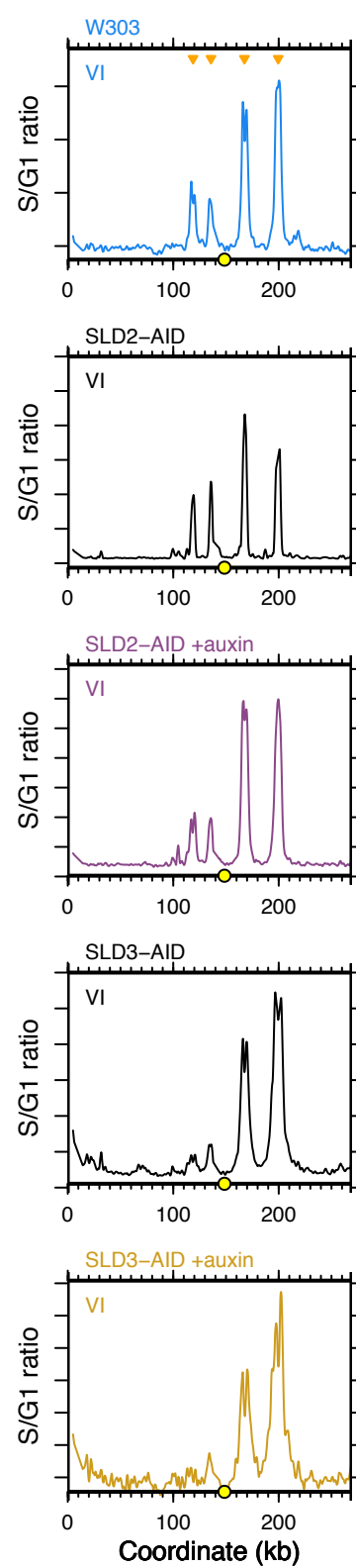

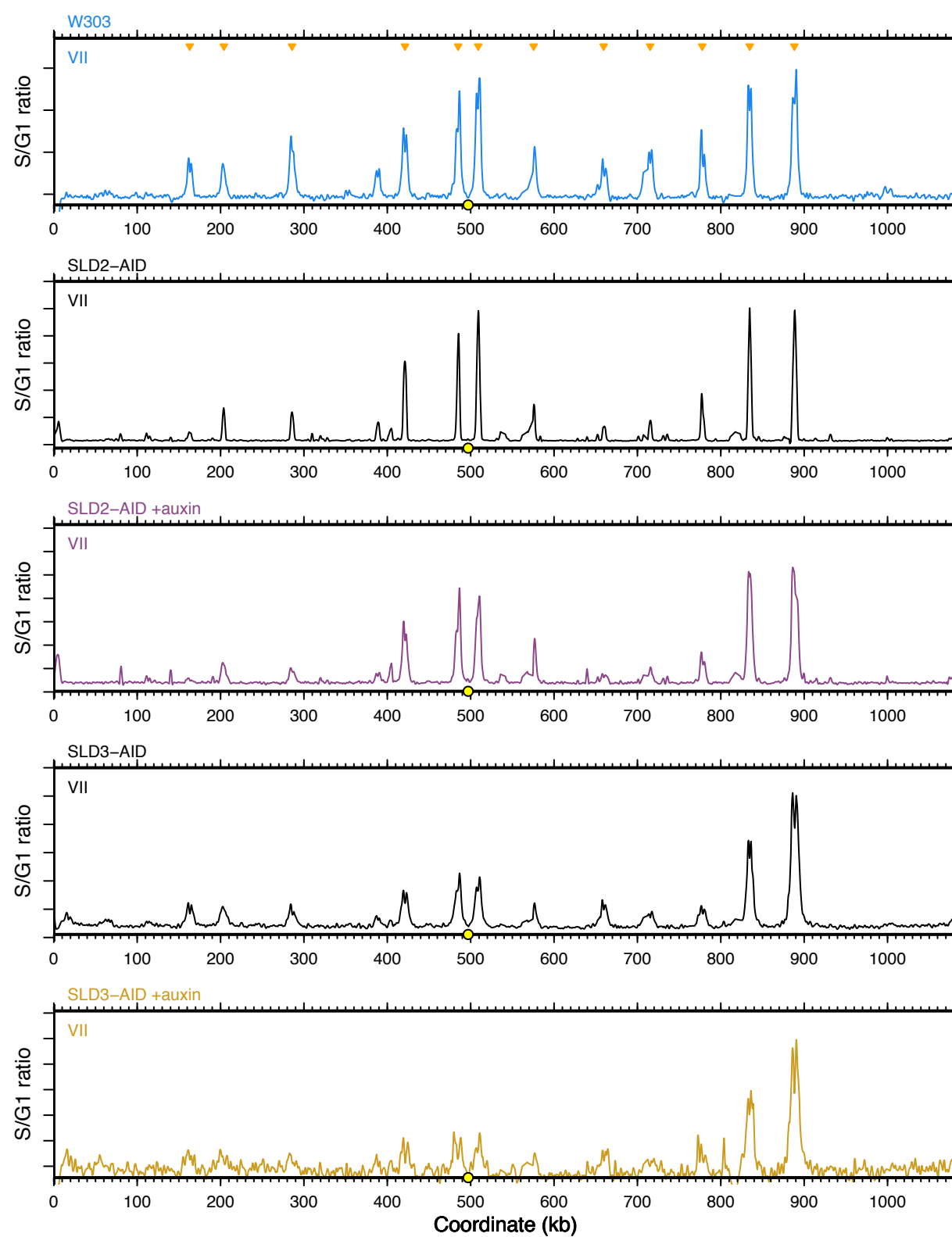

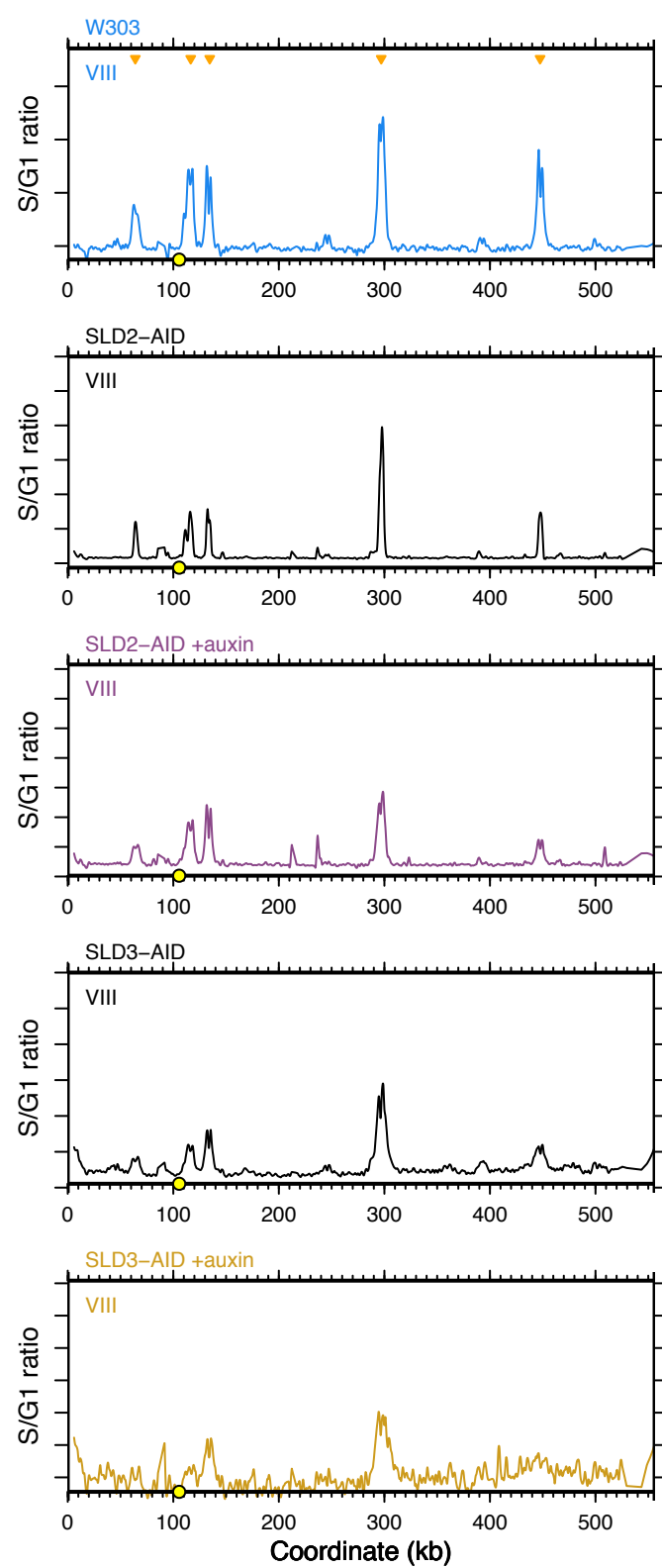

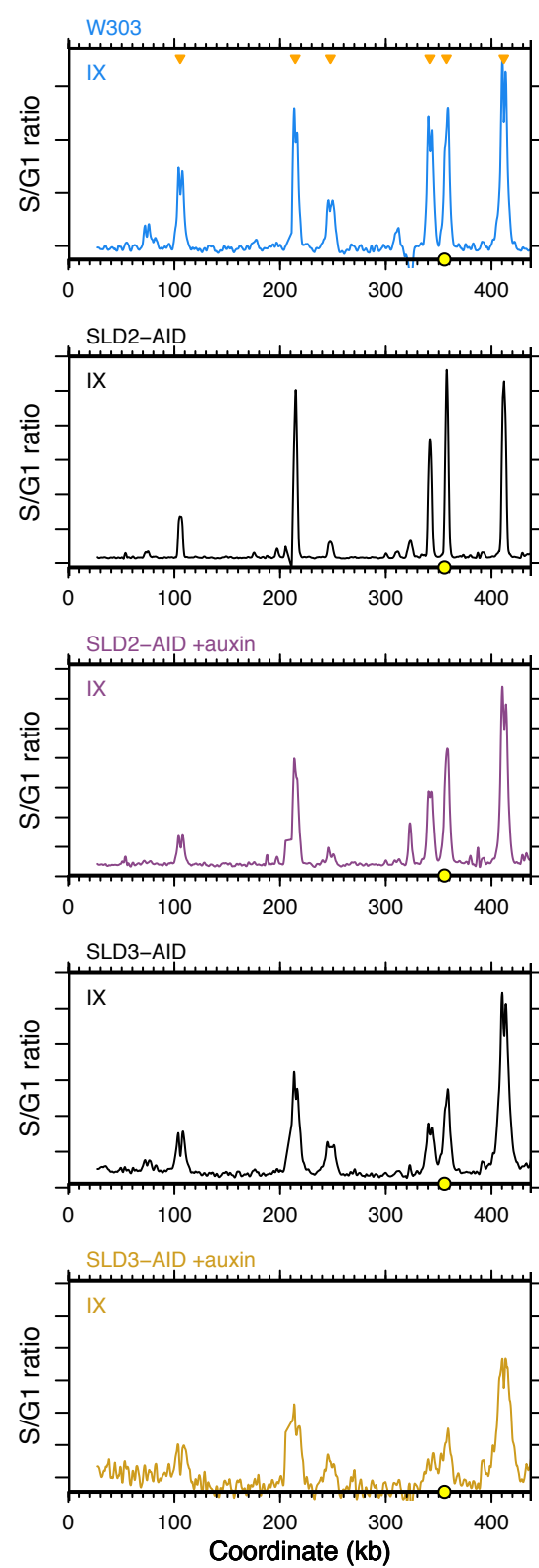

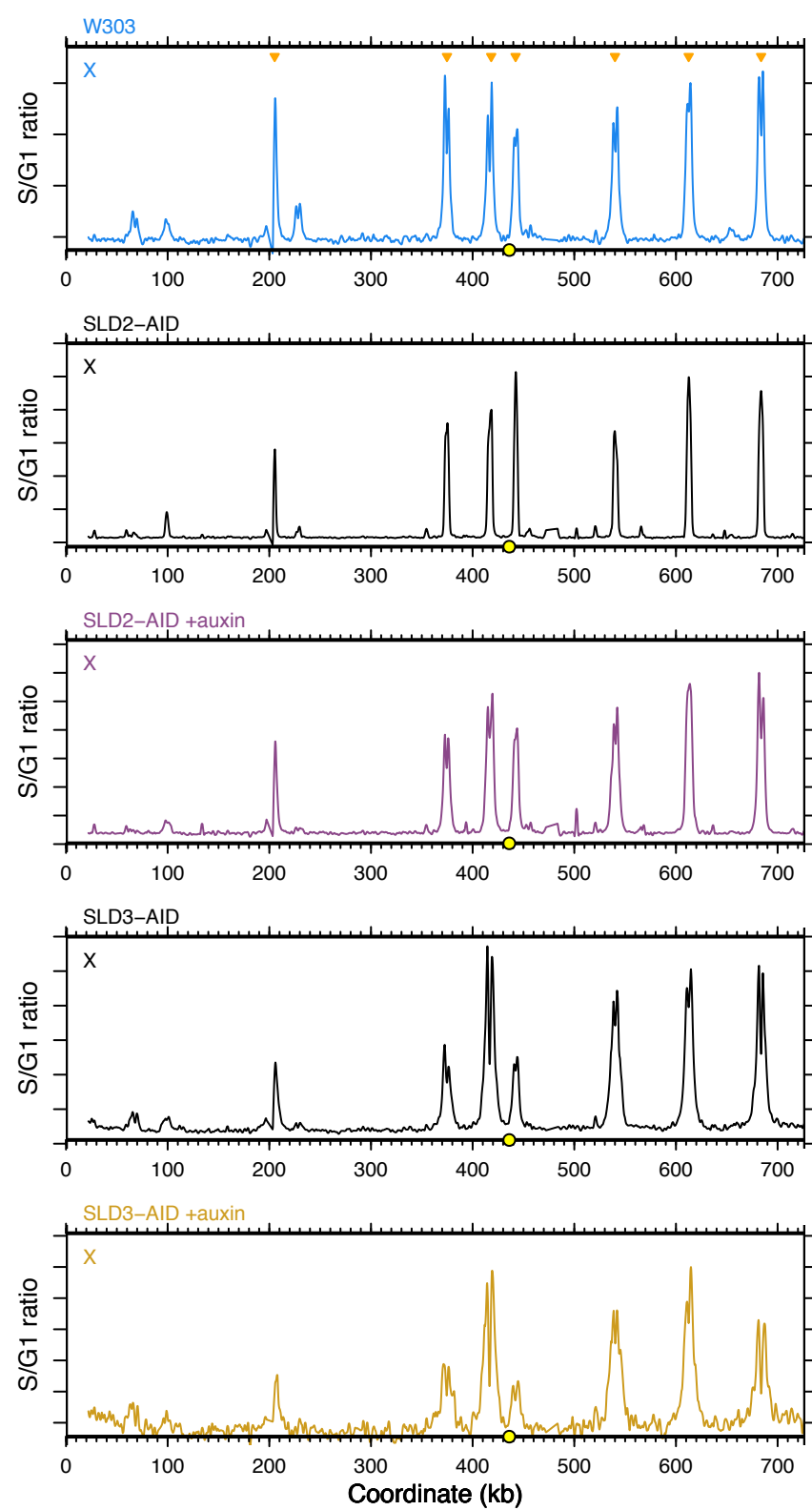

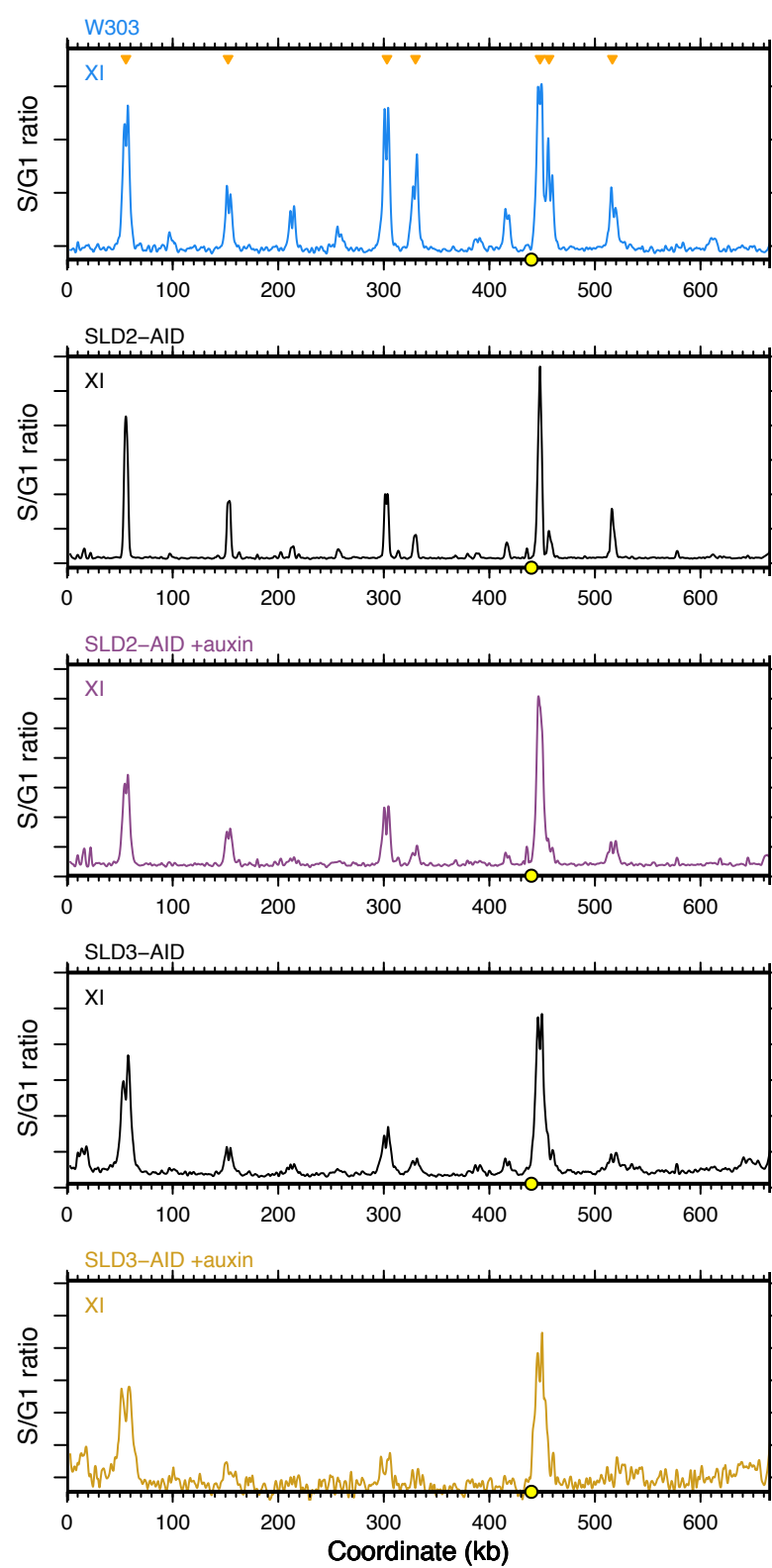

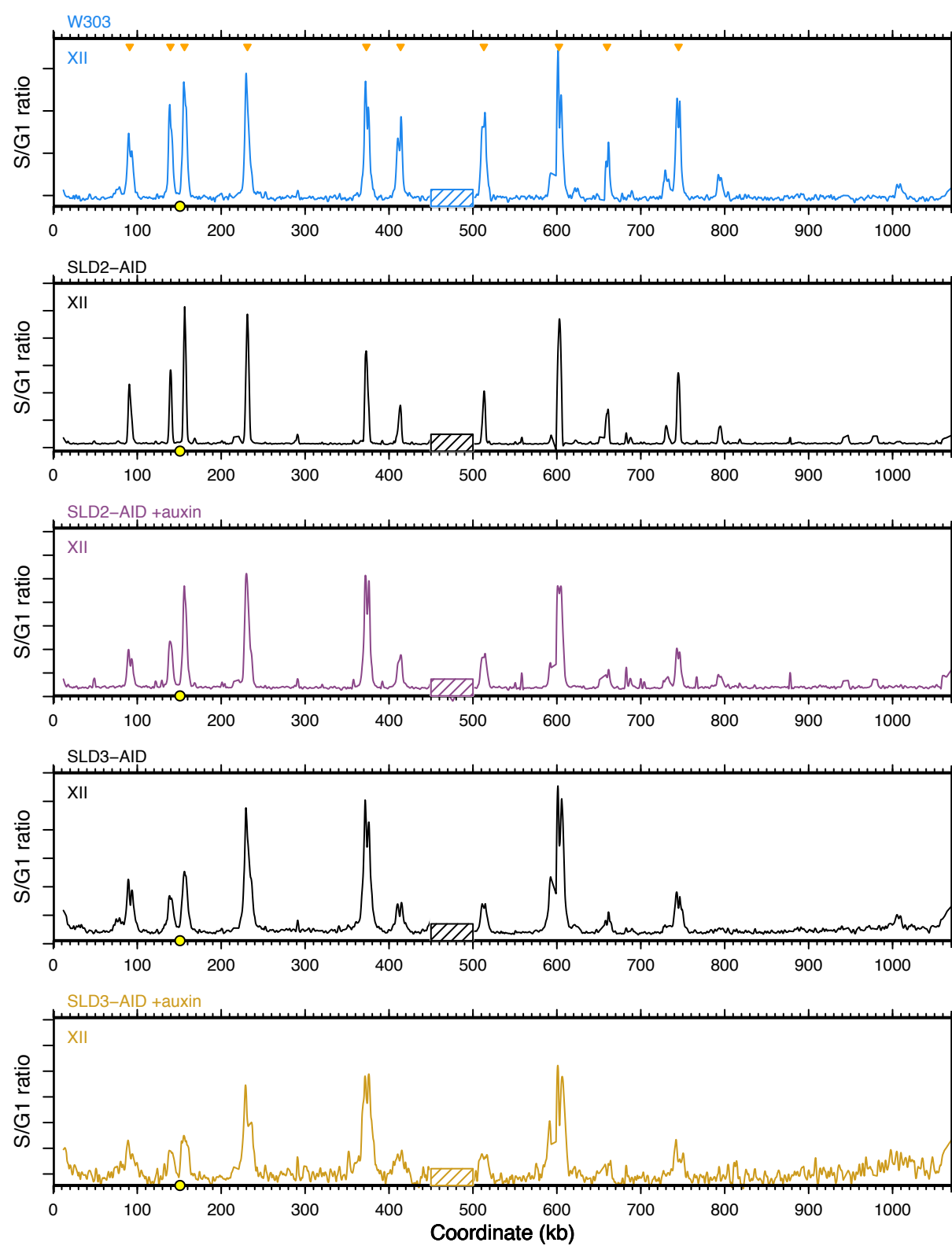

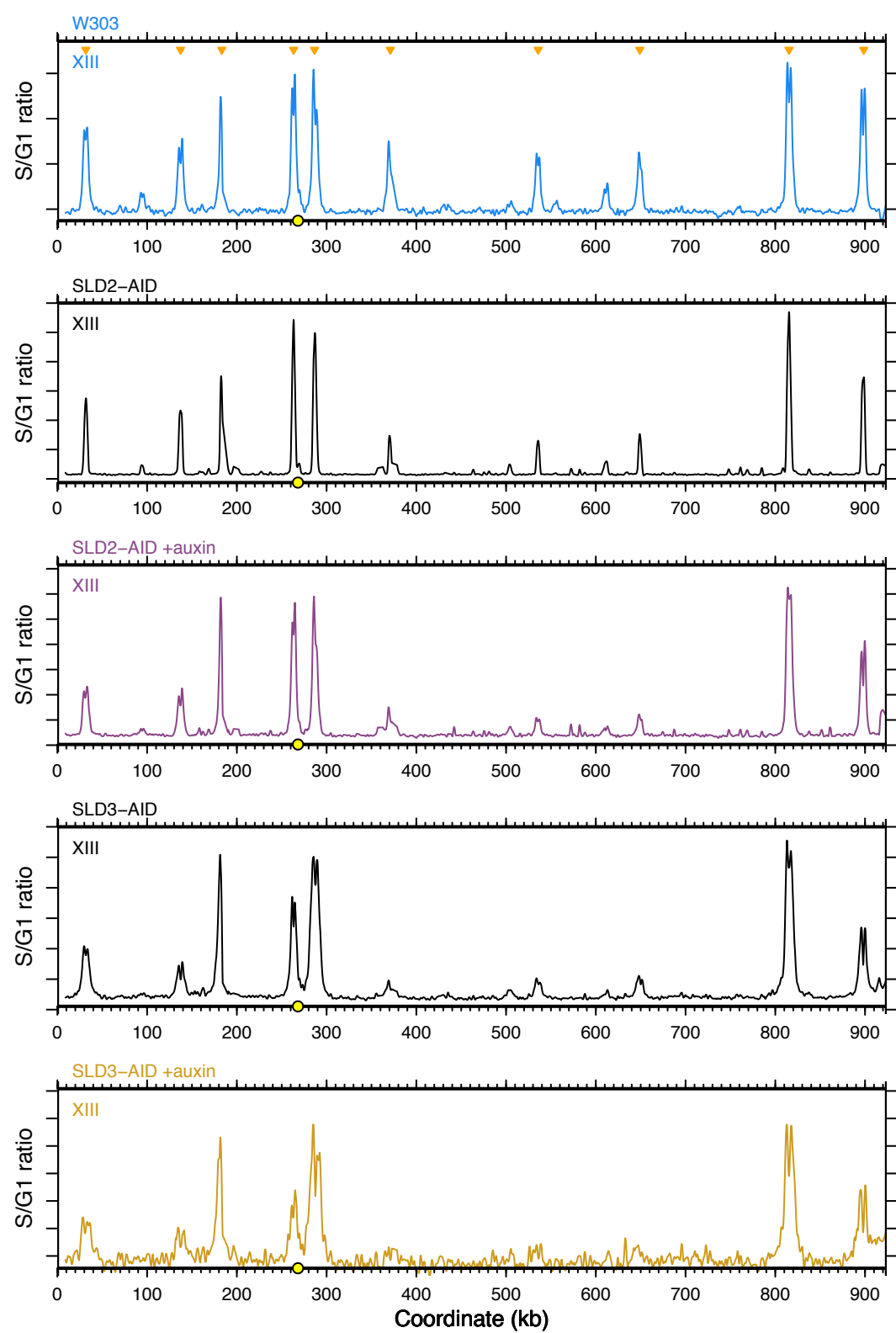

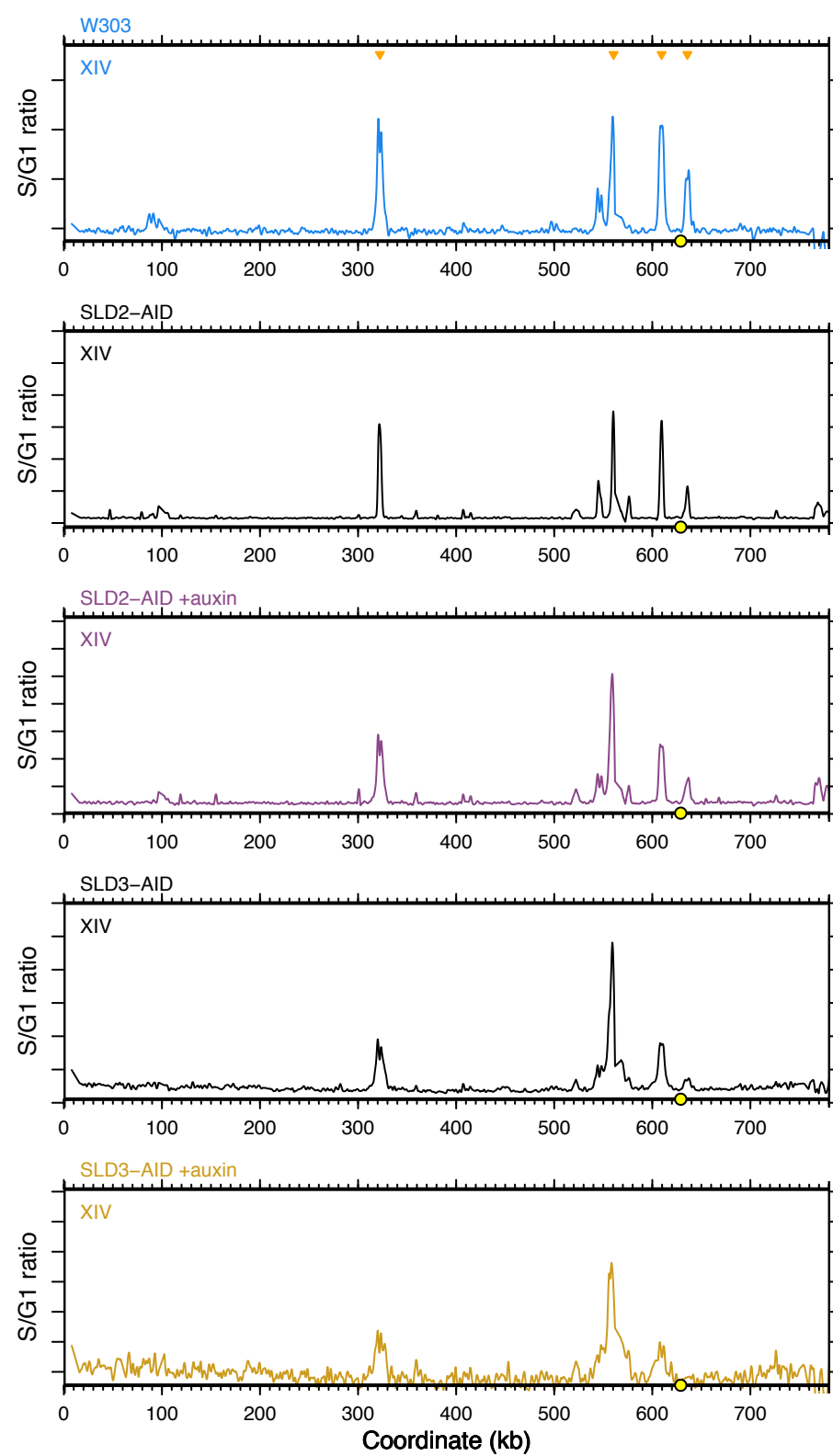

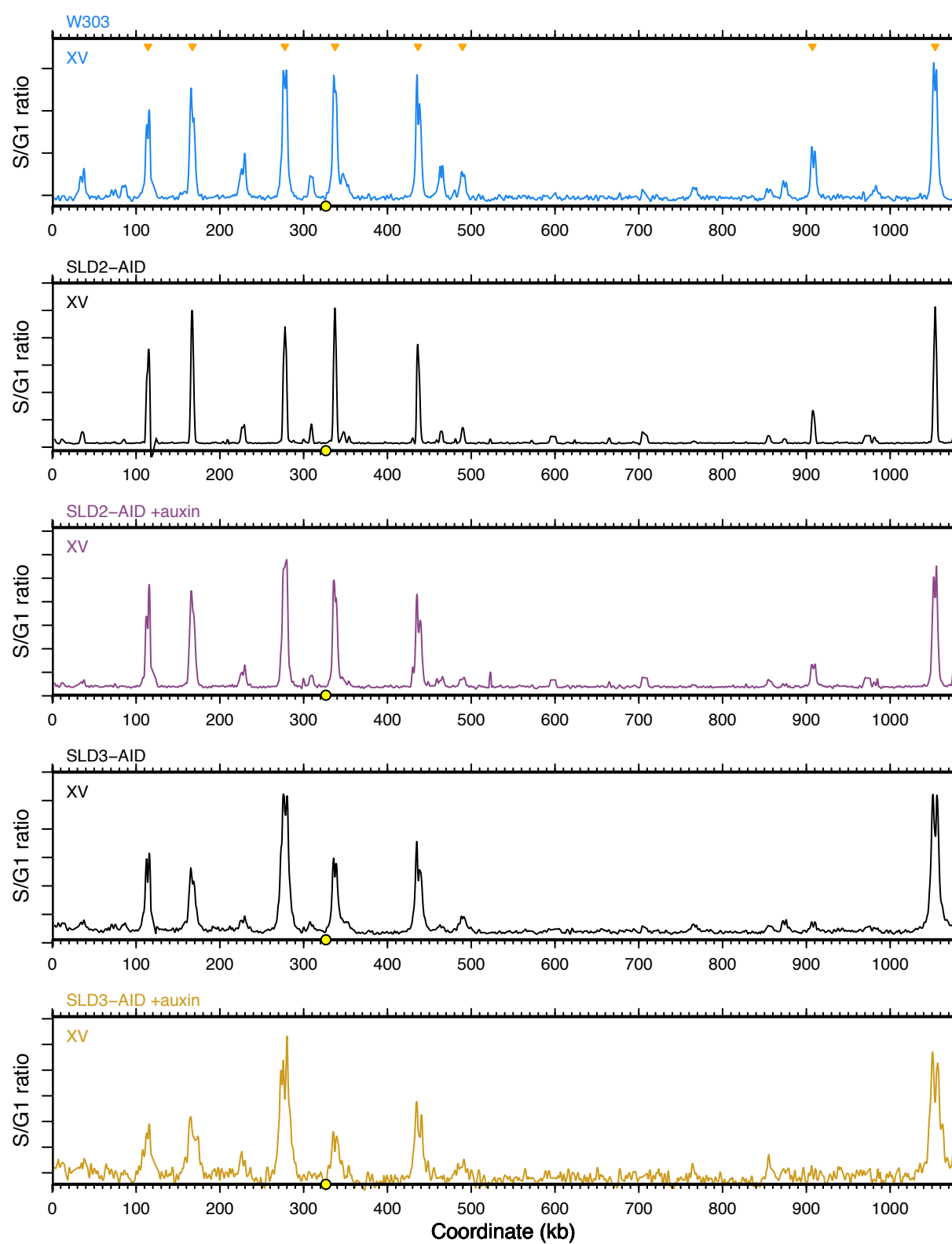

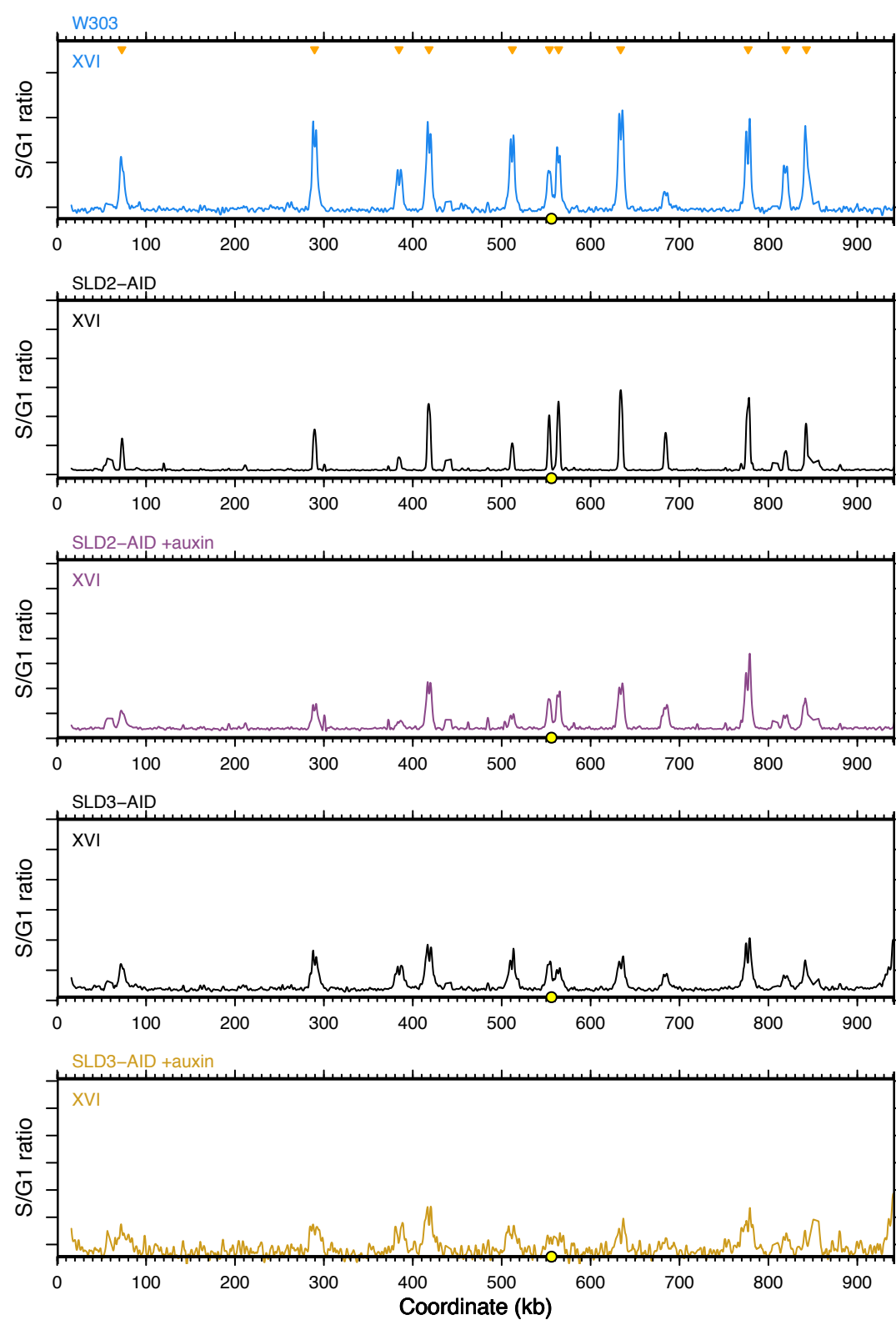

Supplement: S9 Fig — Orange triangle show origins called from WT. Chromosome coordinates are on the X axis and S/G1 ssDNA ratio is mapped on the Y axis. All samples were collected at 60 min. Values at the rDNA locus (striped box at coordinates 450–500 kb) are excluded because of low probe density on the microarrays. (PDF) [file pgen.1008430.s009.pdf]

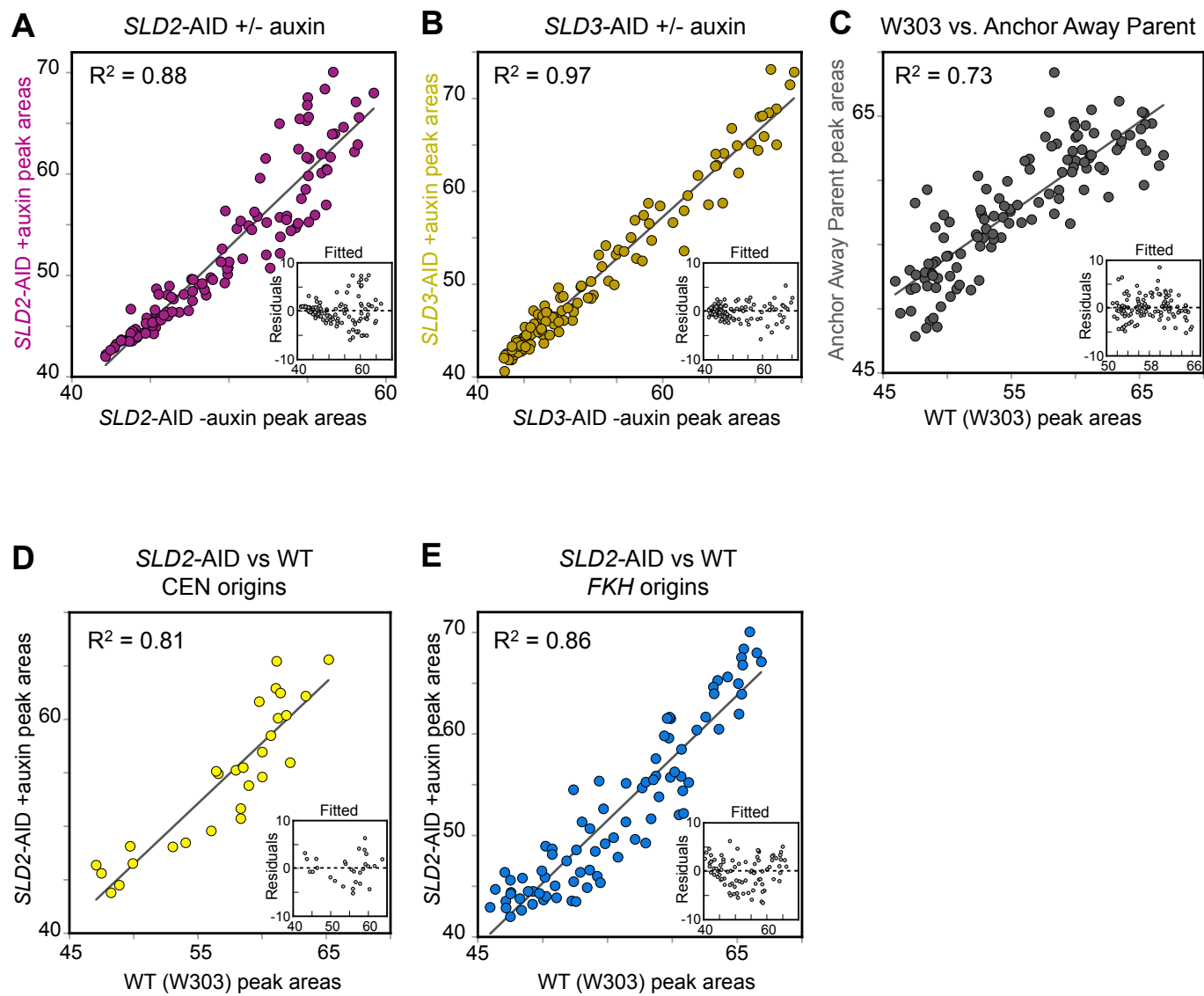

Supplement: S10 Fig — (A, B) Peak areas for the significant set of 117 origins in uninduced vs. induced SLD2-AID (A) and SLD3-AID (B) strains. Insets show residuals from linear fit models as a function of fitted values. (C) ssDNA peak area comparison of two untagged, untreated strains (W303 and Anchor Away Parent with untagged SLD2). Inset shows residuals from a linear fit model. (D) CEN-proximal origin peak area comparison for uninduced SLD2-AID compared to induced SLD2-AID. CEN-proximal origins are the subset of origins assayed by ssDNA assay within 50 kb of the centromere (28 origins). (E) FKH-regulated origin peak area comparison for uninduced SLD2-AID compared to induced SLD2-AID. FKH-regulated origins are the subset (84 origins) of origins detected by ssDNA replication profiling identified by Knott, et al. [27] as partially regulated by FKH1 and FKH2 transcription factor binding near origins. All samples were collected at 60 min. (PDF) [file pgen.1008430.s010.pdf]

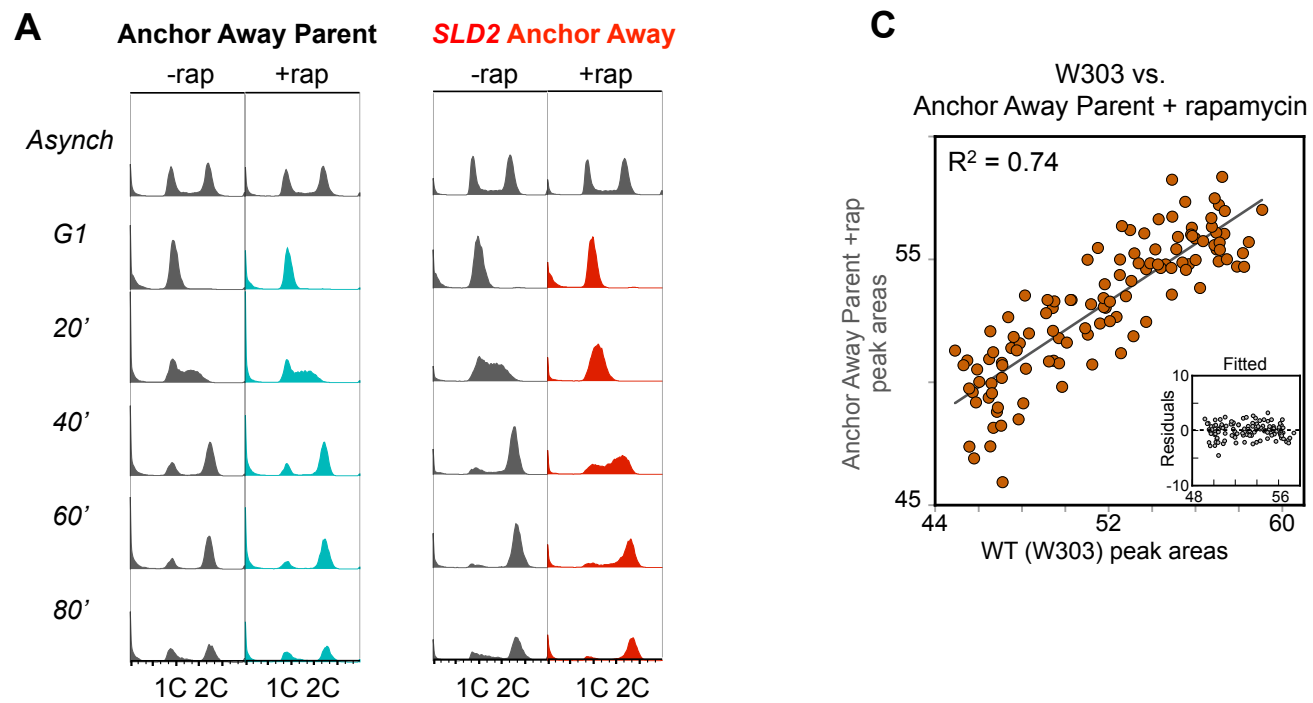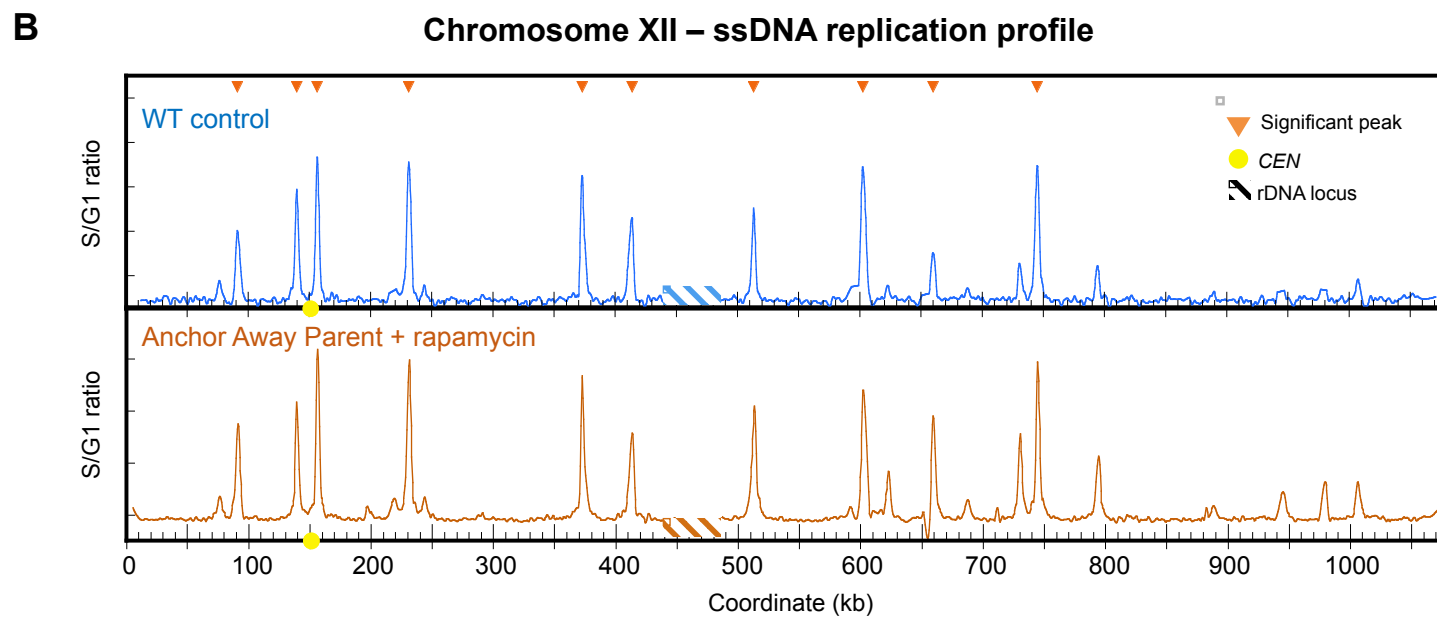

**D**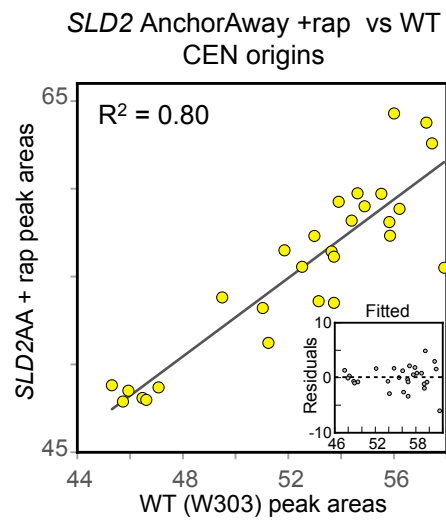**E**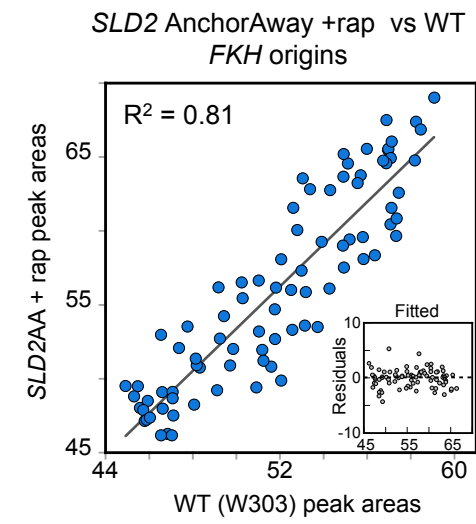

F

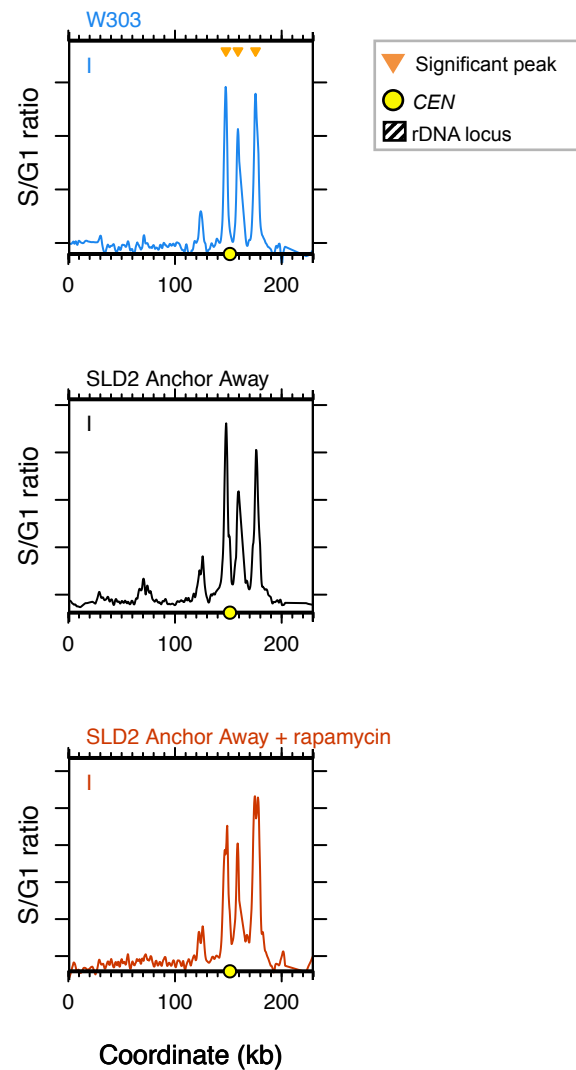

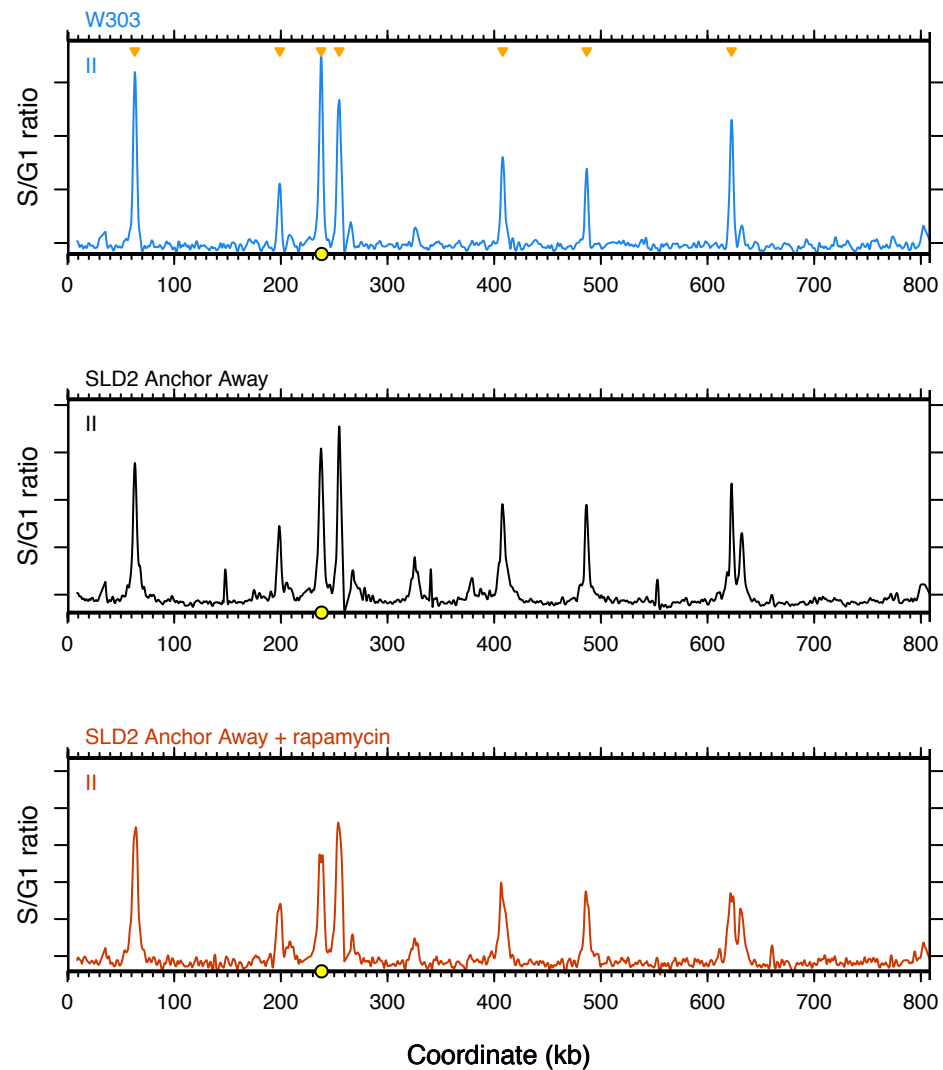

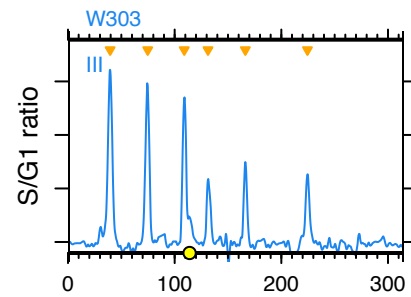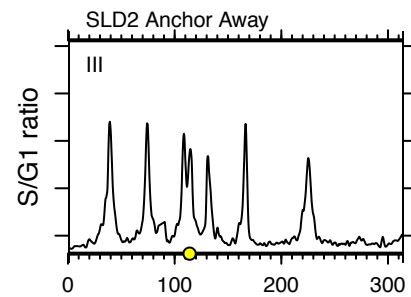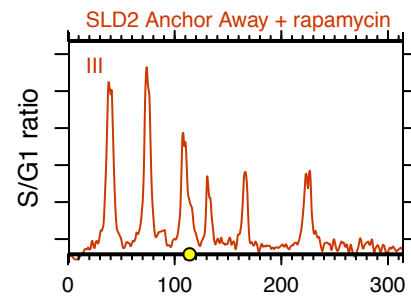

Coordinate (kb)

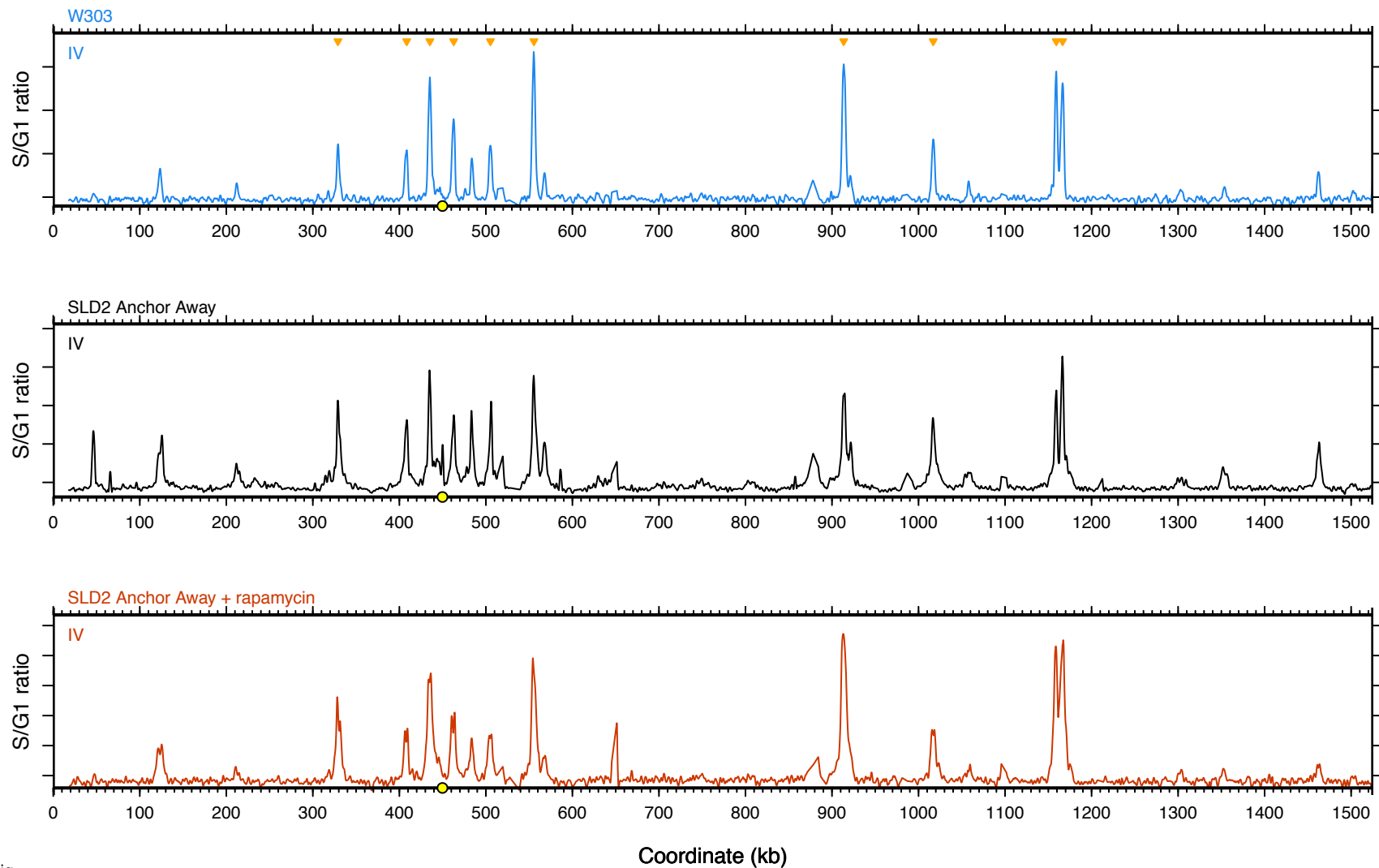

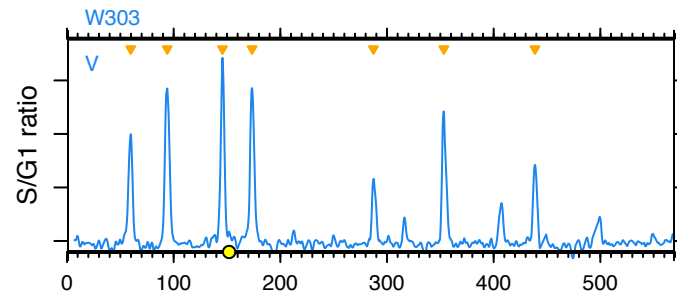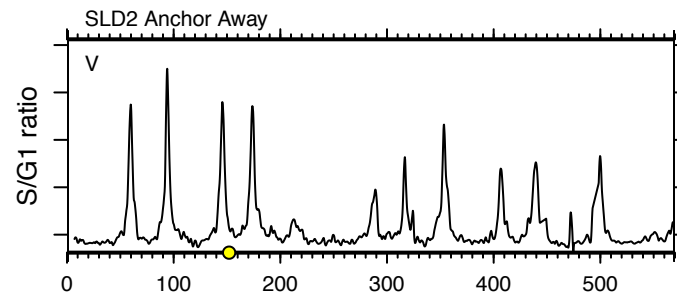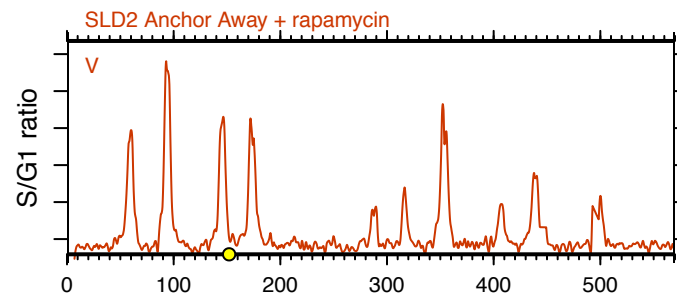

Coordinate (kb)

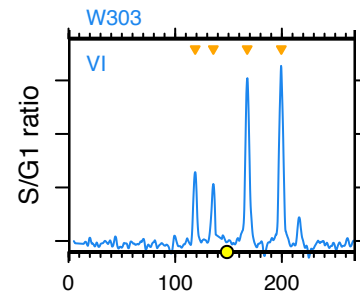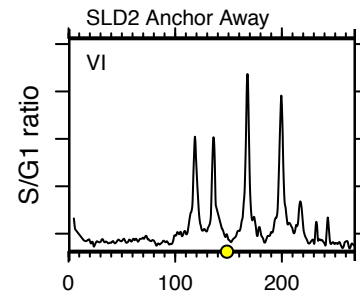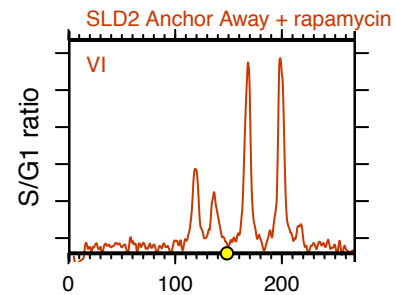

Coordinate (kb)

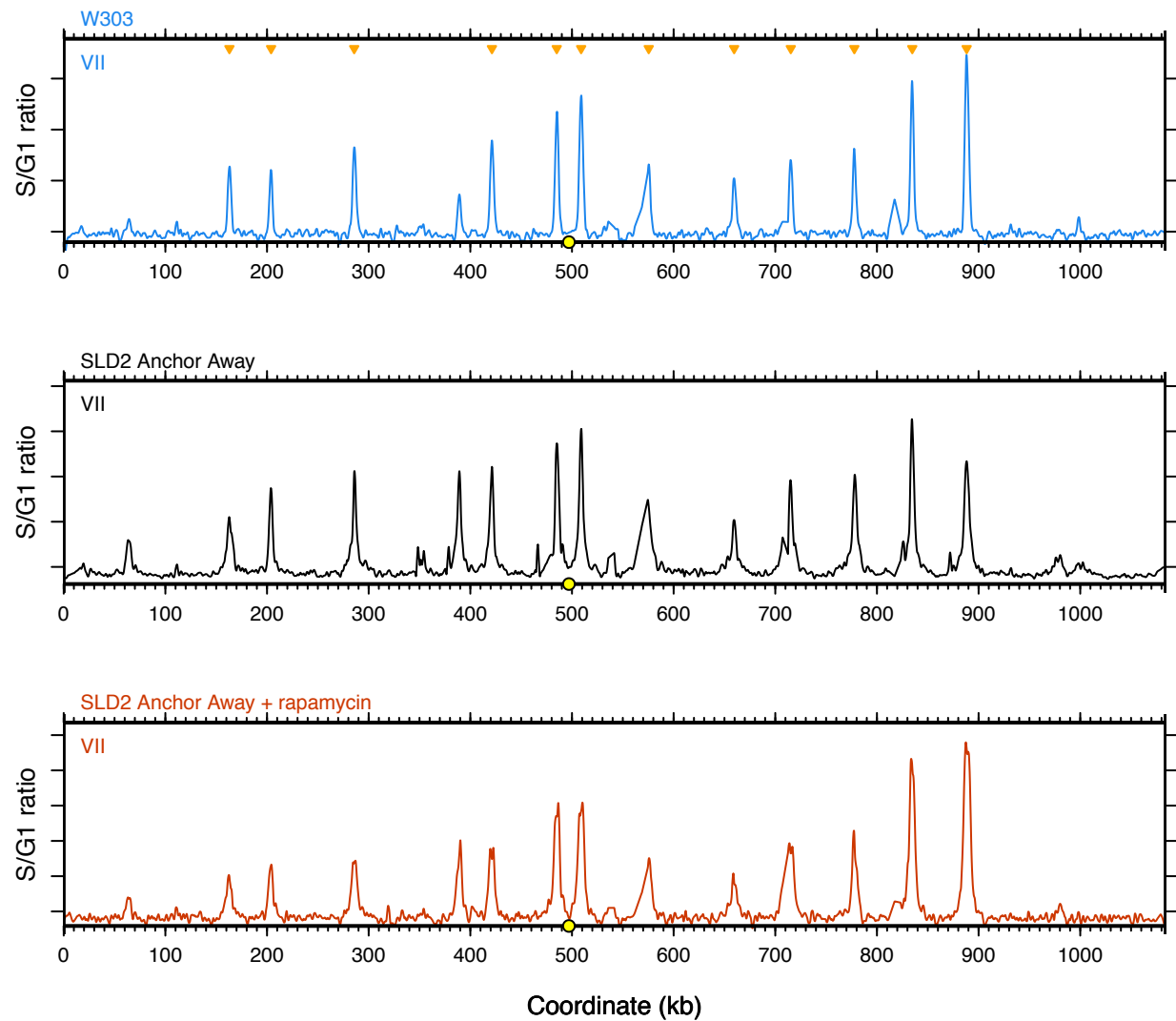

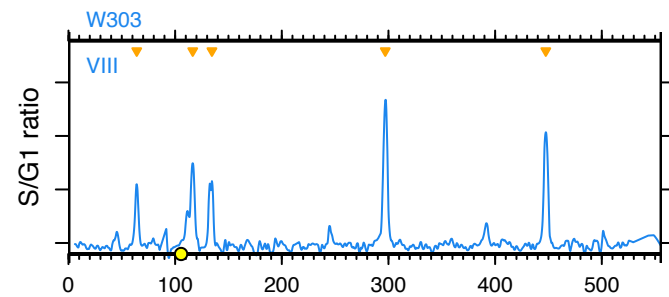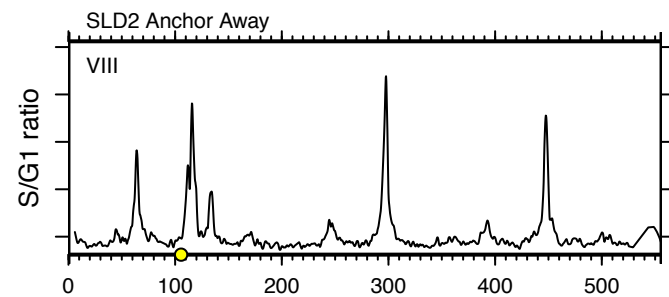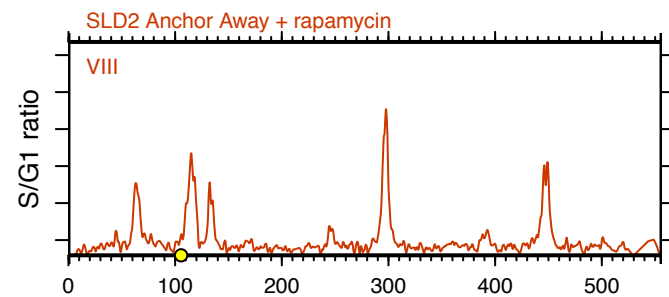

Coordinate (kb)

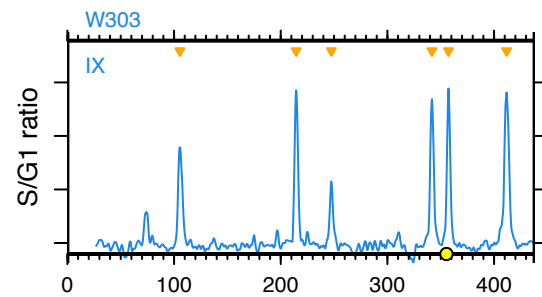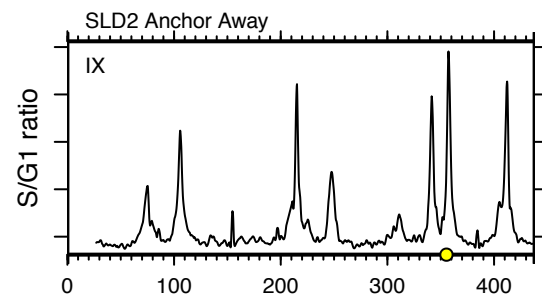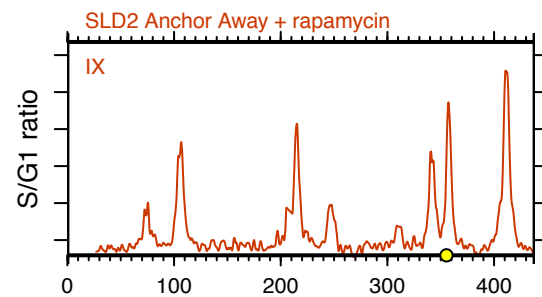

Coordinate (kb)

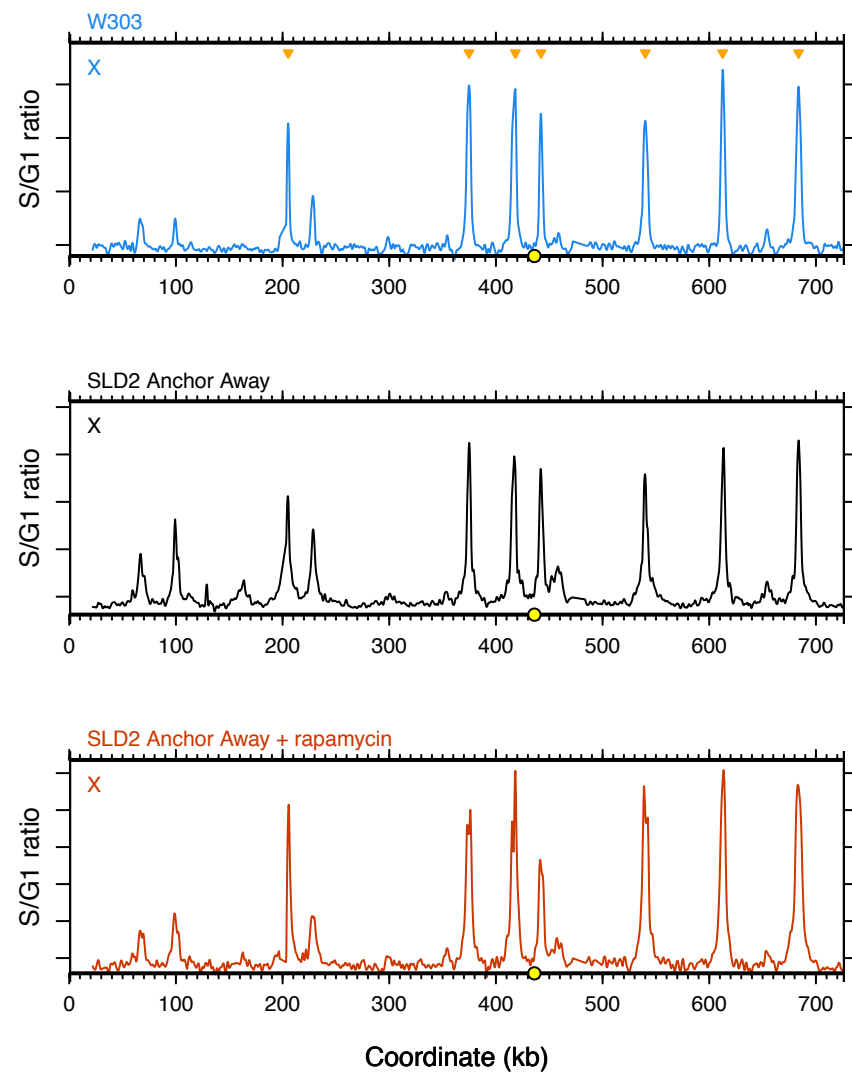

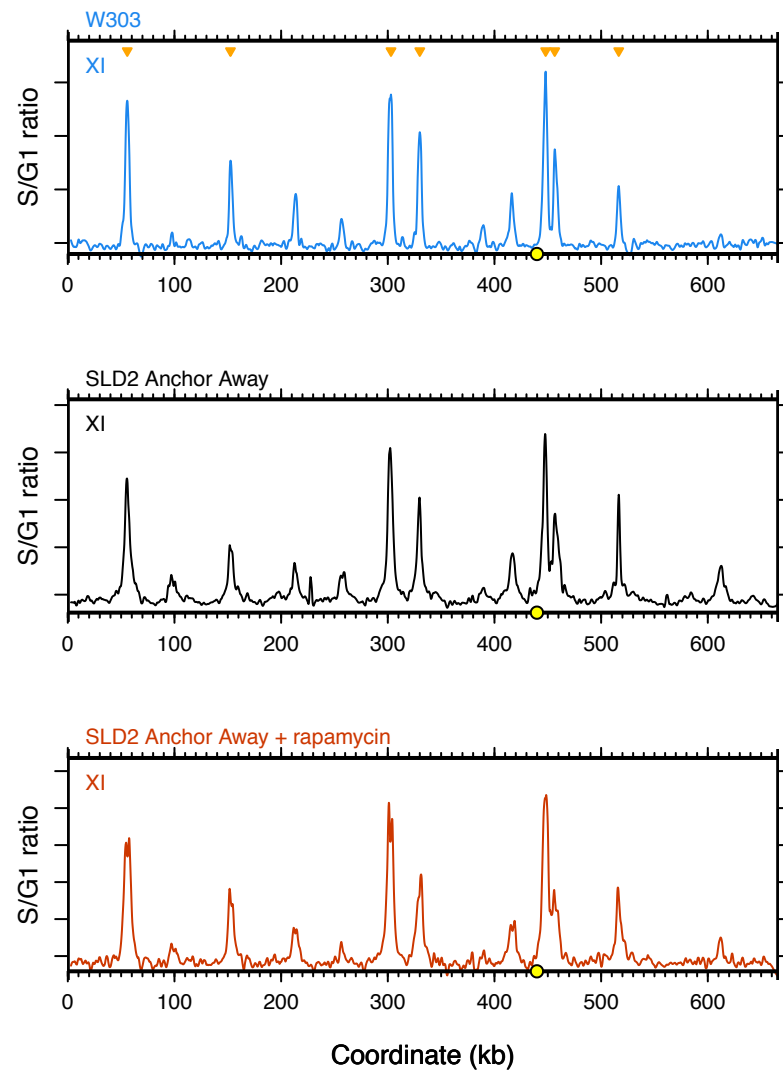

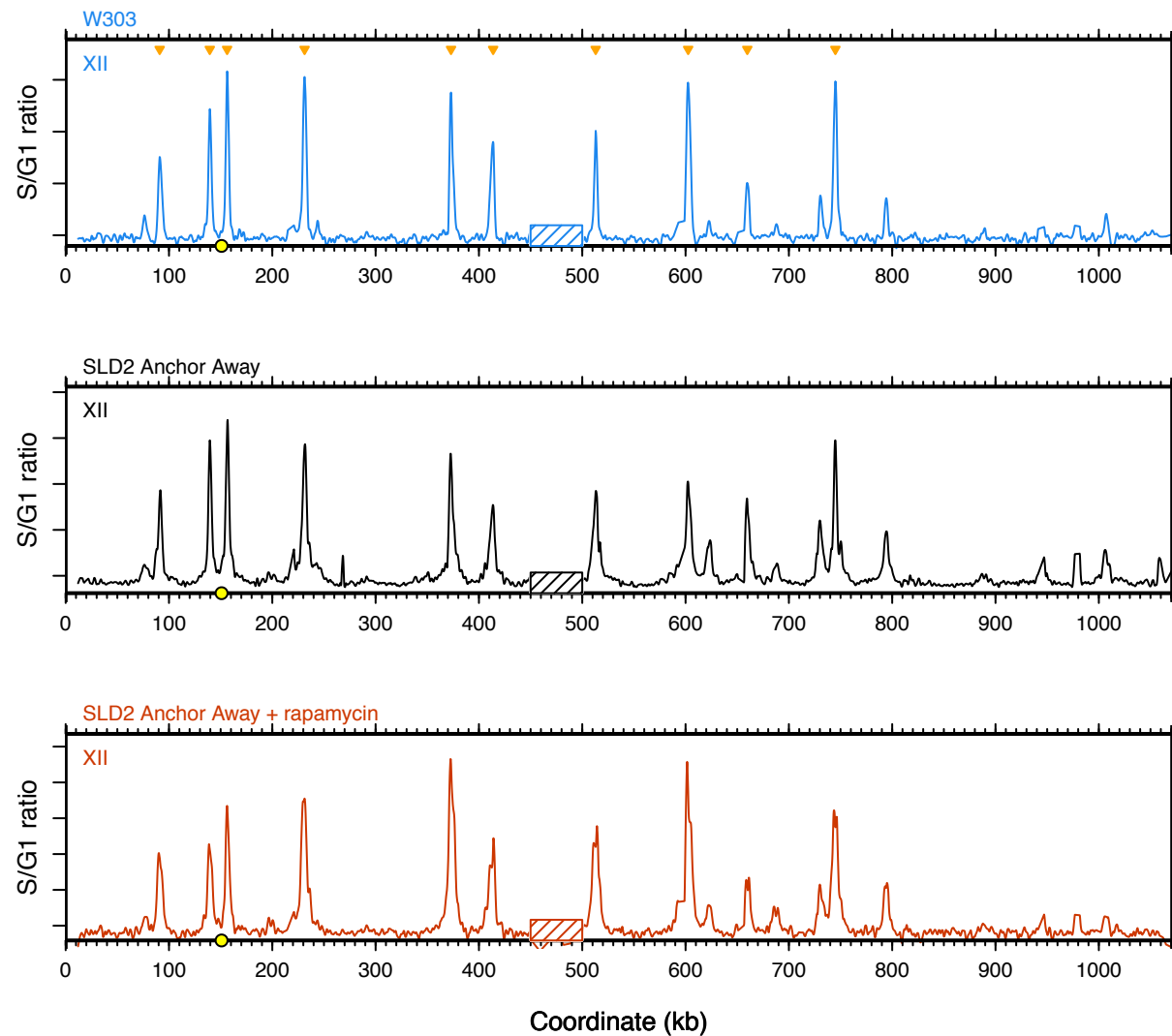

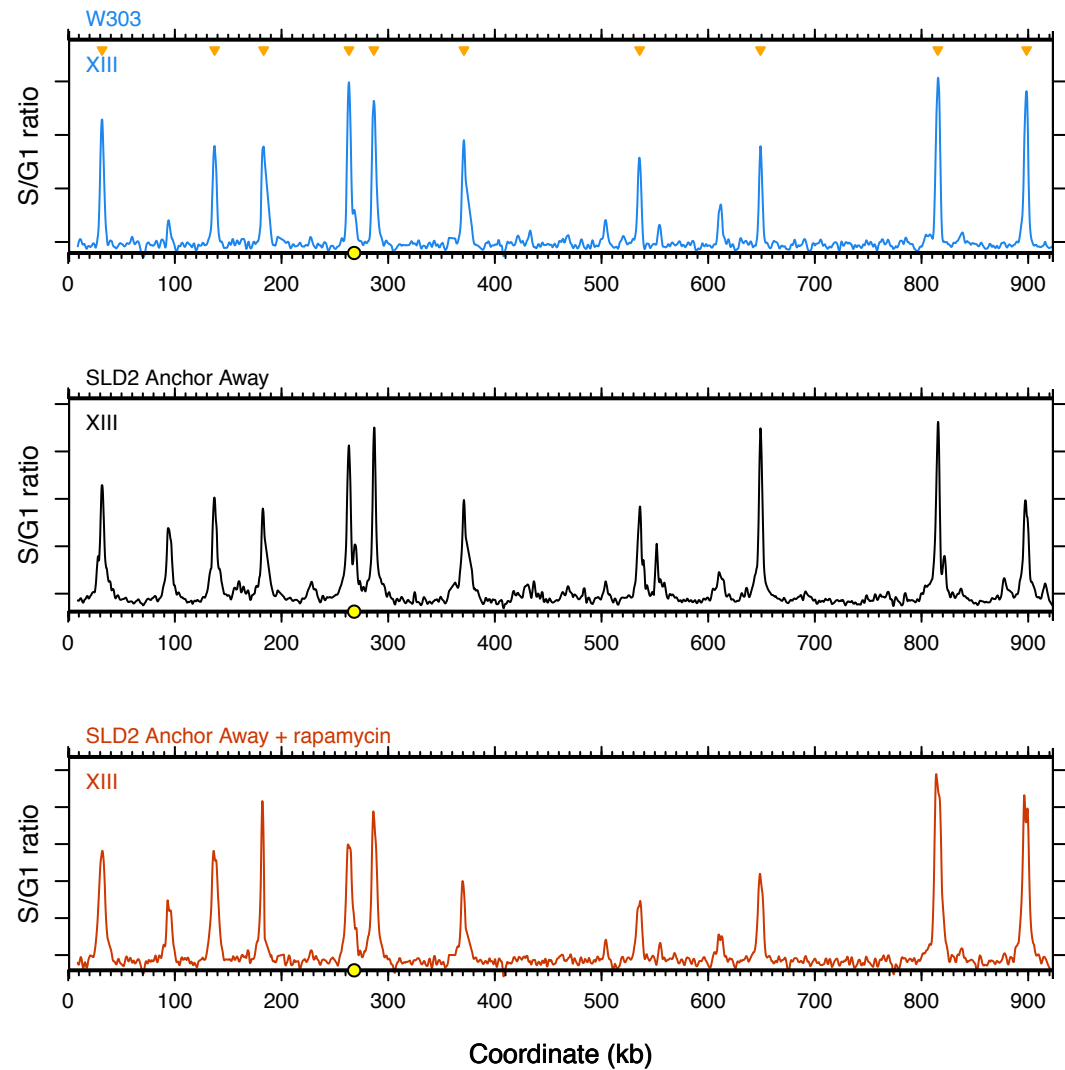

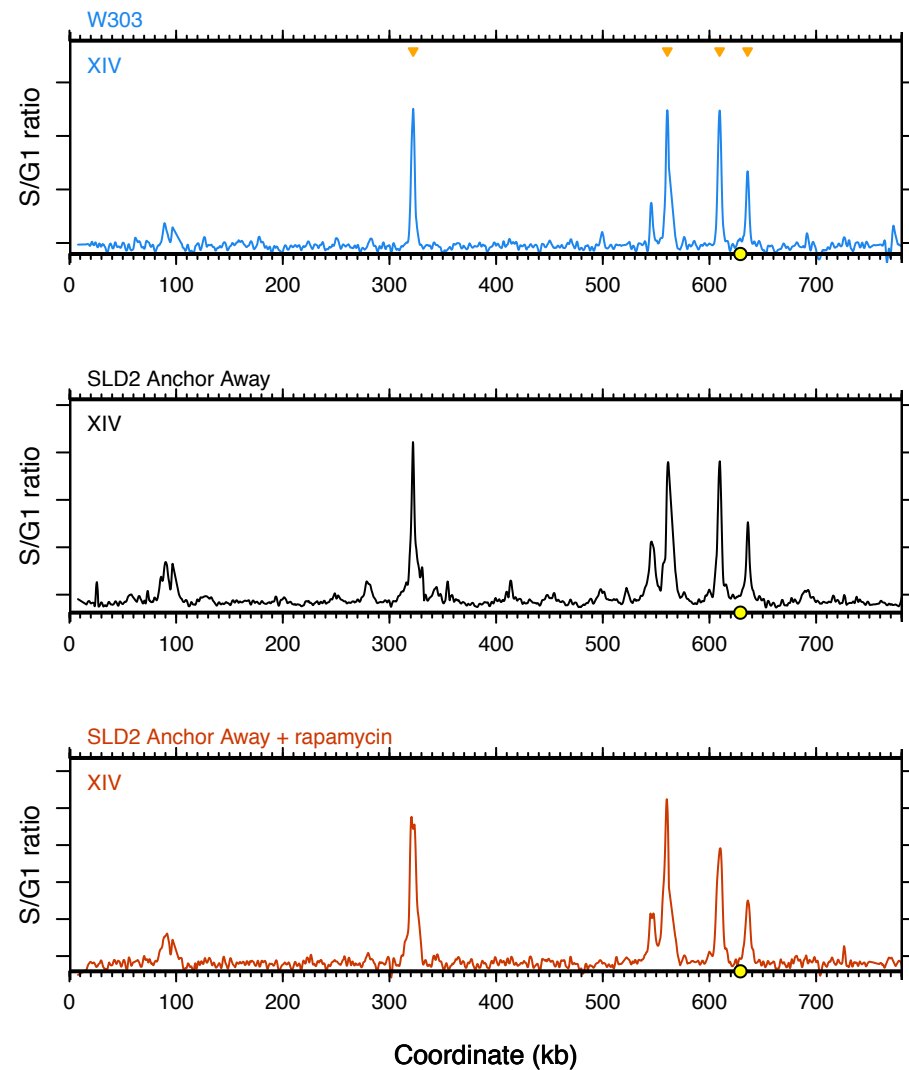

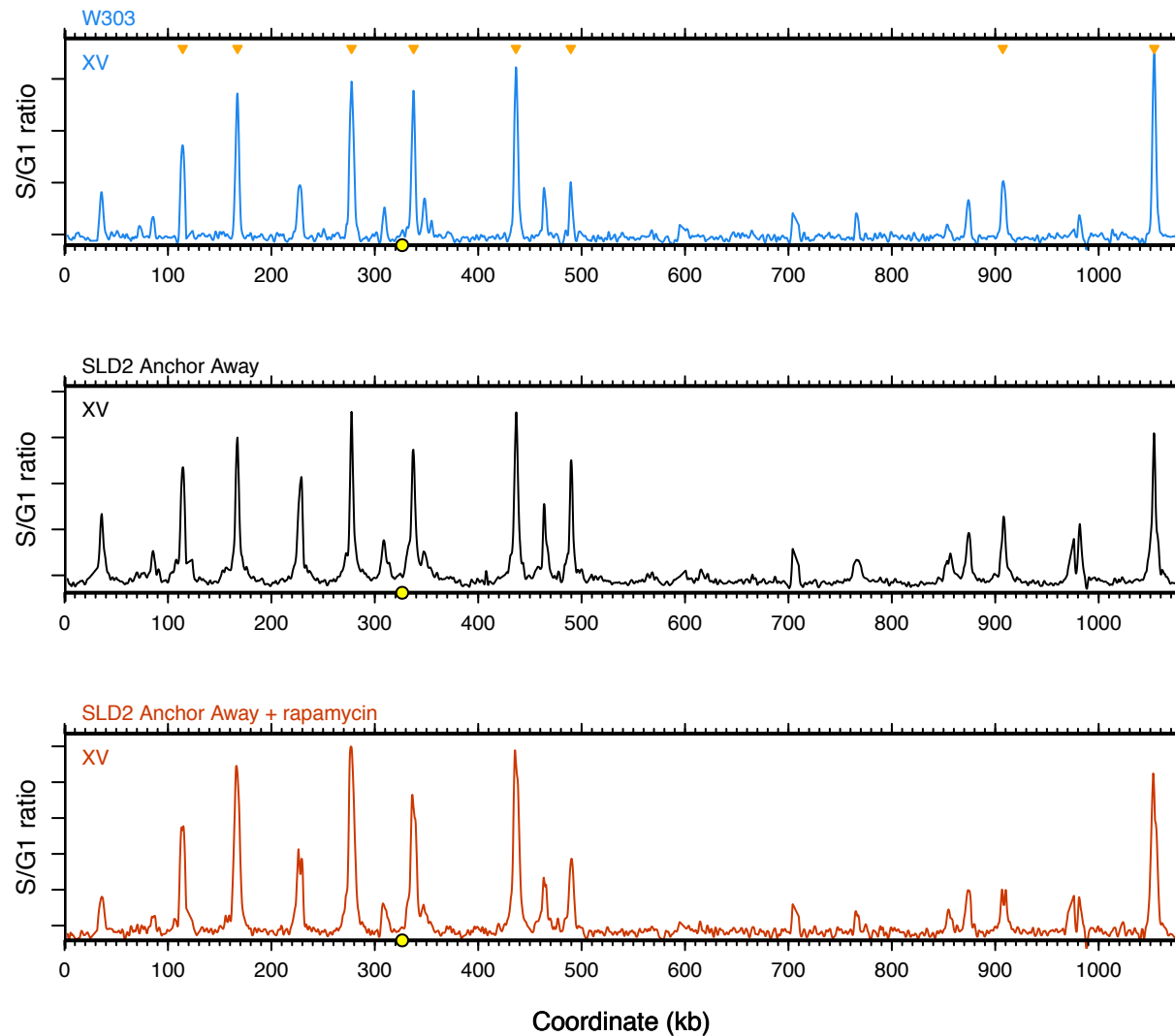

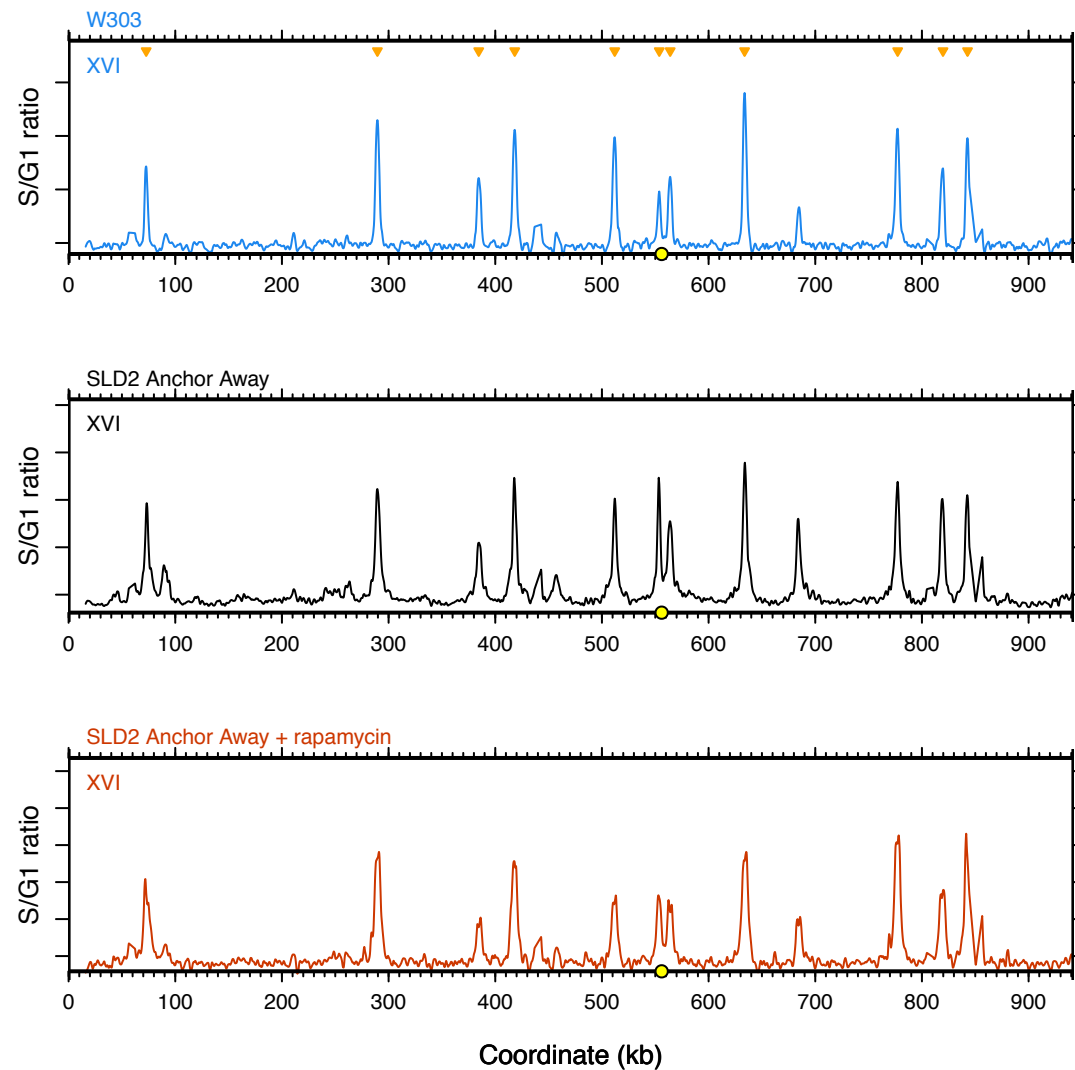

Supplement: S11 Fig — (A) S phase progression analysis by flow cytometry for Anchor Away parent and SLD2 Anchor Away cells with and without the addition of rapamycin. (B) ssDNA replication profile for chromosome XII in wild type control and rapamycin-treated Anchor Away parent strain. The wild type profile is the same as shown in Fig 6. (C) WT ssDNA peak areas compared to those seen in rapamycin-treated Anchor Away parent strain cells. (D) CEN-proximal origin peak area comparison for induced SLD2 Anchor Away compared to WT (28 origins). (E) FKH-regulated origin peak area comparison for uninduced SLD2 Anchor Away compared to induced SLD2 Anchor Away (84 origins). (F) SLD2 Anchor Away ssDNA replication profiles for all chromosomes. All samples were collected at 30 min. WT profiles are the same as in S8 Fig. (PDF) [file pgen.1008430.s011.pdf]
