# Supplementary material for: The effects of manipulating levels of replication initiation factors on origin firing efficiency in yeast
Source: PLoS Genet. 2019 Oct 4;15(10):e1008430. doi: 10.1371/journal.pgen.1008430 (PMC6795477; doi:10.1371/journal.pgen.1008430)
Supplement: S2 Fig — (A) CHEF gel quantification to determine the percentage of signal in the well of each lane. The hybridization signal from a section above the well was subtracted from the well signal and the lane signal portion of each sample to correct for background. The corrected well hybridization was then divided by the total hybridization signal for each individual sample to determine the “percent well signal” shown in the bar charts in (B)–(I). (B) Quantification of Chr IV SLD2-AID and SLD3-AID blots. The gray shading highlights the regions of maximum discrepancy between the uninduced and induced samples. (C) Chr IV-probed CHEF gel blots and quantification for the AID parent strain. (D) Quantification of SLD2-AID and SLD3-AID Chr XII CHEF gel blots. The gray shading highlights the regions of maximum discrepancy between the uninduced and induced condition. (E) Chr XII-probed blots and quantification for the AID parent strain. (F) Chr XV-probed blots and blot quantification the AID strains. The probe is a single copy sequence at ~810 kb. (G) Chr X-probed (ARS1011) blots and quantification. (H) Chr III-probed (ARS306) blots and quantification. (I) Single copy mitochondrial DNA sequence (COX1) probed blots and quantification. Flow cytometry profiles for the AID parent control are shown in S1 Fig. (PDF) [file pgen.1008430.s002.pdf]

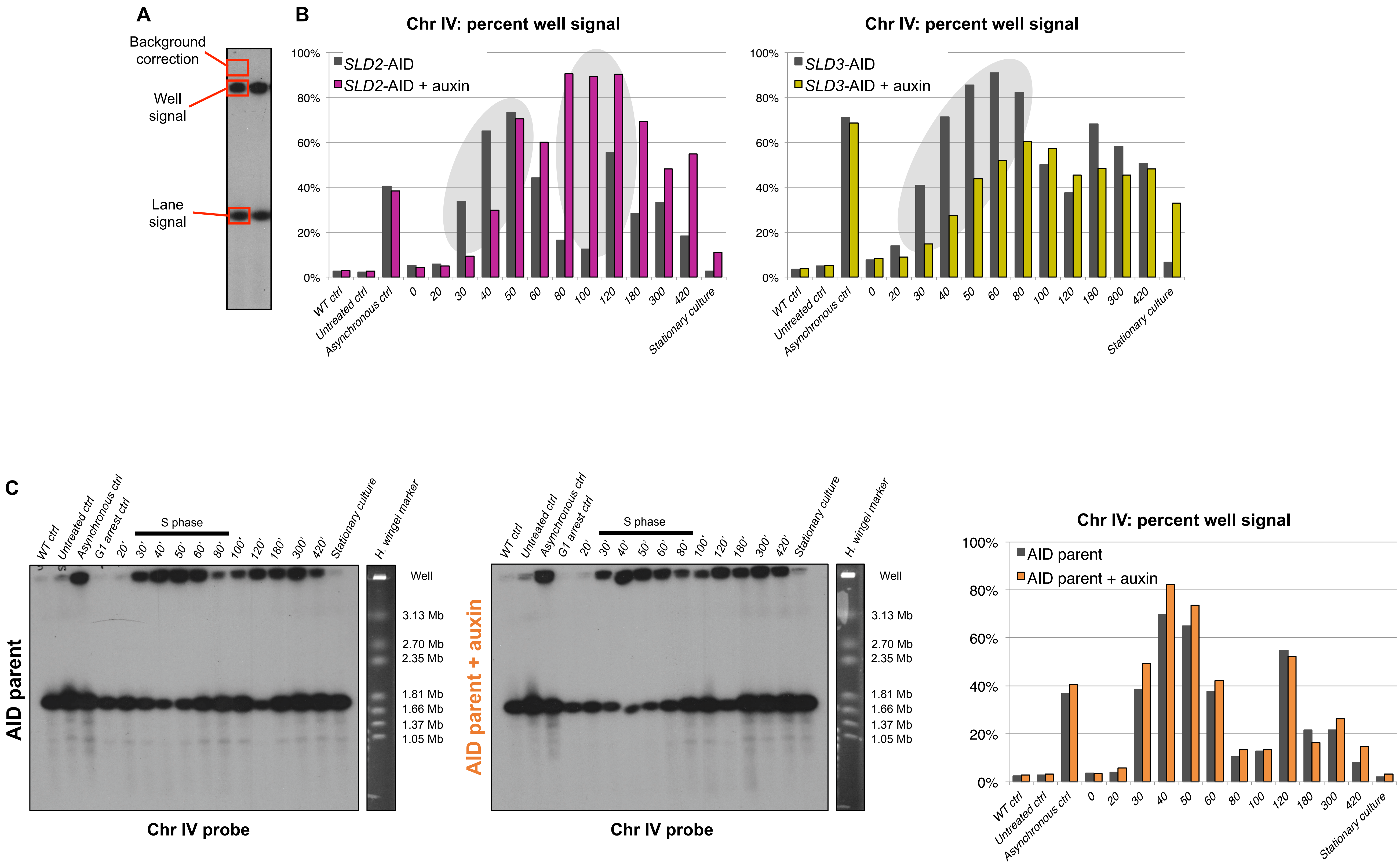
A
Background correction
Well signal
Lane signal
B
Chr IV: percent well signal
SLD2-AID
SLD2-AID + auxin
100%
80%
60%
40%
20%
0%
WT ctrl
Untreated ctrl
Asynchronous ctrl
0
20
30
40
50
60
80
100
120
180
300
420
Stationary culture
Chr IV: percent well signal
SLD3-AID
SLD3-AID + auxin
C
AID parent
WT ctrl
Untreated ctrl
Asynchronous ctrl
G1 arrest ctrl
20'
S phase
30'
40'
50'
60'
80'
100'
120'
180'
300'
420'
Stationary culture
H. wingei marker
Well
3.13 Mb
2.70 Mb
2.35 Mb
1.81 Mb
1.66 Mb
1.37 Mb
1.05 Mb
Chr IV probe
AID parent + auxin
Chr IV probe
Chr IV: percent well signal
AID parent
AID parent + auxin

S2_Fig_Lynch

D

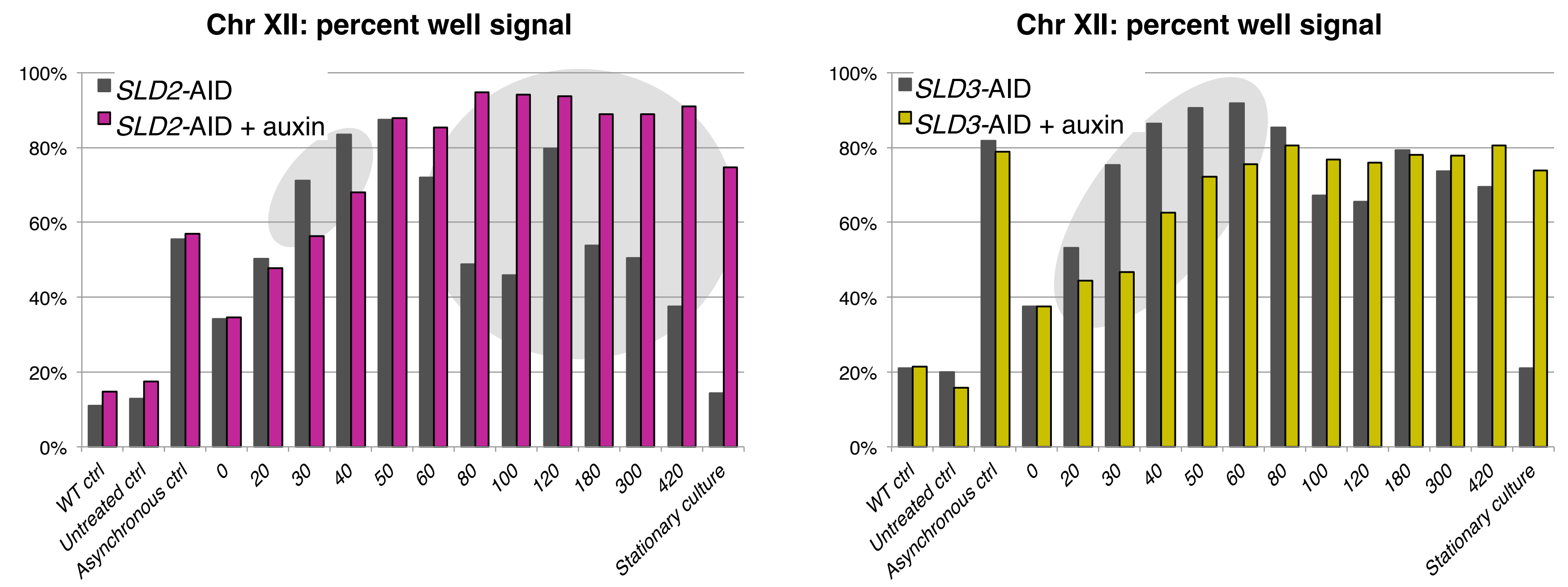

E

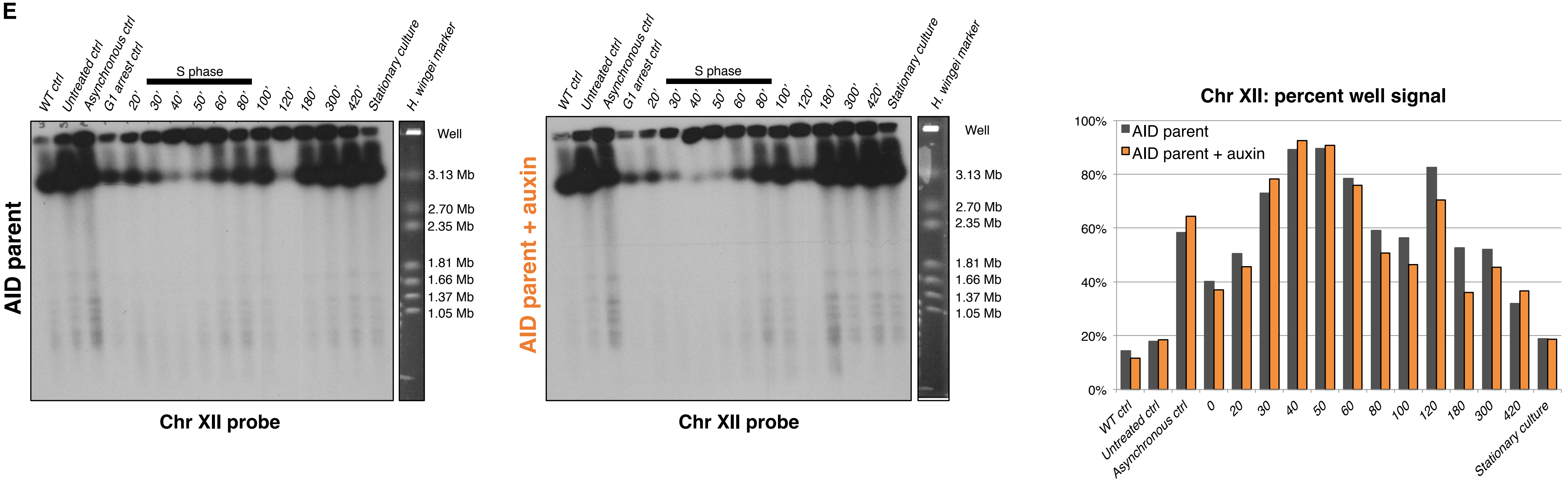

S2_Fig_Lynch

F

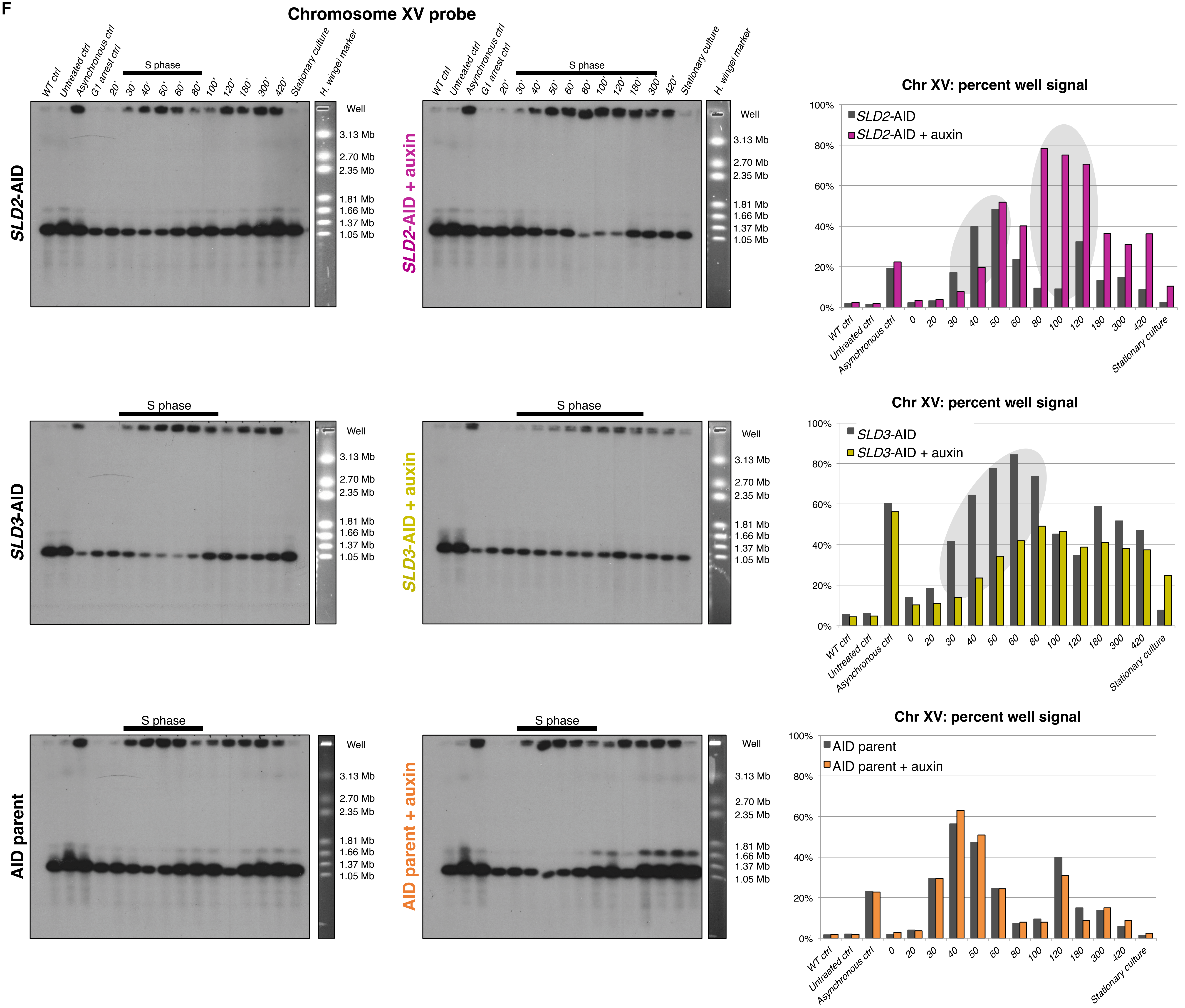

S2_Fig_Lynch

G

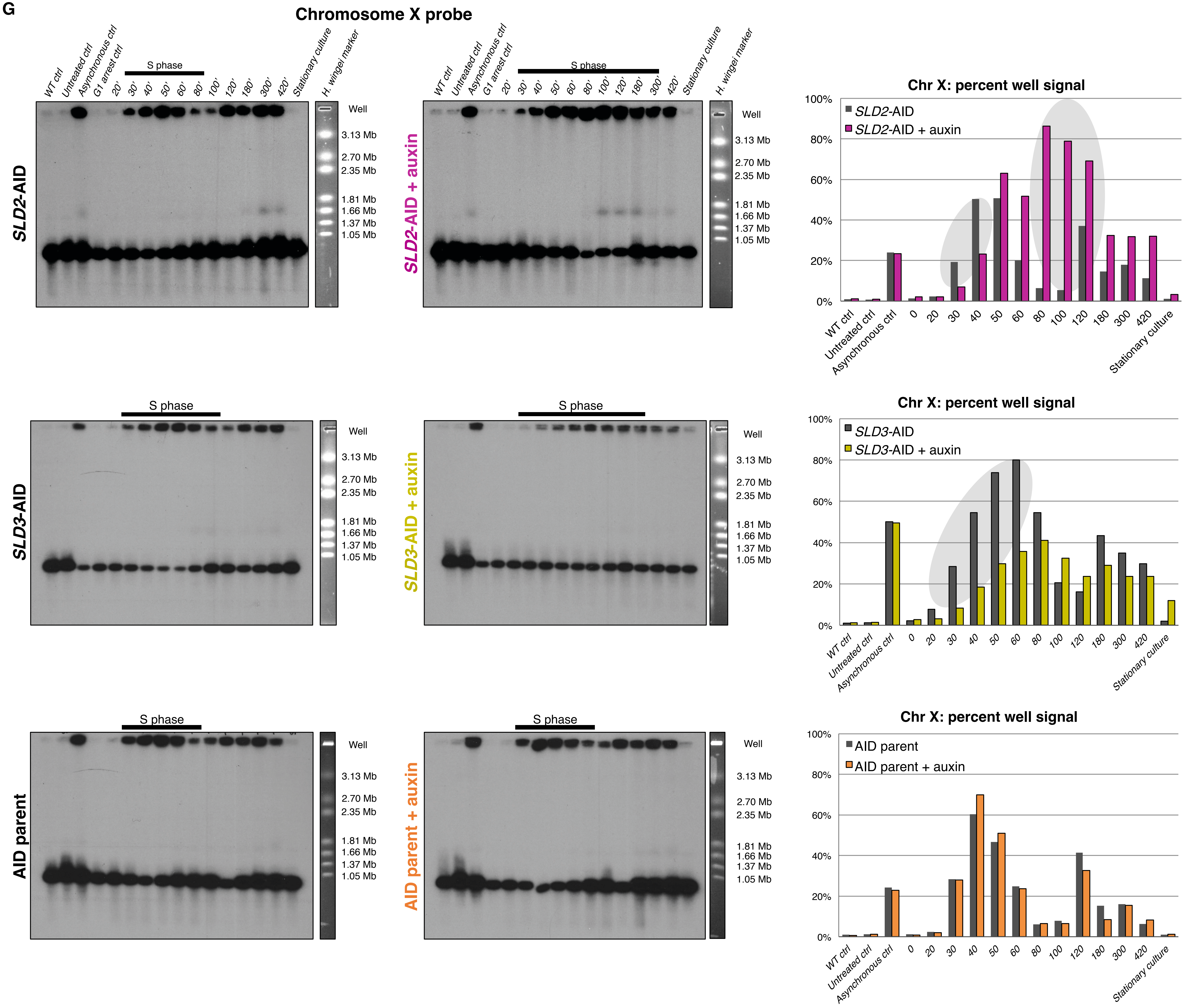

S2_Fig_Lynch

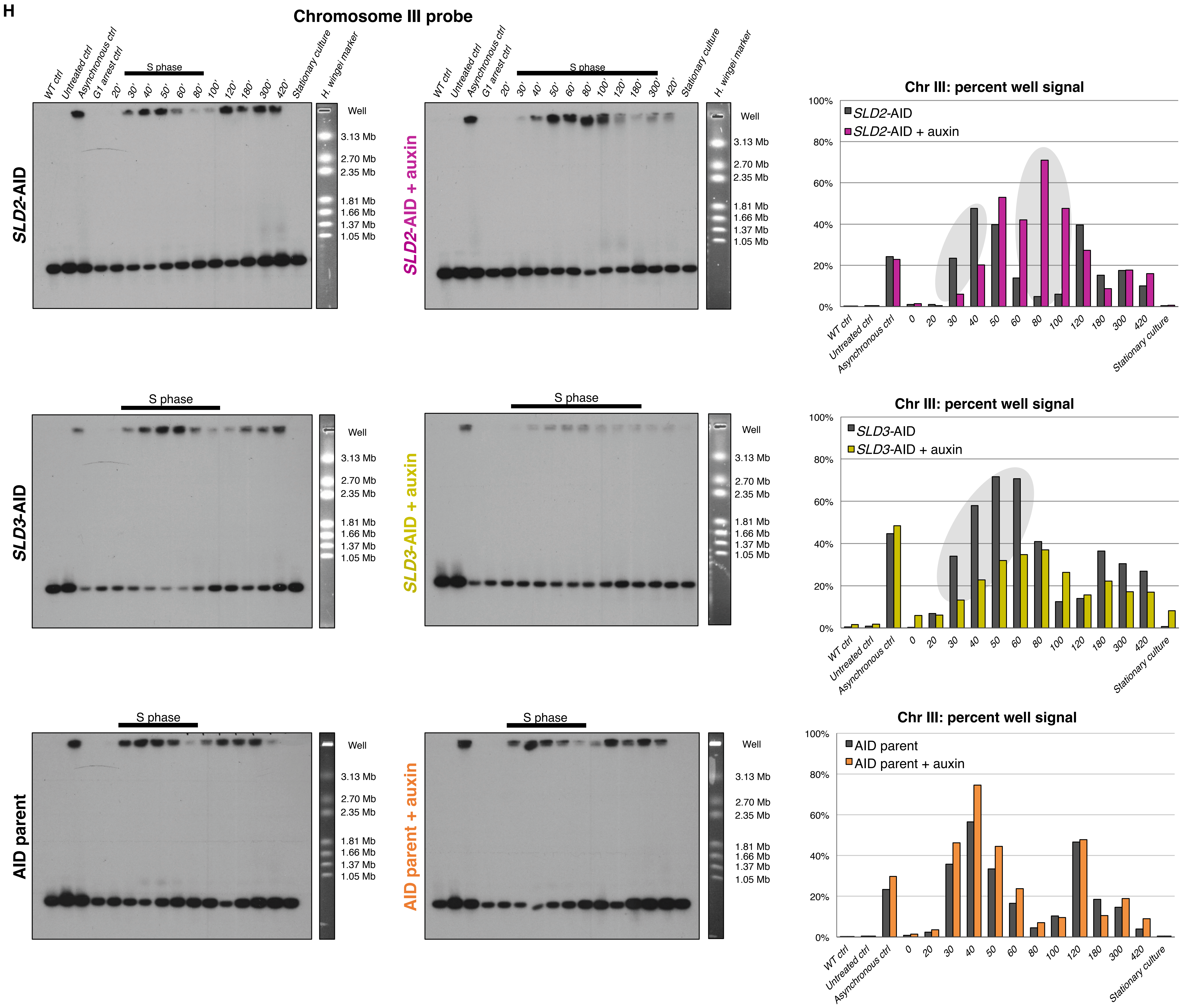
H
Chromosome III probe
WT ctrl
Untreated ctrl
Asynchronous ctrl
G1 arrest ctrl
S phase
20'
30'
40'
50'
60'
80'
100'
120'
180'
300'
420'
Stationary culture
H. wingei marker
Well
3.13 Mb
2.70 Mb
2.35 Mb
1.81 Mb
1.66 Mb
1.37 Mb
1.05 Mb
SLD2-AID
SLD2-AID + auxin
SLD3-AID
SLD3-AID + auxin
AID parent
AID parent + auxin
Chr III: percent well signal
SLD2-AID
SLD2-AID + auxin
SLD3-AID
SLD3-AID + auxin
AID parent
AID parent + auxin
100%
80%
60%
40%
20%
0%
WT ctrl
Untreated ctrl
Asynchronous ctrl
0
20
30
40
50
60
80
100
120
180
300
420
Stationary culture

S2_Fig_Lynch

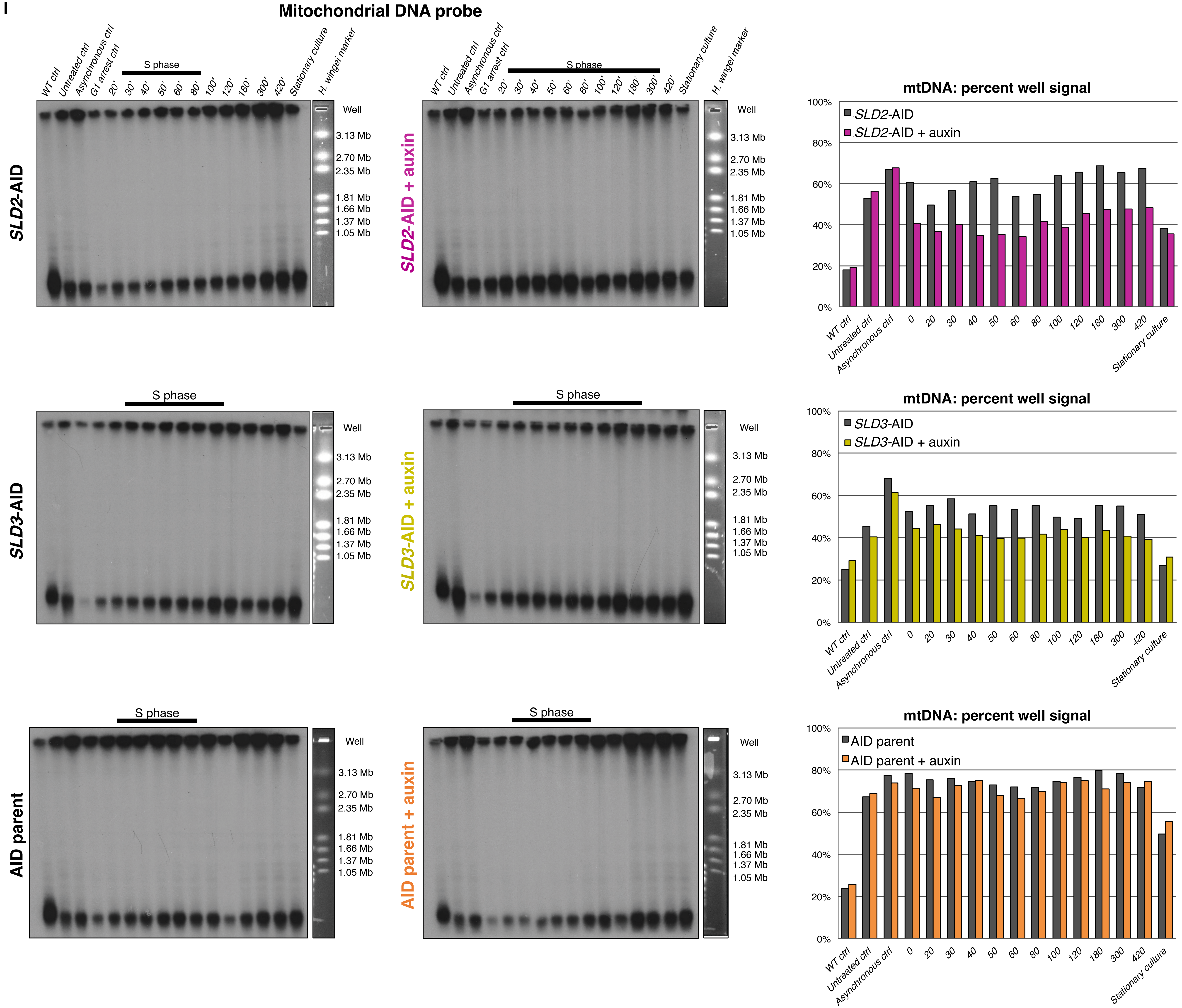
I
Mitochondrial DNA probe
WT ctrl
Untreated ctrl
Asynchronous ctrl
G1 arrest ctrl
S phase
20'
30'
40'
50'
60'
80'
100'
120'
180'
300'
420'
Stationary culture
H. wingei marker
Well
3.13 Mb
2.70 Mb
2.35 Mb
1.81 Mb
1.66 Mb
1.37 Mb
1.05 Mb
SLD2-AID
SLD2-AID + auxin
mtDNA: percent well signal
SLD2-AID
SLD2-AID + auxin
SLD3-AID
SLD3-AID + auxin
AID parent
AID parent + auxin
100%
80%
60%
40%
20%
0%
0
20
30
40
50
60
80
100
120
180
300
420

S2_Fig_Lynch
