# Supplementary material for: The effects of manipulating levels of replication initiation factors on origin firing efficiency in yeast
Source: PLoS Genet. 2019 Oct 4;15(10):e1008430. doi: 10.1371/journal.pgen.1008430 (PMC6795477; doi:10.1371/journal.pgen.1008430)
Supplement: S12 Fig — (A) We analyzed synchronous S phase progression by flow cytometry for SSDD and SSDDCS overexpression strains without HU. Times indicate the time after release from α-factor arrest. The blue line marks the position of cells with 1C DNA. (B) ssDNA replication profiles for wild type and overexpression strains of SSDD and SSDDCS collected at 120 min. See Fig 8 legend for details. Orange triangles show origins called from WT. Yellow circle marks the centromere. Chromosome coordinates are on the X axis and S/G1 ssDNA ratio is mapped on the Y axis. (PDF) [file pgen.1008430.s012.pdf]

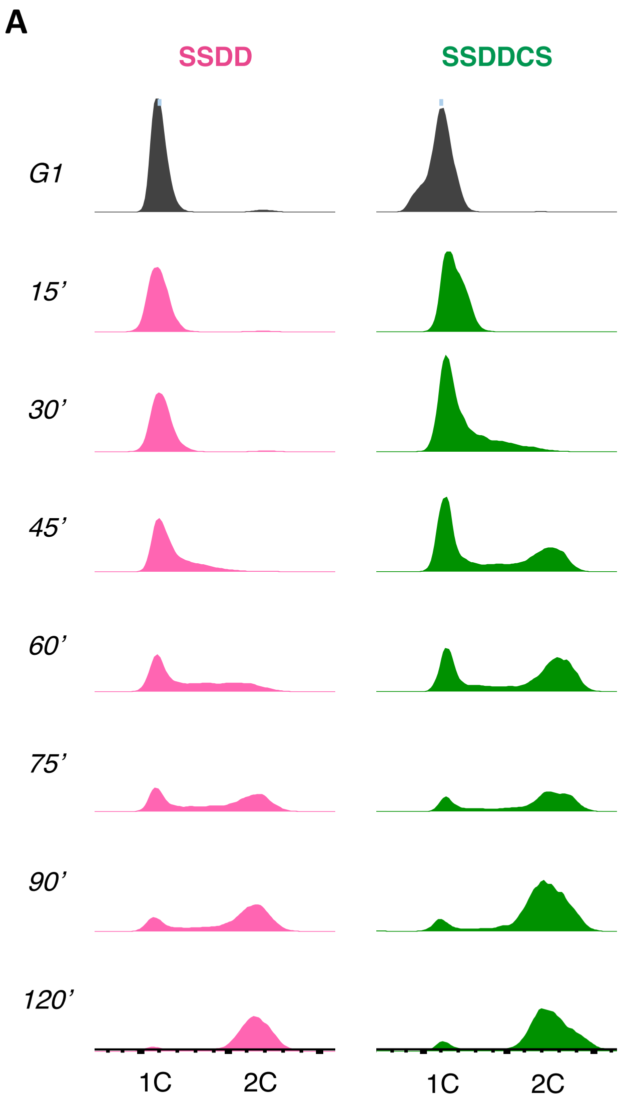

Lynch S12 Fig

B

WT, SSDD overexpression & SSDDCS overexpression ssDNA assay replication profiles

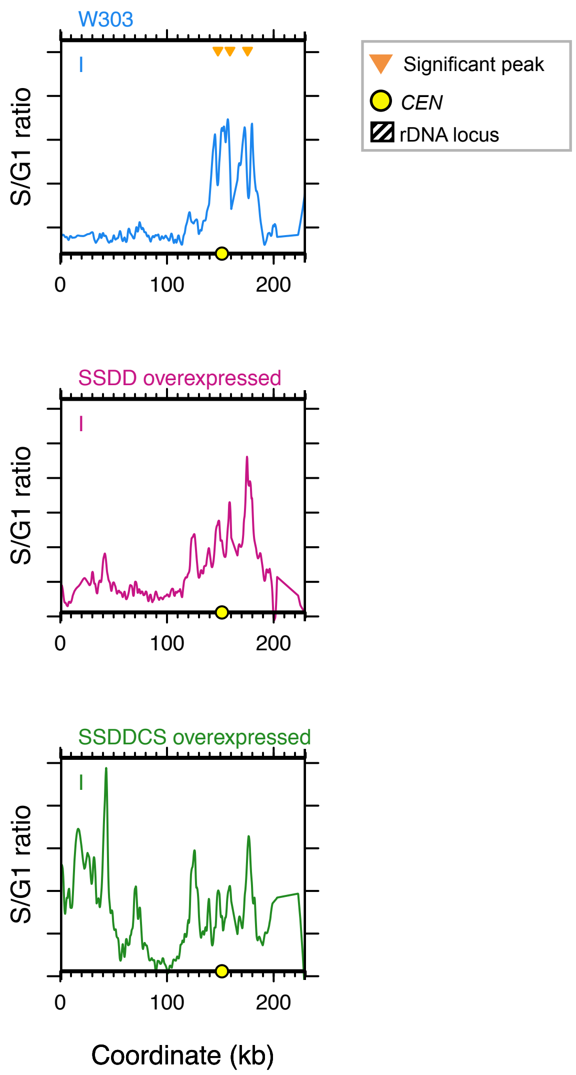

Lynch S12 Fig

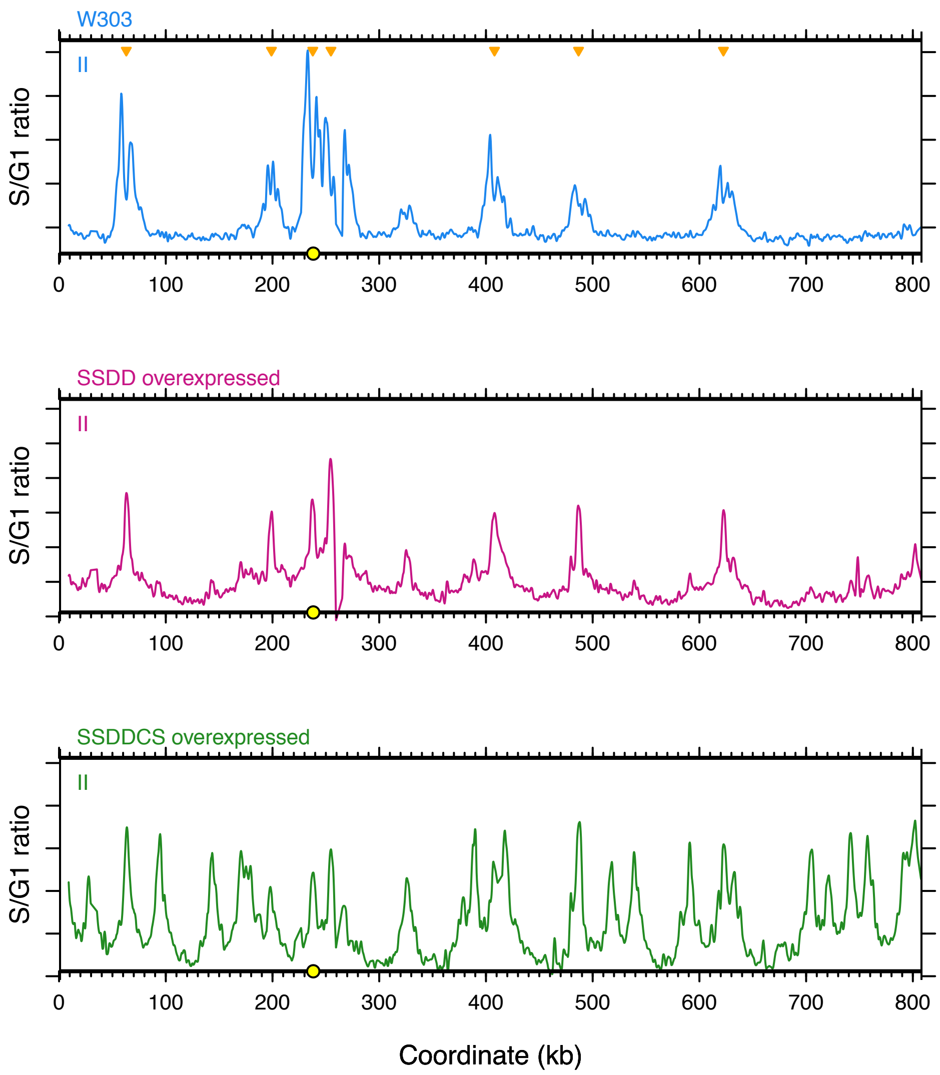

Lynch S12 Fig

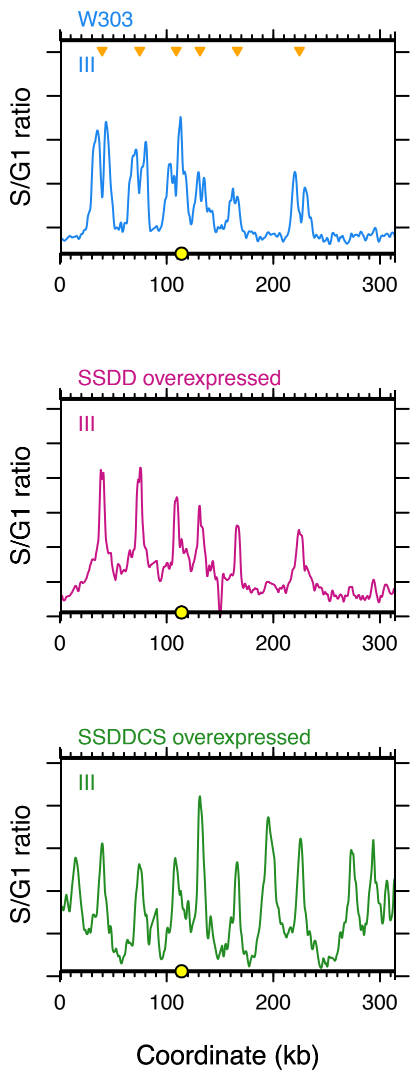

Lynch S12 Fig

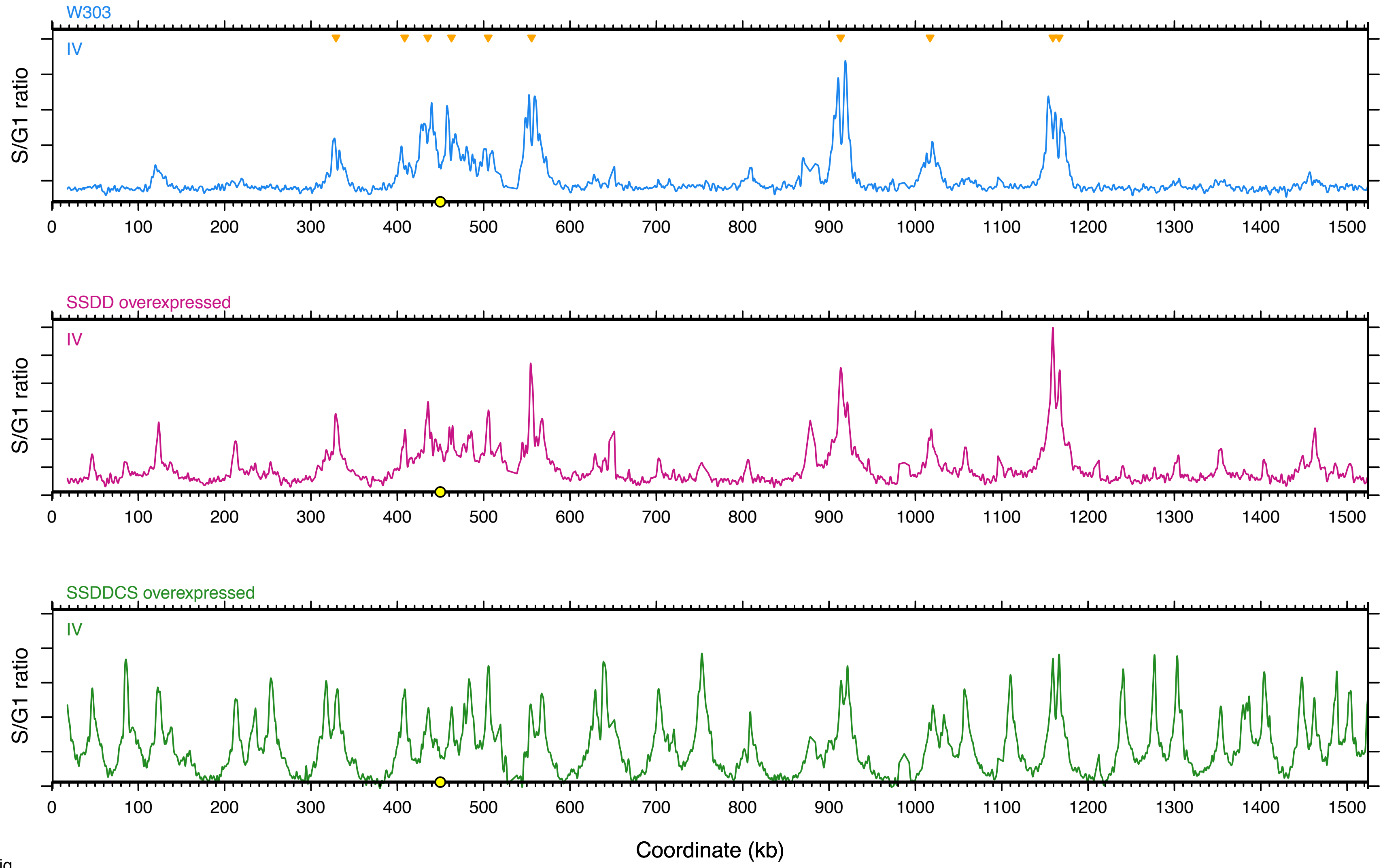

Lynch S12 Fig

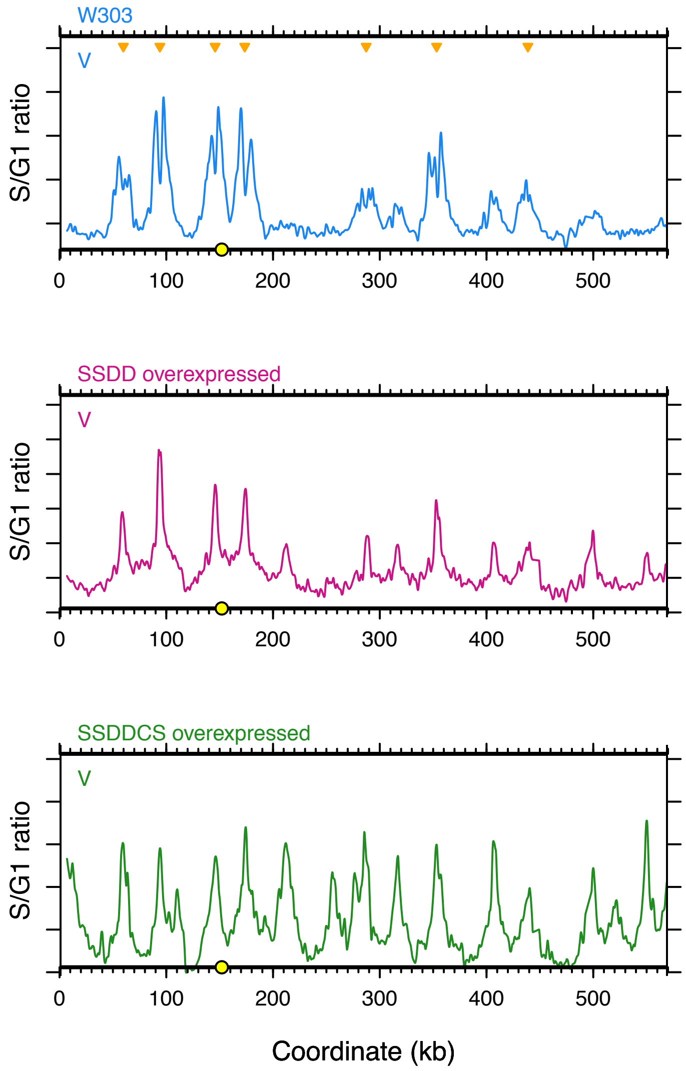

Lynch S12 Fig

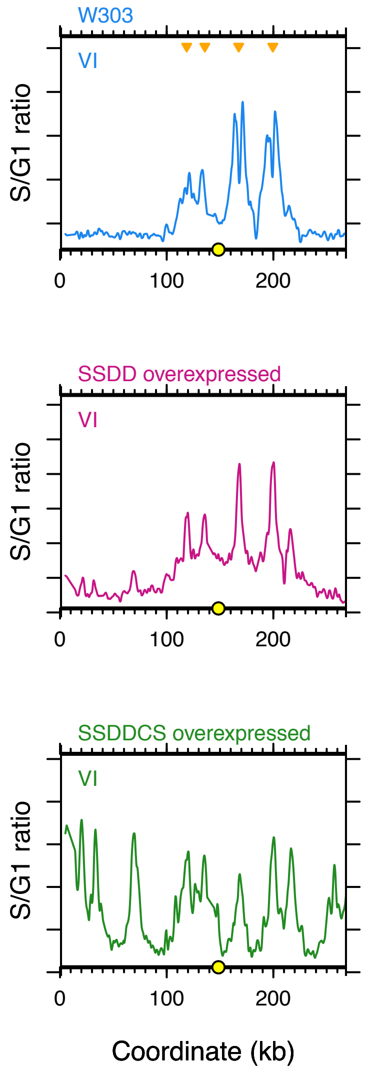

Lynch S12 Fig

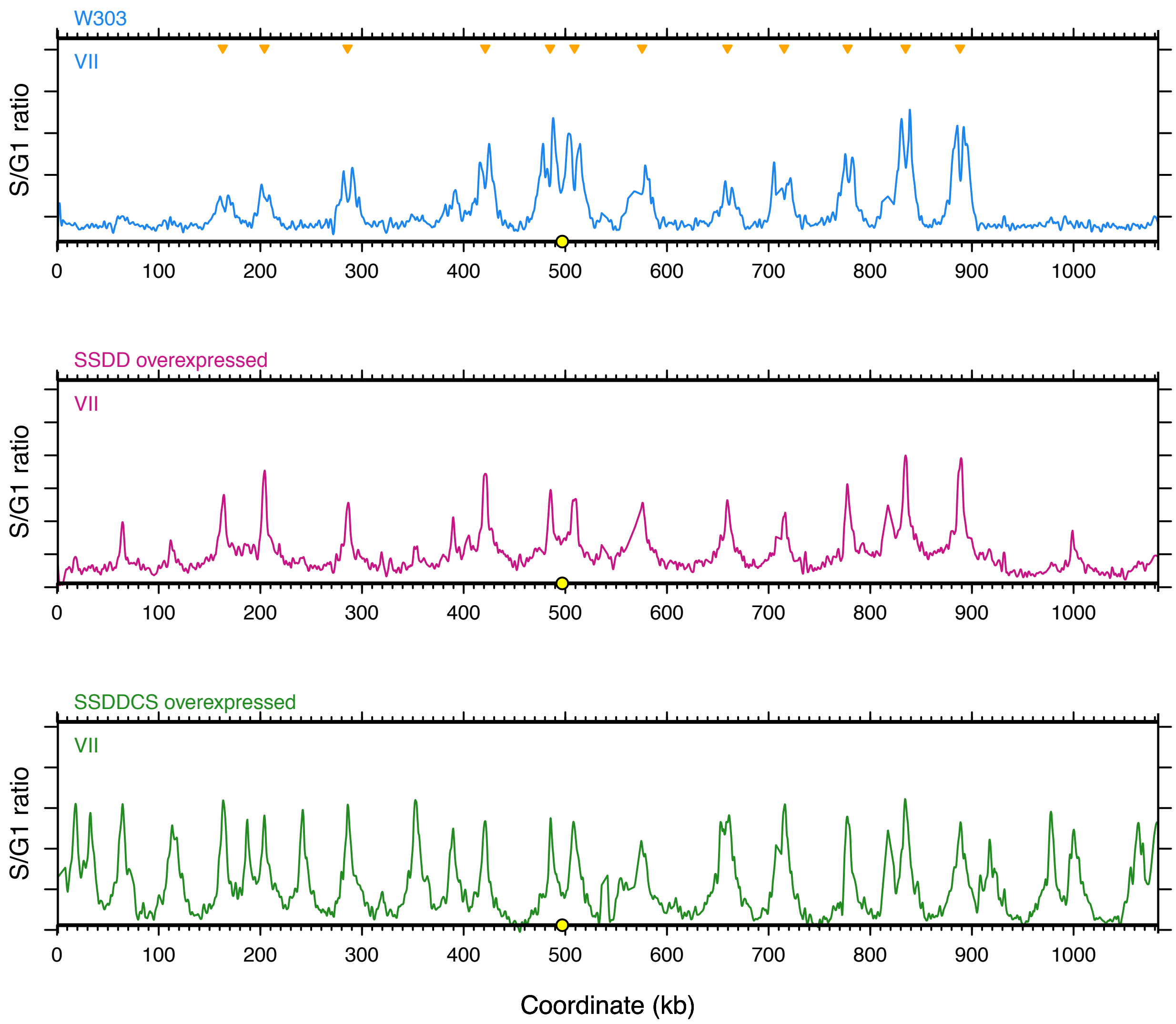

Lynch S12 Fig

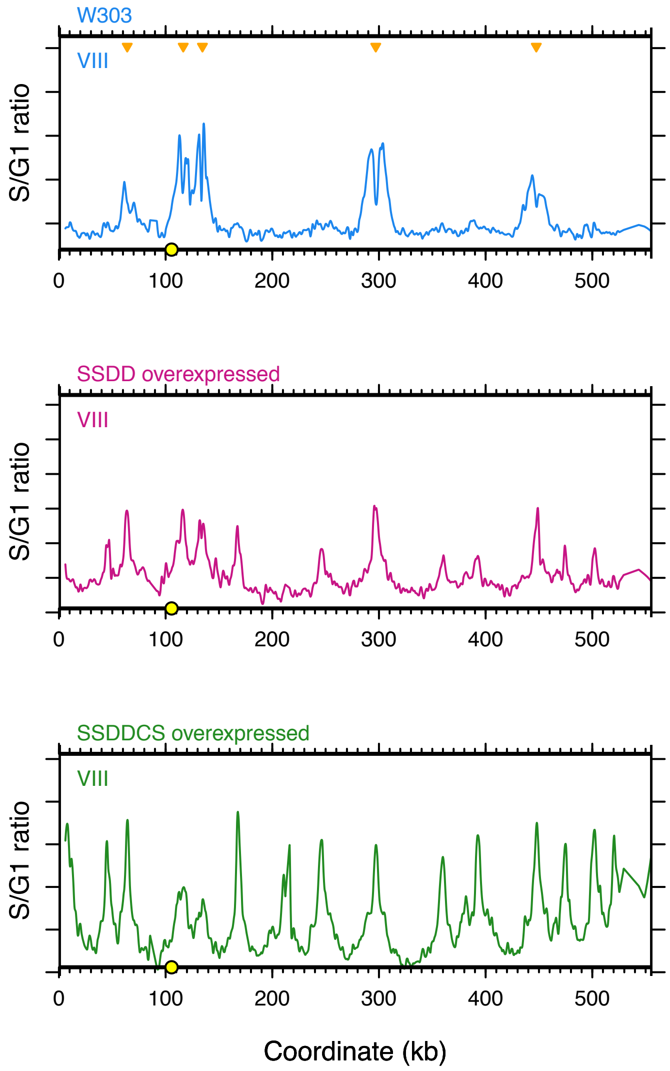

Lynch S12 Fig

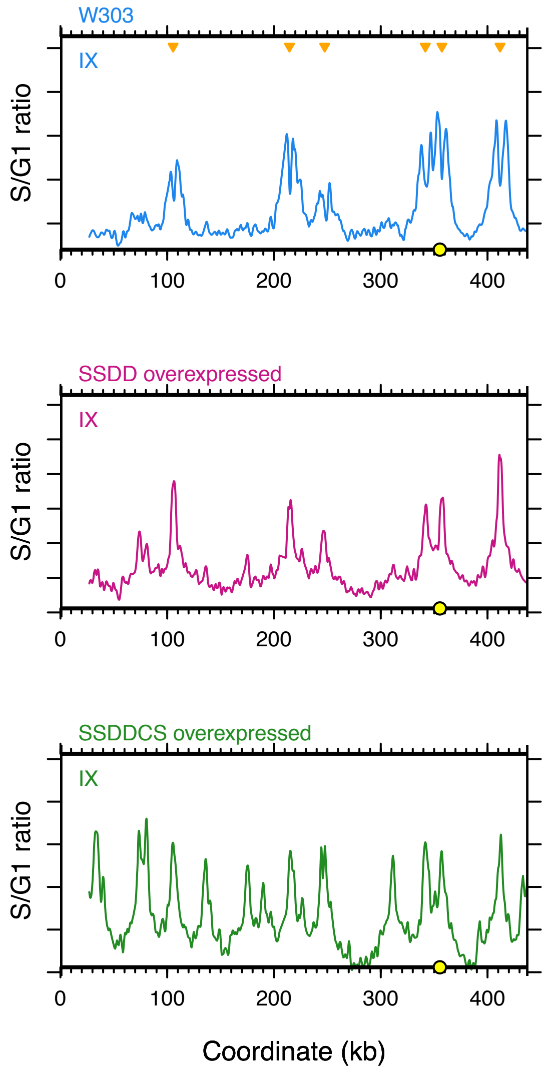

Lynch S12 Fig

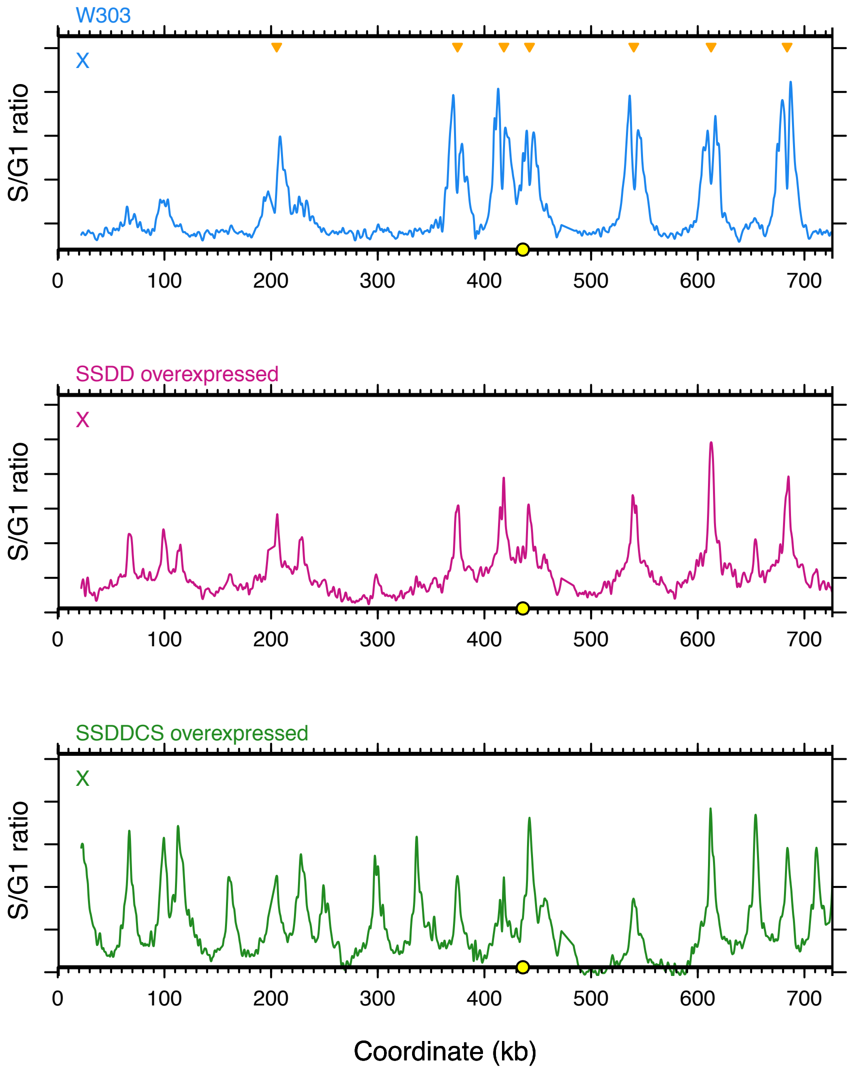

Lynch S12 Fig

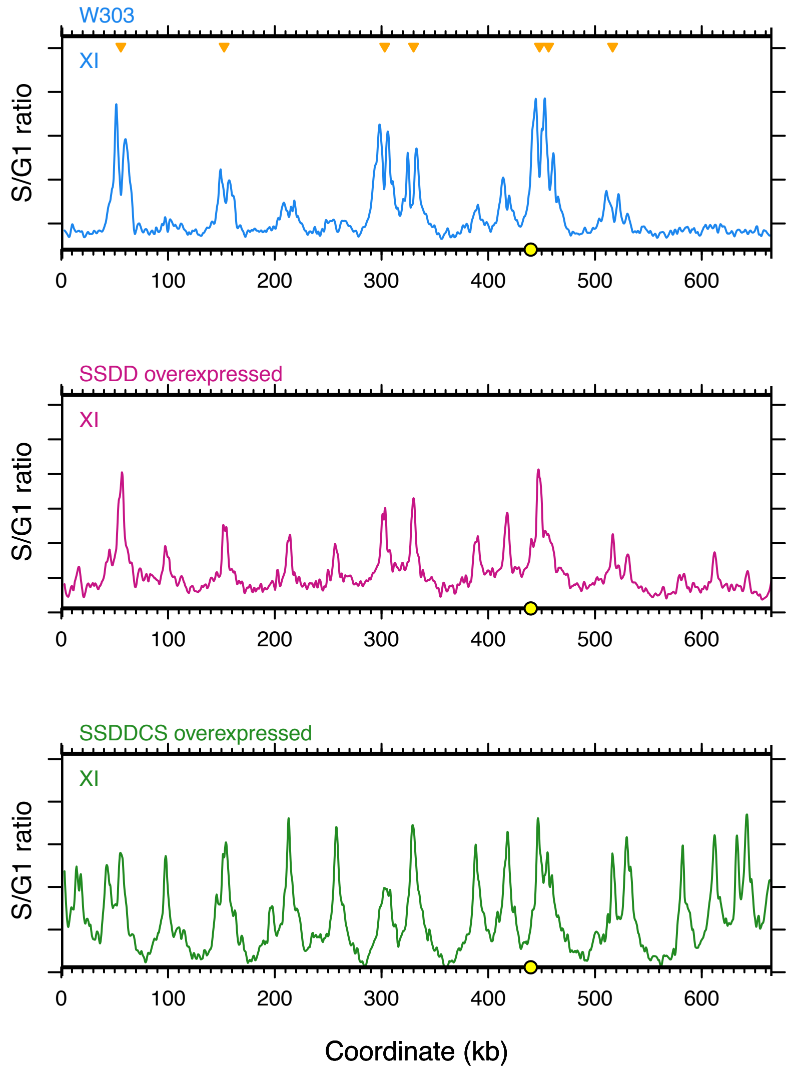

Lynch S12 Fig

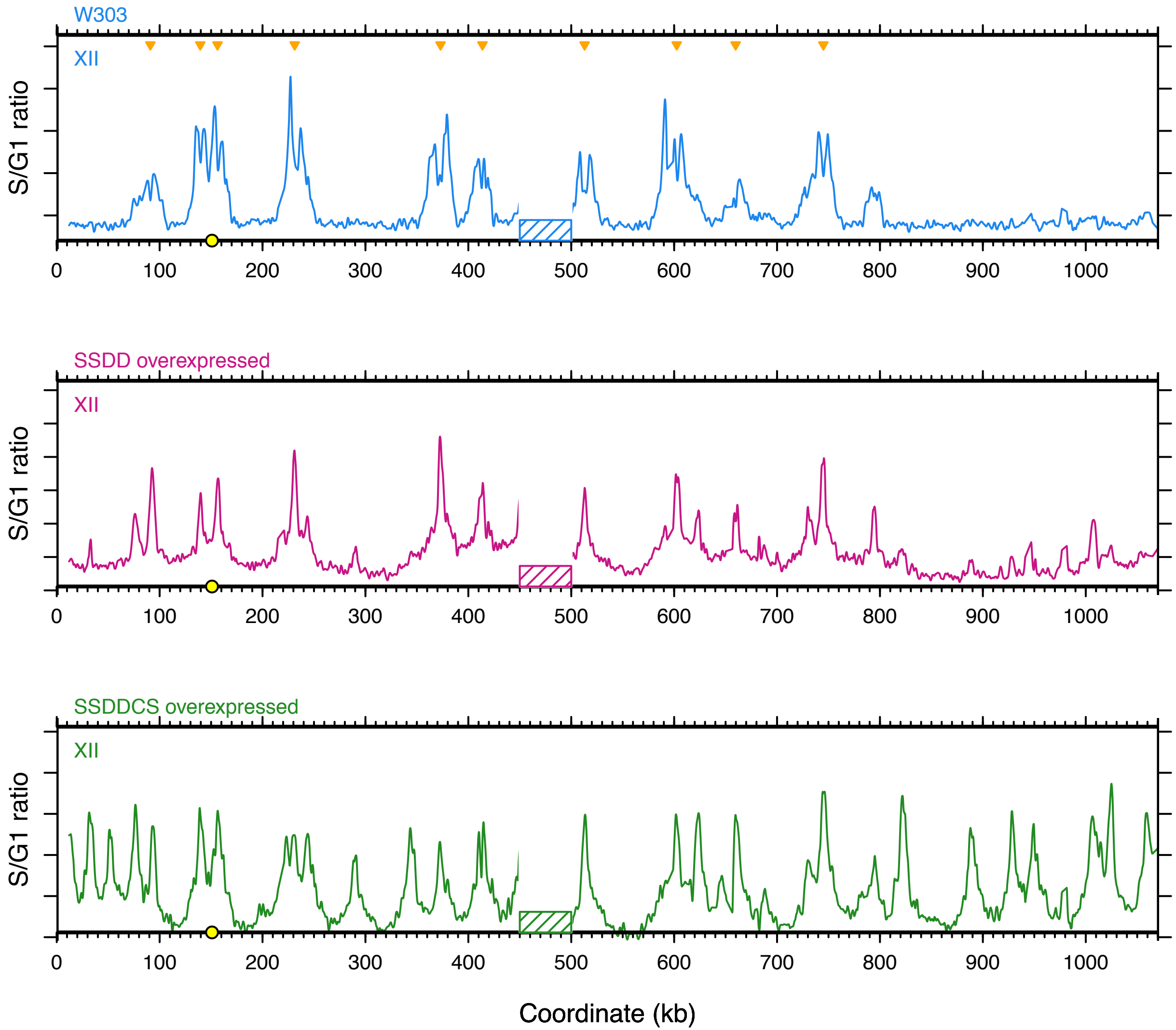

Lynch S12 Fig

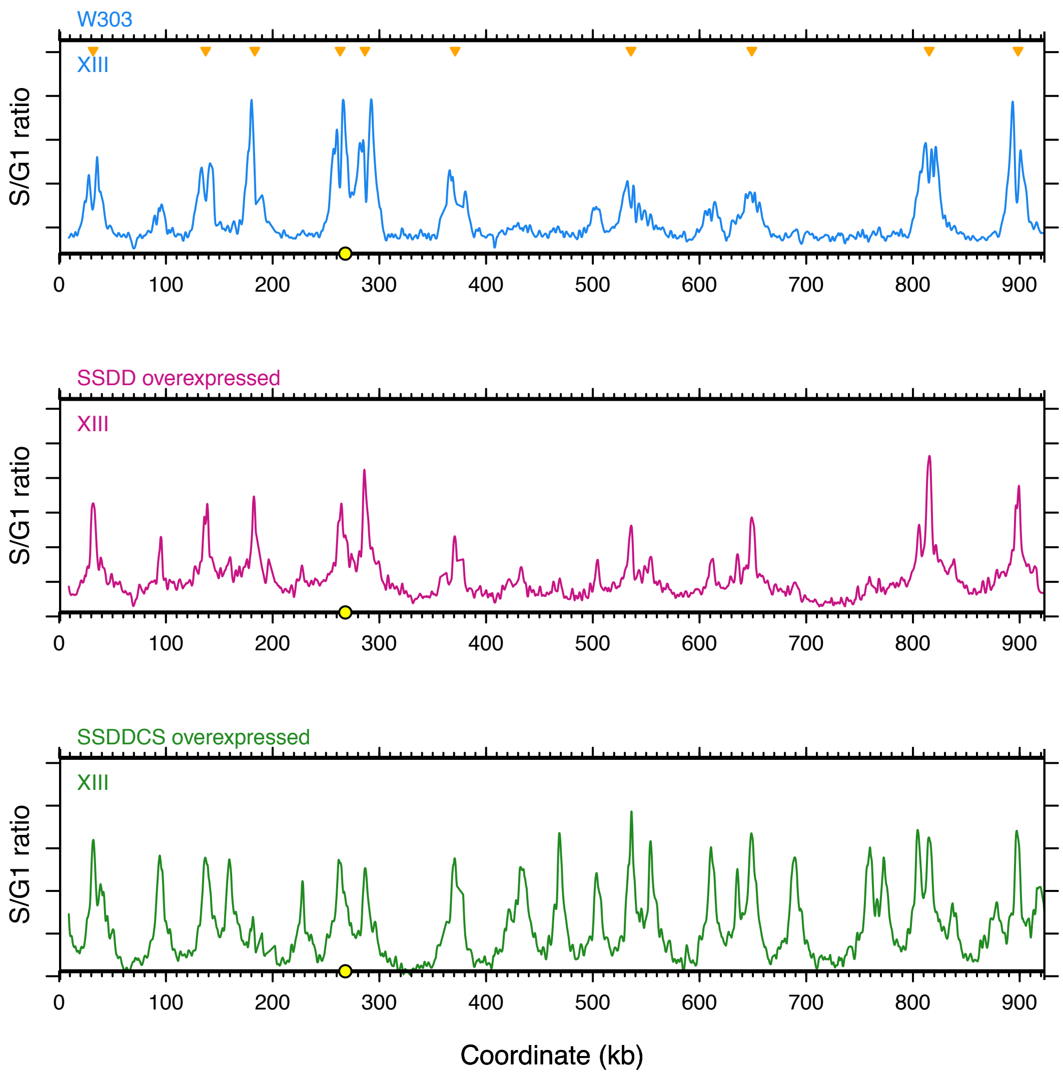

Lynch S12 Fig

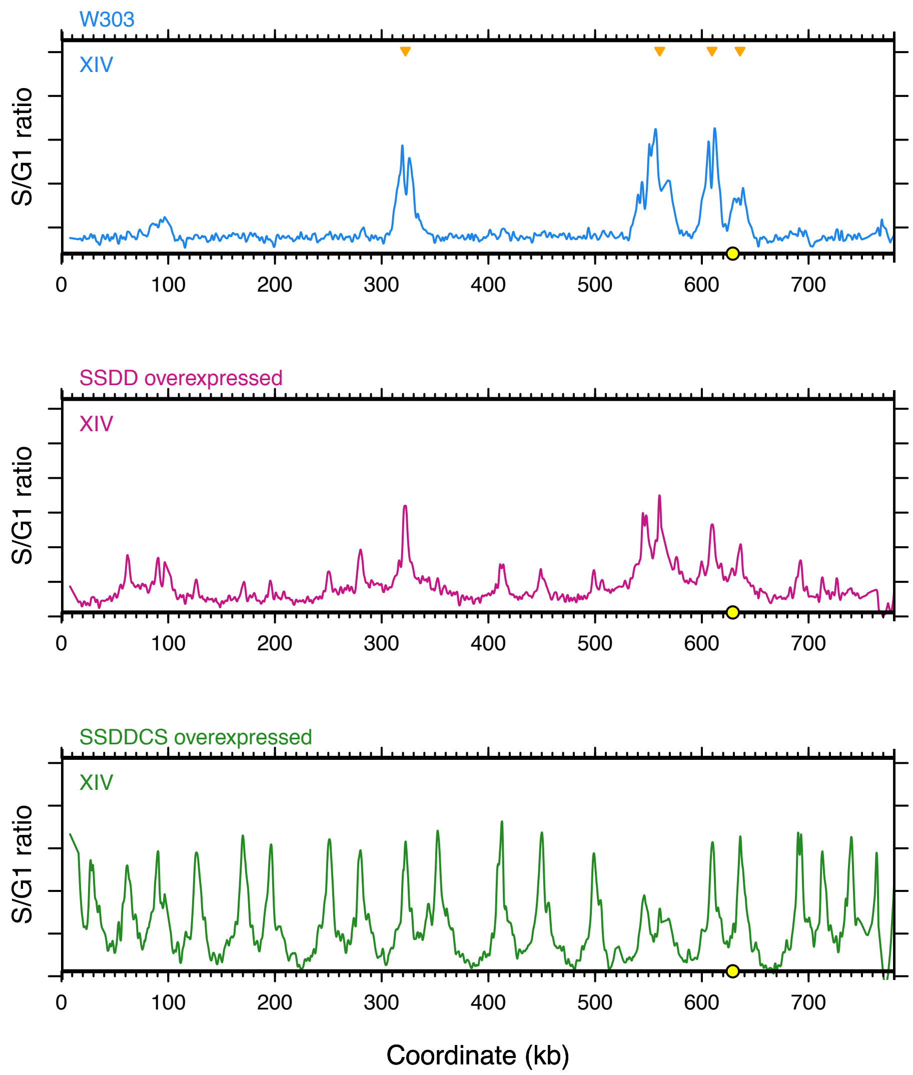

Lynch S12 Fig

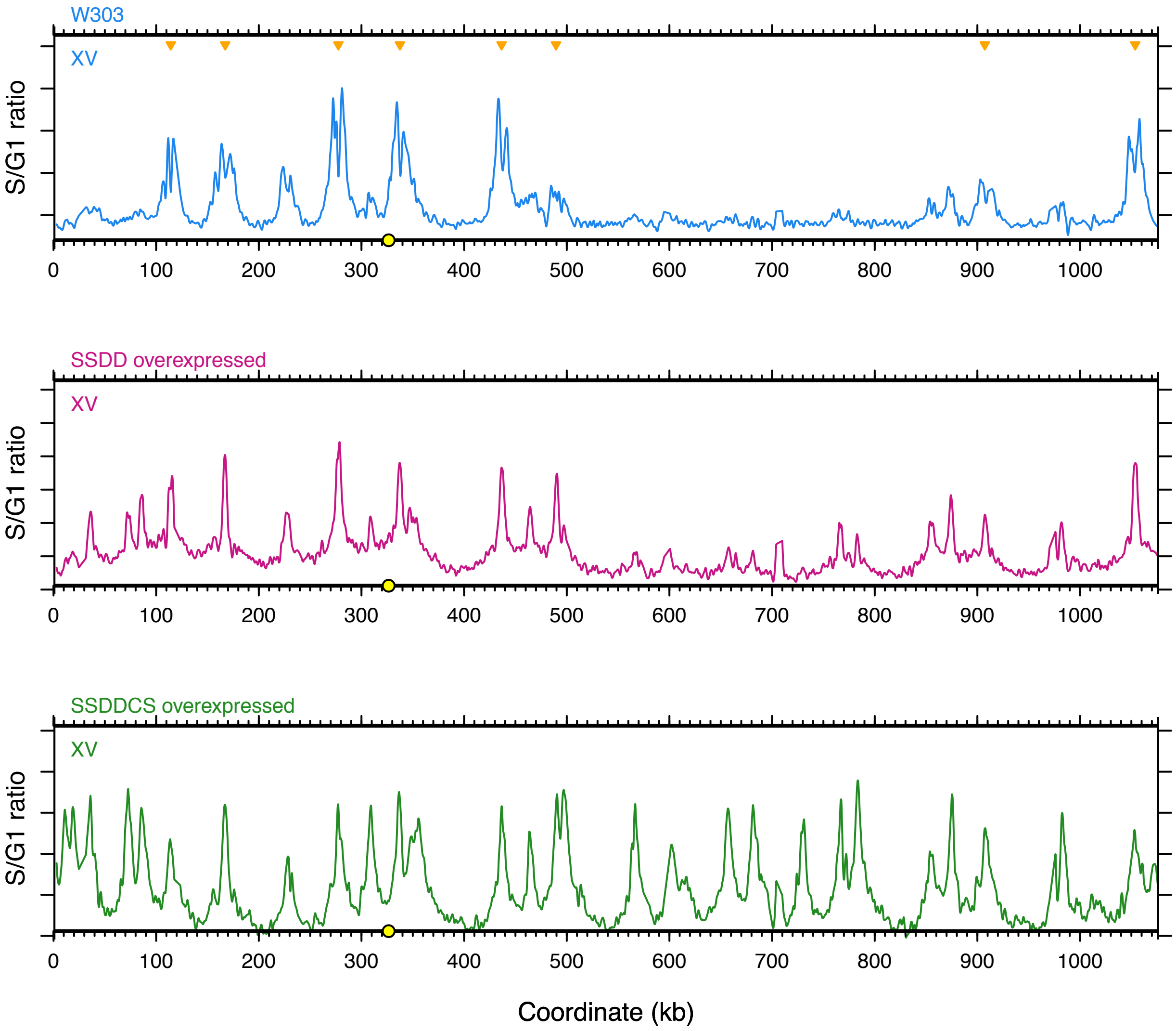

Lynch S12 Fig

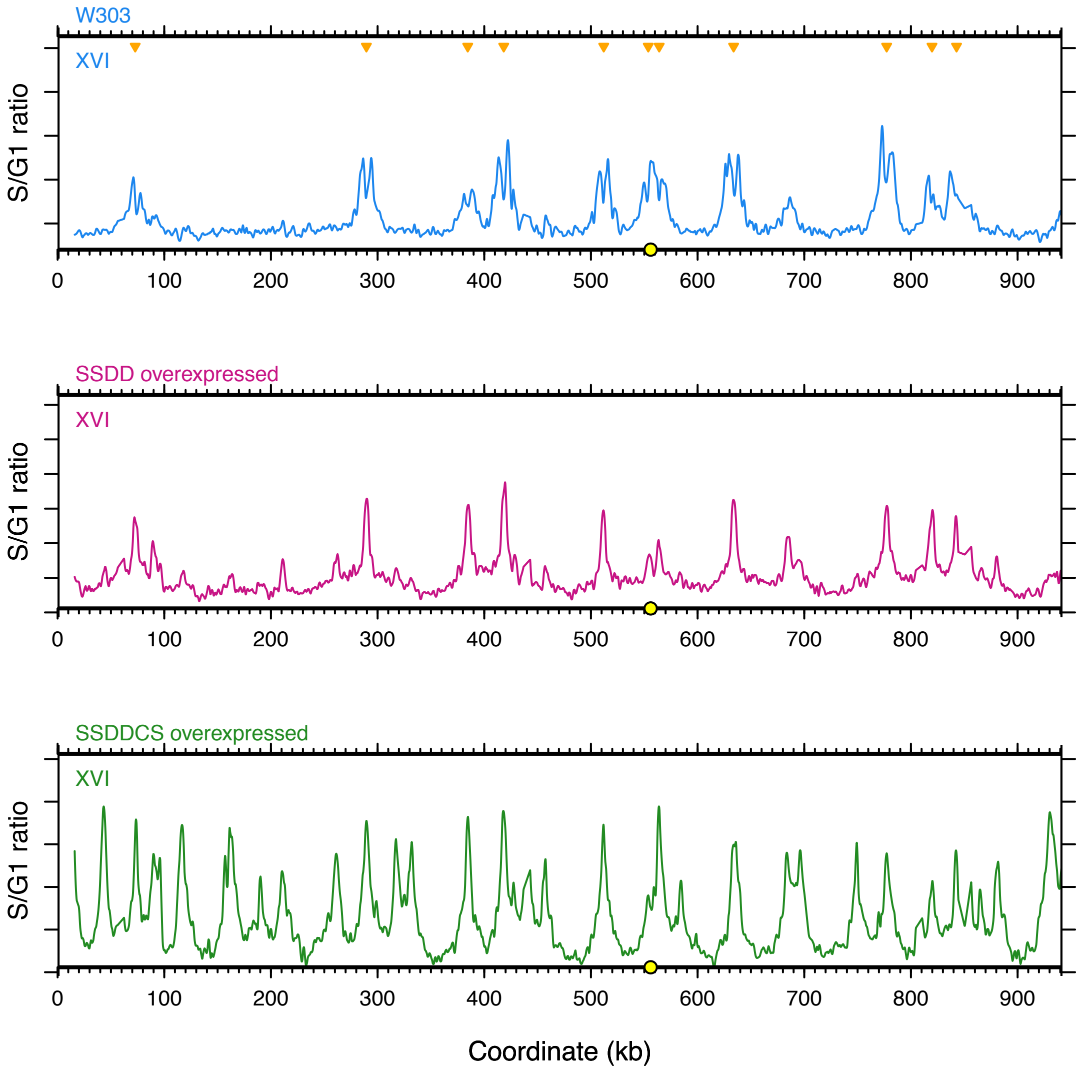

Lynch S12 Fig
